# Intelligent wireless theranostic contact lens for electrical sensing and regulation of intraocular pressure

**DOI:** 10.1038/s41467-022-29860-x

**Published:** 2022-05-17

**Authors:** Cheng Yang, Qianni Wu, Junqing Liu, Jingshan Mo, Xiangling Li, Chengduan Yang, Ziqi Liu, Jingbo Yang, Lelun Jiang, Weirong Chen, Hui-jiuan Chen, Ji Wang, Xi Xie

**Affiliations:** 1 State Key Laboratory of Optoelectronic Materials and Technologies, Guangdong Province Key Laboratory of Display Material and Technology, School of Electronics and Information Technology, Sun Yat-Sen University, Guangzhou, 510006 China; 2 State Key Laboratory of Ophthalmology, Zhongshan Ophthalmic Center, Sun Yat-Sen University, Guangzhou, 510006 China; 3 Department of Cardiology, the First Affiliated Hospital of Jinan University, Guangzhou, 510630 China; 4 School of Biomedical Engineering, Sun Yat-Sen University, Guangzhou, 510006 China; 5 The First Affiliated Hospital of Sun Yat-Sen University, Sun Yat-Sen University, Guangzhou, 510006 China

**Keywords:** Biomedical engineering, Electrical and electronic engineering, Diagnosis, Biotechnology, Sensors and probes

## Abstract

Engineering wearable devices that can wirelessly track intraocular pressure and offer feedback-medicine administrations are highly desirable for glaucoma treatments, yet remain challenging due to issues of limited sizes, wireless operations, and wireless cross-coupling. Here, we present an integrated wireless theranostic contact lens for in situ electrical sensing of intraocular pressure and on-demand anti-glaucoma drug delivery. The wireless theranostic contact lens utilizes a highly compact structural design, which enables high-degreed integration and frequency separation on the curved and limited surface of contact lens. The wireless intraocular pressure sensing modulus could ultra-sensitively detect intraocular pressure fluctuations, due to the unique cantilever configuration design of capacitive sensing circuit. The drug delivery modulus employs an efficient wireless power transfer circuit, to trigger delivery of anti-glaucoma drug into aqueous chamber via iontophoresis. The minimally invasive, smart, wireless and theranostic features endow the wireless theranostic contact lens as a highly promising system for glaucoma treatments.

## Abstract (web summary)

Towards intelligent treatment for glaucoma, here authors demonstrate integrated wireless theranostic contact lenses for in situ electrical sensing of intraocular pressure and on demand anti-glaucoma drug delivery.

## Introduction

Intelligent point-of-care electrical platforms that could provide real-time health assessment and medical intervention would greatly relieve many acute and stubborn diseases^1–6^. Among the diseases, glaucoma and its combined ophthalmic diseases can cause irreversible vision loss in patients^7^, which is often deteriorated by the elevation of intraocular pressure (IOP) due to abnormal circulation of aqueous humor^7–9^. Since IOP varies associated with human activities and circadian rhythm^10^, it needs long-term and continuous tracking to analyze the critical IOP fluctuations for identifying optimal therapeutic conditions^11^. At present, many types of ophthalmotonometers (e.g., indentation tonometry, applanation tonometry, rebound tonometry, and dynamic contour tonometry) have provided snapshot measurements of IOP for glaucoma diagnosis in hospitals^12^, yet the operations generally require trained clinicians and fail to collect many critical IOP fluctuation^13^. On the other hand, clinical medicine administrations for glaucoma treatments have been relying on topical drug delivery via eye drops to reduce IOP for suspending the deterioration of vision that glaucoma caused^8,12,14^. However, conventional drug deliveries into the anterior chamber remain challenging (low intraocular bioavailability, inevitable side-effects, and poor patient adherence) due to the diffusion barriers of cornea^15^, and lack the possibility of integration with smart biodevices for on-demand drug delivery. Especially for acute angle-closure glaucoma featured with the sudden rise of IOP^16^, it is usually accompanied by headache, nausea, and vomiting that hinders manual self-administration by patients^8^, while the delayed reduction of IOP will inevitably cause ischemic infarcts and damage optic nerve^12^ (Supplementary Information S1).

Contact lens, an ideal platform contacted with the human eye intimately^17,18^, has been exploited as wearable devices for physiological measurements^2,19–22^. In recent decades, contact lens-based IOP sensors integrated with resonant circuits, microfluidic chips, piezoresistive, and photonic crystal technologies have emerged^13,19,20,23–26^. For example, Park et al. developed a colorimetric contact lens for IOP reading based on a photonic crystal sensor coupled with a microhydraulic strategy to amplify sensitivity^20^. To acquire electrical signals of IOP, Kim et al. demonstrated graphene/Ag nanowires and silicon strain sensors-based contact lens that could detect IOP with high sensitivities^19,23,27^. Besides, controlled ocular drug deliveries mediated via contact lens devices have employed versatile strategies including thermal-responsive, enzyme triggering, and hydrogel layer-controlled drug release^25,26,28,29^. To reduce the burst release of drugs from devices, Cakmak et al. fabricated a multi-diffusion layers-based contact lens that could achieve stable ophthalmic drug administration with a constant rate^29^. However, medicine's permeabilities into an aqueous chamber by these passive diffusion methodologies are generally compromised due to the physiological barriers of an eye, especially the frequent tear clearance and the tightly packed corneal epithelium cells^30^. While most of the existing strategies for glaucoma applications focus on either sensing or delivery separately, integrated wireless electrical systems for IOP monitoring and regulation are highly desirable to treat glaucoma, yet are rarely developed due to challenges (Supplementary Information S2).

Closed-loop theranostic systems on flexible patches have recently been developed to automatically monitor biomarkers, and respond rapidly to treat these complications^1,3,4,31^. However, in contrast to patch devices worn on the skin, theranostic systems based on contact lens confront several complicating challenges due to its nature of the limited size and the requirement of wireless operations. First, the contact lens is a flexible, lightweight, curved, and ultrathin device with an extremely limited area^32,33^. It is highly challenging to install an intricate theranostic system composited by multi-modules on a contact lens, which is less compatible with standard 2D micro/nano-fabrication routes. Second, contact lens devices need to operate wirelessly to promote patients’ comforts^34^, yet the potential cross-coupling between wireless sensing and delivery modulus on a limited device area would interfere with their individual operations. Third, simultaneous satisfaction of detection sensitivity and on-demand drug delivery on a single device are also difficult, since the limited space of contact lens would restrict the sizes of sensor or delivery module to achieve effective operations.

**Fig. 1:**
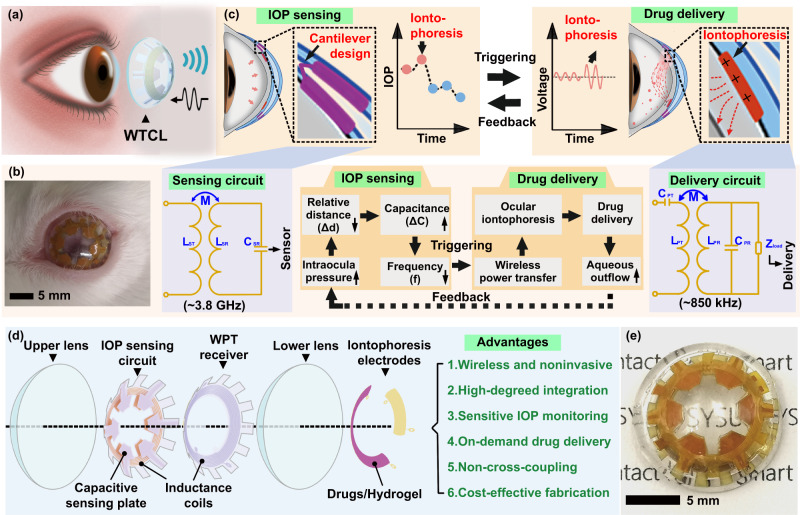
Schematic of the WTCL for real-time and in situ IOP monitoring and drug administration. **a** Schematic of the WTCL for wireless IOP monitoring and administration. **b** Photograph of WTCL worn on the eyes of a live rabbit. **c** Schematic of wireless operation for the purpose of IOP monitoring and on-demand medicines administration in a minimally invasive manner. The soft device, engineered as a double-layer contact lens structure, was integrated with an LCR and a WPT receiver circuit. These modules were wirelessly connected to an external integrated antenna that could record the IOP signal and trigger iontophoresis for drug delivery if needed. Insert figures respectively highlight critical IOP sensing and drug delivery unit. **d** Structure of the WTCL in an exploded view. **e** Optical image of the WTCL.

In this work, an integrated wireless theranostic contact lens (WTCL) was developed, for in situ IOP monitoring and electrically triggered drug administration in high-risk IOP conditions (Fig. 1a). The WTCL employed a highly compact structural design and circuits layout, which enabled high-degreed integration of IOP sensing and on-demand delivery modulus on the curved and limited surface of the contact lens without vision blockage (Fig. 1b). The IOP sensing modulus possessed a unique cantilever configuration of the LCR circuit, where each capacitive sensing plate sandwiching ultra-soft air dielectric film could ultra-sensitively respond to the IOP changes, producing detectable resonant frequency signals for wireless recording. The drug delivery modulus utilized an efficient wireless power transfer (WPT) circuit to drive coated anti-glaucoma drugs to migrate into the aqueous chamber via iontophoresis, which offered an electrical switch for drug delivery and enhancement of drug permeation across the cornea (Fig. 1c). The specialized design of the wireless sensor and WPT receiver enabled channel separation via different operational frequencies without cross-coupling, ensuring the individual functions of modulus in an integrated system. The minimally invasive, smart, wireless, and theranostic features of the WTCL endowed this platform as a highly promising tool for facilitating IOP administration and treatment acute angle-closure glaucoma.

## Results

### System design and fabrications of the WTCL

A soft contact lens conformally interfaced with the cornea could effectively deform to transduce the expansion of the corneal limbus to the integrated sensor circuit when IOP increases, and could locally exert external stimulations (e.g., electricity or chemicals) on the cornea. Double-layer lens structure, a typical design acceptable for contact lens devices^34,35^, was adopted for fabricating WTCL to benefit the integration of the LCR circuit and drug administration module (Fig. 1d) on the extremely limited space of contact lens (Fig. 1e). The air film sandwiched between two layers of the lens combined with an LCR circuit characterized by a cantilever structure formed the IOP transducer that could detect pressure fluctuations and transmit it wirelessly^23,36^. At high-risk IOP conditions (IOP >21 mmHg), WPT triggered iontophoresis enables in situ drug administration effectively. Key advantages of this device contain the following: (1) the soft, lightweight, re-usable, and minimally invasive features as well as wireless operations are compatible with the contact lens platform; (2) Compact structural design and circuits layout enable high-degreed integration of IOP monitoring and on-demand delivery modulus in a limited area without vision blockage; (3) Rational circuit designs enabled sensitive IOP monitoring by unique cantilever sensor structure, on-demand, and effective ocular drug delivery via iontophoresis, independent wireless channel without cross-coupling via frequency separation; (4) the cantilever capacitive sensor is sensitive to pressure, which allows drug delivery circuits to integrate into limited space without blocking IOP monitoring. Slight distance displacement or angular displacement of the capacitive plates driven by IOP could readily induce significant electrical signals. Due to the cantilever design that an ultra-soft air layer is present between the capacitive plates, the displacement of capacitive plates could be sensitively responsive to the pressure even in the case that the pressure could be partially buffered by the delivery coils on top of the sensor (Fig. S2). (5) The theranostic system with an entirely electrical interface is beneficial for signal collection, processing, feedback, and transmission, as well as programmable on-demand drug administration. (6) Fabrication of the device is compatible with the existing large-scale and cost-effective manufacturing process, emphasizing its potential for widespread applications.

**Fig. 2:**
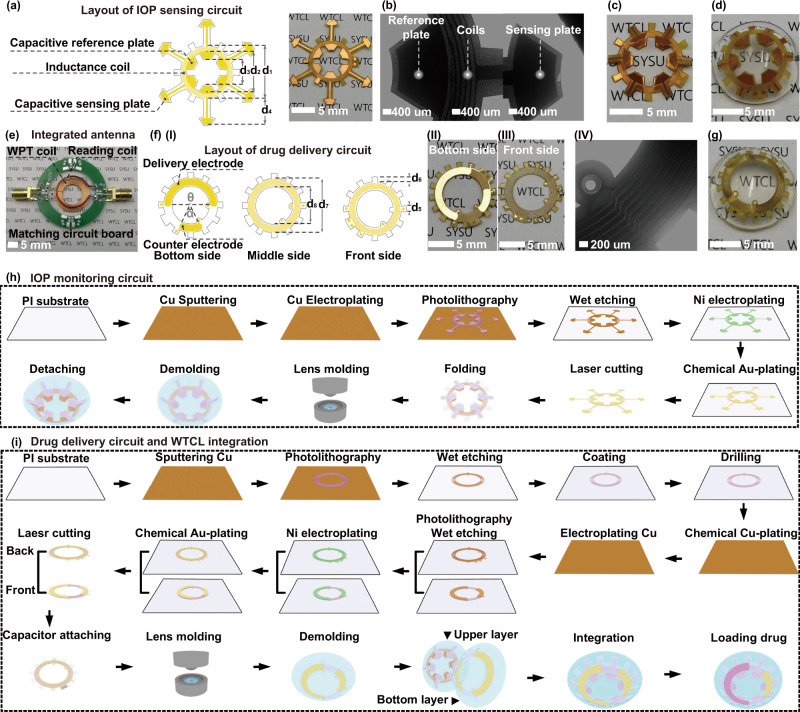
Schematic illustration of the WTCL’s design and fabrication process. **a** The snowflake-shaped layout design and the photograph of the sensing circuit. **b** The microscopic image of the reference plate, coils, and sensing plate deployed on the sensing circuit. The photograph of (**c**) the folded sensing circuit and (**d**) the upper layer of contact lens. **e** Image of the integrated antenna. **f** (I) The flower-shaped layout design, (II) bottom surface, (III) front surface images, and (IV) microscopic image of the drug delivery circuit. **g** The photograph of the bottom layer lens integrated with the drug delivery circuit. Illustration of the fabrication process of (**h**) IOP monitoring circuit, **i** drug delivery circuit and the device integration. The fabrication of the sensing and delivery modulus employed a printed circuit process coupled with a cast-molding method.

The IOP monitoring circuit employed a unique snowflake-shaped layout design (Fig. 2a), where each capacitive sensing plate (totally six plates) was then aligned with the reference plate (Fig. 2b) by folding to form a cantilever configuration (Fig. 2c). The reference plates and five coils of inductance were embedded in the upper lens (Fig. 2d), while the dangling sensing plates contacted the front surface of the lower lens, with a dielectric air film between the reference and sensing plates forming a variable capacitor (Fig. S2). The capacitance combined with the inductance coil formed an LCR circuit for wirelessly IOP monitoring. The deformation of corneal curvature caused by increased IOP compressed the thickness of the air dielectric layer (Δd), leading to the rise of capacitance (C_SR_) and reduction of the resonant frequency of the LCR circuit that could be recorded by reading coil of an integrated antenna (Fig. 2e) wirelessly^23,36^. Due to the ultra-soft (ultra-low elastic modulus and zero viscoelasticity) feature of the sandwiched air film, the variable capacitors formed by cantilever configuration can ultra-sensitively respond according to the change of pressure (Fig. S2). The cantilever design effectively avoids the issues of redundant serial capacitors, and complicated device fabrication process, especially the wire bonding step that have been encountered in previously reported strategies^36,37^. On the other hand, the drug delivery circuit utilized a flower-shaped layout design (Fig. 2f) that enabled robust interlocking mechanically between the flexible circuit and the lower layer of the contact lens (Fig. 2g)^38^. The front side of the circuit embedded in the lower lens possessed coils (Fig. 2f-IV and Fig. S2) connected with a chip capacitor for wireless power harvest, while the drugs-coated iontophoretic electrodes on the bottom side of the delivery circuit were exposed and be in contact with the cornea. Anti-glaucoma drugs, brimonidine, was loaded in a hydrogel layer coated on the iontophoretic electrode, which could be delivered into the aqueous humor via wirelessly iontophoresis to reduce IOP. The iontophoresis not only offered a non-mechanical switch for drug delivery in a low-power consumption manner but also facilitated drug penetration across the cornea via electrophoresis effects^39^. The rational design of the wireless sensor and WPT receiver at different operational frequencies (~3.8 GHz and ~850 kHz, respectively) enabled channel separation for individual functions. The double-layer lens design enabled a compact structure to accommodate multiple electronic modulus positioned in the rim region of the contact lens, hence possessed an open vision window larger than the pupil’s size without blocking the views of wearers.

The fabrication of the sensing and delivery modulus employed a printed circuit process coupled with a cast-molding method. For the sensing module, Cu (~100 µm) was electro-deposited on a flexible polyimide (PI) substrate and patterned via photolithography and wet etching, followed by covering with Ni/Au to improve the biocompatibility of electrodes. The flexible substrate was cut by laser according to the snowflake-shaped circuit design, and then was folded and embedded into the upper polydimethylsiloxane (PDMS) lens via cast-molding technique (Fig. S4), where each folded sensing plate was detached from the contact lens to form a cantilever configuration (Fig. 2h). For the delivery module, similar to the sensing module, Cu was electro-deposited on PI substrate and patterned as coils features via photolithography, which was then removed by wet etching to form the WPT receiver. The second layer of PI was spinning coated on top of the coils, and another Cu layer was prepared according to the iontophoretic electrode pattern, which was connected to the coils at the bottom side by through-holes. The electrodes were further covered with Ni/Au, and capacitors were soldered onto the front side of the circuit to tune the WPT operation frequency, followed by embedding the circuit into the lower PDMS lens via cast molding. The delivery electrode of the lower lens was coated with a thin layer of drugs-loaded hydrogel and assembled with the upper lens to form the final WTCL (Fig. 2i). The compact layout and double-layer lens design enabled sensors and WPT receiver to be embedded inside the contact lens, avoiding direct contact of these components to the ocular surface that might cause potential irritations to the eye. The WTCL fabrication was compatible with the commercial printed circuit board process, indicating the potential for large-scale manufacturers of this biomedical device. For experiments, an integrated antenna (Fig. 2e) consisting of concentrically aligned IOP reading coils and a WPT coil soldered on a matching circuit board was fabricated, which could collect the output signals from the wireless sensor and transfer power to the WPT receiver of the WTCL. Related parameters of the WTCL’s double contact lens structure were illustrated in Fig. S5.

### Ex vivo performance of wireless IOP monitoring

**Fig. 3:**
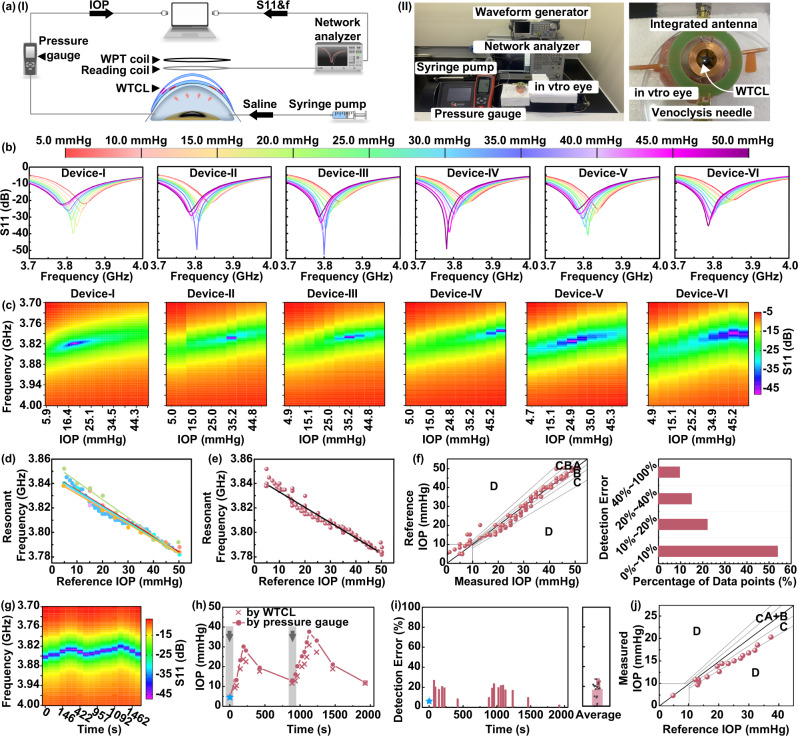
IOP sensing performance of the WTCL. **a** (I) Schematic and (II) experimental setup of the wireless IOP sensing experiments. **b** The reflection spectra of six representative WTCL devices worn on porcine eyeball at different IOP. **c** The results of the S11 values at different frequencies and IOP conditions in (**b**) were plotted as a heatmap diagram, where the value of S11 exhibited a linear pattern in the frequency-IOP heatmap. **d** Linear regression of resonant frequency versus IOP value of each WTCL device. **e** The averaged linear regression of resonant frequency versus IOP value of the six WTCL devices in (**b**). **f** Error grid analysis and statical analysis of the IOP sensing accuracy via WTCL. Region A, B, C, and D referred to errors <10, 10–20, 20–40, and >40%, respectively. **g** Heatmap plot of the reflection coefficients recorded during the continuous recording of IOP via WTCL. **h** Continuous IOP signals monitored by WTCL on ex vivo porcine eyeball. The calibration point using reference IOP was marked with blue asterisks. The black arrow referred to the time point of saline injections. **i** Statistical analysis of detection errors via WTCL compared to commercial pressure gauge at different time points. The calibration point was marked with blue asterisks. *N* = 17 data points. Data were presented as mean ± SD. **j** Error grid analysis of the continuous IOP sensing via WTCL. Region A + B, C, and D referred to errors <20, 20–40, and >40%, respectively. *N* = 17 data points.

The sensing performance of the WTCL was tested ex vivo using porcine eyeballs, where porcine eyeballs with similar features to human eyeballs have been widely employed in many physiological experiments. The IOP in the porcine eye was tuned by controlled infusion of saline solution into the anterior chamber via a microinfusion pump, with a pressure gauge to monitor the reference IOP. The IOP reading coil (diameter: 17 mm, turns: 1) of the integrated antenna connected to a network analyzer was positioned on top of the WTCL to monitor the resonance frequency (Fig. 3a). The static sensing performance was conducted by a stepwise increase of IOP, while the resonance frequency of WTCL at each IOP condition was recorded. The reflection spectra of six representative WTCL devices worn on the porcine eyeball at different IOP (5–50 mmHg) were recorded and analyzed (Fig. 3b and Fig. S6), where the resonant frequency of the IOP monitoring module was found to shift to the lower frequency at higher IOP. The return loss (S11) values at different frequencies and IOP conditions were plotted as heatmap diagrams, where the S11 value exhibited a linear pattern in the frequency-IOP heatmaps (Fig. 3c). The relation between resonance frequency and IOP of each device was analyzed (Fig. 3d and Fig. S7), which was revealed to be in an inversely linear profile (average R-Square = 0.976 ± 0.015). Theoretically, the resonant frequency is inversely related to the capacitance according to the LCR circuit equation $f={\left(2\pi \sqrt{{LC}}\right)}^{-1}$. Our results were consistent with the theoretical prediction in that the increase of IOP would reduce the distance between the capacitance electrodes, hence led to the elevation of capacitance value and reduction of the resonant frequency. A theoretical model revealing the relations between the mechanical deformation of contact lens and the shifting of resonant frequency was established with the COMSOL simulation method, which verified that the increase of IOP pressure leads to the reduction of resonant frequency (Fig. S8 and Table S2).

Noted that the linear relations of all the six devices overlapped well (normalized slope variation <15%) with each other (Fig. S9), indicating the reliability and repeatability of the fabricated device using our design.

A universal standard curve between resonant frequency and IOP was established by averaging all the six linear curves obtained from the measurements on the six representative devices (Fig. 3e), where this standard curve could be employed to calculate detected IOP based on the measured resonant frequency. The results suggested that the IOP sensors in the WTCL possessed sufficient sensitivity of 1.28 ± 0.09 MHz/mmHg, which was superior or comparable to other wireless IOP sensors (Table S3). This was likely due to the specific cantilever design of the sensor, where the ultra-soft air film sandwiched between the sensing and reference plates was mobile so that the variable capacitors formed by the cantilever configuration could respond to the change of pressure in a sensitive manner. The linear range of WTCL was wider than 5–50 mmHg, which were desirable for IOP monitoring applications. The measured IOP values by the six WTCL devices were derived from the recorded values of resonant frequency, and compared to the reference IOP measured by pressure gauge for analyzing the sensor’s static accuracy via error grid analysis (Fig. 3f). The percentage of data points at different error ranges was quantified, where >50% recording was found to be within error <10%, and >75% recording was found to be within error <20%. The continuous recording of IOP via WTCL was also examined by measuring the resonant frequency and reference IOP via pressure gauge, respectively, where the saline solution was injected into the anterior chamber at *t* = 0 s and 883 s intending to induce IOP spikes (Fig. 3g). The measured resonant frequency (Fig. S10) was calculated into IOP according to the WTCL’s averaged standard curve, and the results were calibrated (Fig. S11). Considering the possible batch variations of devices and porcine eyeballs in experiments, the detected IOP results via WTCL were calibrated (Supplementary Information S4.7) by a pressure gauge-measured data point at *t* = 0 s (indicated with a blue star in Fig. 3h). The injections of saline induced rising of IOP, followed by slight IOP declines potentially due to the gradual leakage of solution from the eyeball, which were all consistently recorded by both WTCL and pressure gauge. The dynamic recording accuracy of WTCL at each time point was analyzed (Fig. 3i) and plotted via an error grid analysis (Fig. 3j), and the average error was found to be 16.49 ± 7.58% with all the errors below 30%. The above results demonstrated the WTCL possessed sufficient sensitivity, linear region, and reliability that were desirable for IOP monitoring.

### WPT performance characterization

**Fig. 4:**
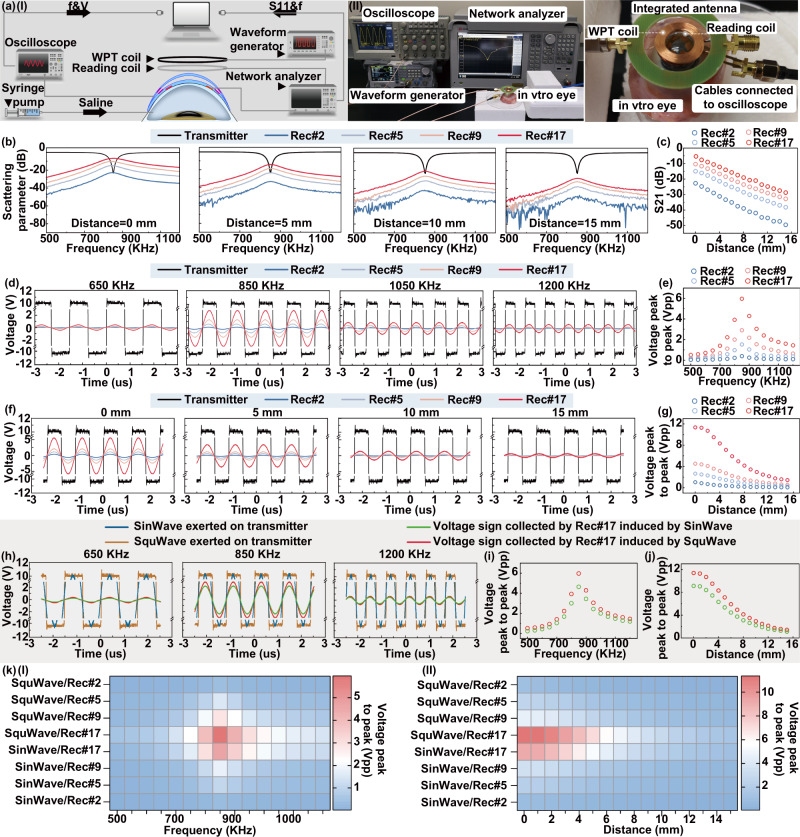
WPT performance of the WTCL. **a** (I) Schematic and (II) experimental setup of the WPT experiments. **b** Reflection coefficient spectra (S11 and S21) were recorded from four receivers at different radiation distance. **c** The S21 recorded by the four receivers at 850 kHz were plotted as a function of radiation distance. **d** Alternating voltage signals collected from four receivers wirelessly under different frequency radiation at 20 Vpp applied on the transmitter, and (**e**) Wirelessly transferred alternating voltage waveforms of four receivers under different radiation distance at 20 Vpp applied on the transmitter, and (**f**) the Vpp were plotted as a function of frequency and (**g**) as a function of distance. **h** The wirelessly transferred voltage signals collected by Rec#17 were activated by SquWave or SinWave voltages at different frequencies, and the Vpp were plotted as a function of (**i**) frequency or (**j**) distance. **k** Heatmap plot summarized the Vpp recorded from four receive circuits under different voltage-transfer conditions, including the coupling frequency, the radiation distance, and the waveforms.

Magnetic resonance coupling-based WPT has been a competing technique for wireless bioelectronics due to its relatively high power transfer efficiency and resistance to environmental inference^40^. As the WPT device design and optimization process (Fig. S12), the resonant frequency of the transmitter and all receivers were designed to be at 1 MHz, to ensure the frequency was significantly separated from the resonant frequency of the IOP sensing module (~3.8 GHz). Based on our observation of ex vivo drug delivery in a preliminary experiment (as well as those we verified and showed in fluorescence microscopic images of rhodamine B absorbed in ocular tissue after ex vivo experiments on porcine eyes, iontophoresis with a frequency of ~850 kHz would produce the optimal drug delivery efficacy among the range of 0.65–1.2 MHz. To optimize coupling performance, four types of WPT receivers with 2, 5, 9, and 17 coils-design (namely Rec#2, Rec#5, Rec#9, and Rec#17, respectively) were fabricated, where the number of coil turns was sequentially increased with roughly twofold. The other circuit parameters of resistance and capacitance were modified according to the number of coil turns to tune the resonant frequency to be ~850 kHz (Fig. S13 and Tables S4,  S5). In order to evaluate the power transfer performance, the optimal coupling frequency and acceptable radiation distance between WPT receiver and transmitter were examined. During experiments, the WPT transmitter of the integrated antenna connected to a waveform generator and a network analyzer was aligned over the WTCL with an identical axis (Fig. 4a), while the WPT receivers were connected to an oscilloscope to monitor the generated voltages. The reflection coefficient spectra from four receivers at different radiation distances were recorded (Fig. 4b and Fig. S14), where the resonant frequency of the transmitter and all receivers were observed to be at ~850 kHz according to the circuit designs. The channel separation between IOP monitoring (~3.8 GHz) and WPT (~850 kHz) was sufficiently large to avoid cross-coupling, which might prevent unexpected activation of a nontargeted wireless channel in the IOP monitoring and administration^41^. The return loss (S11) revealed that most of the energy carried by electromagnetic waves could be radiated rather than dissipated in the frequency range of 837.38 to 867.45 kHz, with a bandwidth of transmitter of about 30 kHz^42^. The S21 under 850 kHz of all receivers decreased linearly with the increase of radiation distance (Fig. 4c). Considering that a certain distance between transmitting coils and contact lens is required to avoid interference to human eyes in practical applications, 6 mm was chosen as the optimal distance between transmitting coils and WTCL in experiments. Sequentially, a series of the square wave (20 Vpp) with different frequencies (500 to 1200 kHz, 50 kHz step) or at different distances (0 to 15 mm, 1 mm step) were wirelessly exerted on the transmitter, to further verify the optimized coupling frequency and distance. The generated sinusoidal voltages on the receivers were recorded by an oscilloscope (Fig. 4d, e and Fig. S15, S16), and the relations between peak to peak (Vpp) values and frequencies were analyzed. At the set distance of 6 mm, the Vpp increased sharply from 500 to 850 kHz and dramatically decreased from 850 kHz to 1.2 MHz, where the coupling at 850 kHz displayed a maximum Vpp of ~6 V (Fig. 4f), consisting of the previous results of resonant frequency at ~850 kHz. On the other hand, at the set frequency of 850 kHz, the Vpp of all receivers decreased with the increase of radiation distance (Fig. 4g). The insert loss (S21) and Vpp of Rec#17 were both significantly higher than those of other receivers at identical conditions, suggesting that Rec#17 possessed better matching with the WPT transmitter. Square wave (SquWave) and sine wave (SinWave), representing common voltage signals in analog electronics, possess distinct characteristics in rising and falling edges. In our experiments, SquWave and SinWave voltage signals featured with 20 Vpp and different frequencies (500 to 1200 kHz, with a step of 50 kHz) were exerted on the WPT transmitter (Fig. 4h and Figs. S17, S18) at different set distances (0 to 15 mm, 1 mm step) to the Rec#17, which was chosen as the optimized receiver design. The correspondingly collected Vpp of the receiver showed that the voltage-transfer behaviors at SinWave voltage were similar to that at SquWave, where 850 kHz was close to the optimal frequency. Moreover, the Vpp induced by the SquWave voltage wave was slightly higher than the that by SinWave (Fig. 4i, j), likely due to the fact that SquWave signals with more steeper edges created more rapidly changed magnetic field that is more favorable for WPT performance, compared to the SinWave at identical conditions.

To comprehensively evaluate the optimal conditions of WPT, receiver designs and the voltage-transfer conditions (the coupling frequency, the radiation distance, and the waveforms) were systematically analyzed (Figs. S15–S20) and summarized in two heatmap diagrams (Fig. 4k). Although the WPT efficiency was higher at a shorter radiation distance, 6 mm was selected as the optimal distance between transmitting coils and WTCL since the contact lens needed certain separation from the transmitting coils in practical applications. The maximum transferred Vpp was observed on the optimal receiver Rec#17 at the applied SquWave with a frequency of 850 kHz, which were identified as the optimal conditions for the final WTCL.

**Fig. 5:**
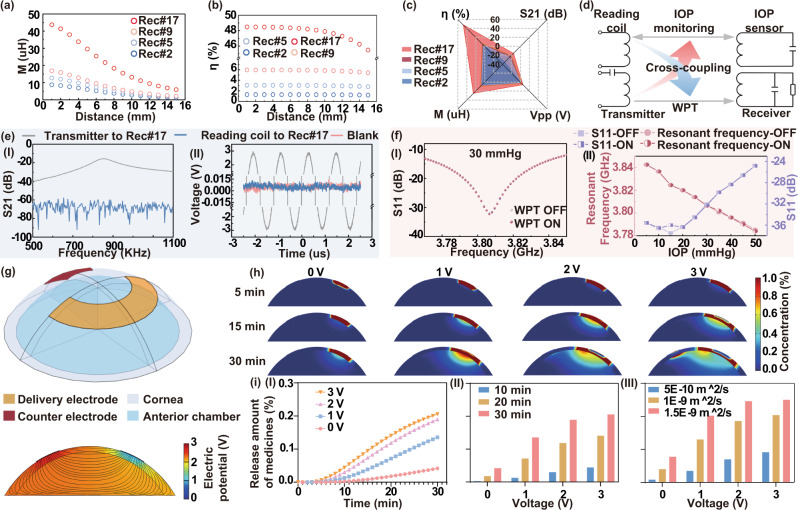
Avoidance of cross-coupling and drug delivery simulation. **a** The mutual inductance and **b** Power transfer efficiencies were theoretically calculated according to the circuit design of four receivers and the radiation distance. **c** Rader chart summarized performance (S21, Vpp, M, and η) of etch WPT group (Rec#2, Rec#5, Rec#9, or Rec#17 linked to transmitter with 6 mm radiation distance and 850 kHz). **d** Schematic showing the experiments studying the cross-coupling between IOP monitoring and WPT module. The red arrow denoted the interference generated by the radiation of the WPT transmitter to the sensing module. The Blue arrow denotes the cross-coupling between the IOP reading coil and the WPT receiver. All examinations were performed with the radiation distance of 6 mm. **e** The IOP reading coil and WPT transmitter were coupled with the WPT receiver (using Rec#17), respectively, and the S21 indicated the coupling efficiency and the generated voltages on the receiver radiated at 850 kHz were separately measured. **f** The WTCL was placed on the porcine eye at different IOP, and the reading signals (the resonance frequency and the S11) were recorded with or without the presence of radiation from the WPT transmitter. **g** The (top) 3D COMSOL model and (bottom) the simulated distribution profile of electric potential and electric field through the anterior region under the condition of iontophoresis at 3 V for *t* = 30 min. **h** The time slots of drug concentration profile delivered by WTCL at various applied voltages (0, 1, 2, and 3 V). **i** The delivered amounts of drugs under different conditions, including the (I) applied voltages, (II) iontophoretic duration, and (III) assumed diffusivities, were systematically evaluated.

The WPT performance of the WTCL was further theoretically analyzed, where the mutual inductance (M), power transfer efficiency (η) (Fig. S21), and the skin effects (Fig. S22) were calculated based on the circuit design. The Mutual inductance (M), a key factor in the technology of WPT, determines voltage in the coil of receiver circuits, were derived from the magnetic coupling coefficient according to the circuit design of four receivers^43^. The mutual inductance was inversely proportional to the radiation distance between transmitter and receiver circuits, where the Rec#17 group (transmitter and Rec#17) exhibited the highest value of mutual inductance than other groups (Fig. 5a). The power transfer efficiency of Rec#17 was further calculated to be 48.4% at the resonate frequency of 850 kHz and 6 mm radiation distance, significantly higher than the other three receiver designs (Fig. 5b), also consistent with the experimental results. Rader chart (Fig. 5c) visually summarized the performance (S21, Vpp, M, and η) of each WPT group (Rec#2, Rec#5, Rec#9 or Rec#17 linked to transmitter with 6 mm radiation distance and 850 kHz), where Rec#17 group showing greater chart area compared to other alternative groups. It demonstrated that Rec#17 group could serve as the optimal power transfer platform for further iontophoretic drug administration in this work.

### Evaluation of cross-coupling

The cross-coupling between multiple wireless channels is a significant concern since it may disturb the independent control over the in situ sensing and delivery modules (Fig. 5d). The conventional strategy to spatially avoid cross-coupling is less compatible with contact lens devices due to their limited space^44^. Here we employed a specialized technique of radiofrequency separation to solve the cross-coupling issue, based on a compact design of device to accommodate distinguished wireless circuits on the limited area of the contact lens. Firstly, the IOP reading coil and WPT transmitter were coupled with the WPT receiver (using Rec#17) at a set distance of 6 mm, respectively, and the S21 indicating the coupling efficiency and the generated voltages on the receiver were individually measured. The coupling between WPT transmitter and receiver exhibited S21 higher than −40 dB and reached its maximum value (−15.6 dB) at ~850 kHz (Fig. 5e-I), and an apparent sinusoidal voltage waveform with 6 Vpp (Fig. 5e-II) was recorded. The coupling between the reading coil and WPT receiver displayed ultra-low S21 (<−60 dB) (Fig. 5e-I) and negligible voltage generated that was close to a blank group of an uncoupled receiver (Fig. 5e-II), suggested cross-coupling between IOP reading coil and WPT receiver rarely occurred.

The WTCL was placed on the porcine eye at different IOP (0–50 mmHg), and the reading signals were recorded with or without the presence of radiation from the WPT transmitter (Fig. S23). The S11-frequency spectra appeared to be overlapping well disregarding the presence of WPT radiation, where a typical example at 30 mmHg IOP was shown in Fig. 5f-I. The resonance frequency and the peak S11 at different IOP were quantitatively analyzed (Fig. 5f-II), where the radiation of the WPT transmitter did not significantly influent the IOP monitoring, indicating cross-coupling between the IOP sensor and WPT transmitter was negligible.

### Ex vivo delivery of Rhodamine B into porcine eyes by WTCL

Brimonidine with positive charge is a medication that has been topically delivered to the aqueous humor or ciliary body to treat glaucoma clinically by increasing uveoscleral outflow and reducing aqueous fluid production^8,14^, as discussed in Fig. S24. However, the corneal barrier comprised of tightly packed epithelium and hydrophilic-hydrophobic interfaces could significantly hinder the passive diffusion of drug molecules from the ocular surface into the anterior chamber^39,45^. Iontophoresis that drives the migration of charged species via electric field have been successful in transdermal pharmaceutical delivery^46,47^, and the delivery dosage can be tuned by the iontophoretic strength or duration^48^. To achieve effective and controllable ocular drug delivery, iontophoresis is coupled to the WTCL to enhance the transport of bromonidine across the cornea layer in an on-demand manner. A mixture solution of HEMA monomers, crosslinker, photoinitiator, and brimonidine tartrate was drop-casted on the iontophoretic electrode surface and was irradiated with UVB light to form a brimonidine-loaded pHEMA hydrogel layer^49^. When wirelessly powered by the transmitter, the WPT receiver generated alternating voltages on the electrode, which electrically drove the brimonidine into the anterior chamber across corneal barriers^39^.

### Theoretical simulations of iontophoretic medicines administration

To understand the process of iontophoretic delivery, a simplified 3D model (top of Fig. 5g and Fig. S25) imitating the actual scenario of WTCL worn on an eye was established using COMSOL Multiphysics 5.5, where the electric currents interface and the transport of diluted species interface were employed to calculate the electrically-driven drug diffusion profile. The anterior was modeled as a cornea layer (consisting of the epithelial cell layer, stroma, and epithelial cell layer) covered on the anterior chamber, with corresponding electric conductivities and mass diffusivities. A drug delivery electrode coated with a thin layer of drug-loaded hydrogel and a counter electrode were conformally placed on the eye, and constant voltage was applied instead of alternating voltage in order to simplify the dynamic simulation process. Detailed boundary conditions and parameters were shown in Figs. S26,  S27 and Table S7 in supplementary materials. The distribution of electric potential and electric field through the anterior region under iontophoresis at 3 V at *t* = 30 min were shown at the bottom of Fig. 5g, and the time slots of drug concentration profile at various applied voltages (0, 1, 2, and 3 V) were shown in Fig. 5h and Fig. S28. Moreover, simulations for Rhodamine B delivery were also performed as shown in Fig. S29 and Table S8. Under iontophoresis, positively charged drug molecules migrated into the anterior chamber, where the drug delivery via iontophoresis was more effective (~3 to 5-folds higher) than the passive diffusion, due to the difficulty of drug diffusion across the cornea. The delivered amounts of drugs under different conditions, including the applied voltages, iontophoretic duration, and assumed diffusivities, were systematically evaluated (Fig. 5i). The results indicated the increase of these parameters would effectively enhance the drug delivery efficiency.

**Fig. 6:**
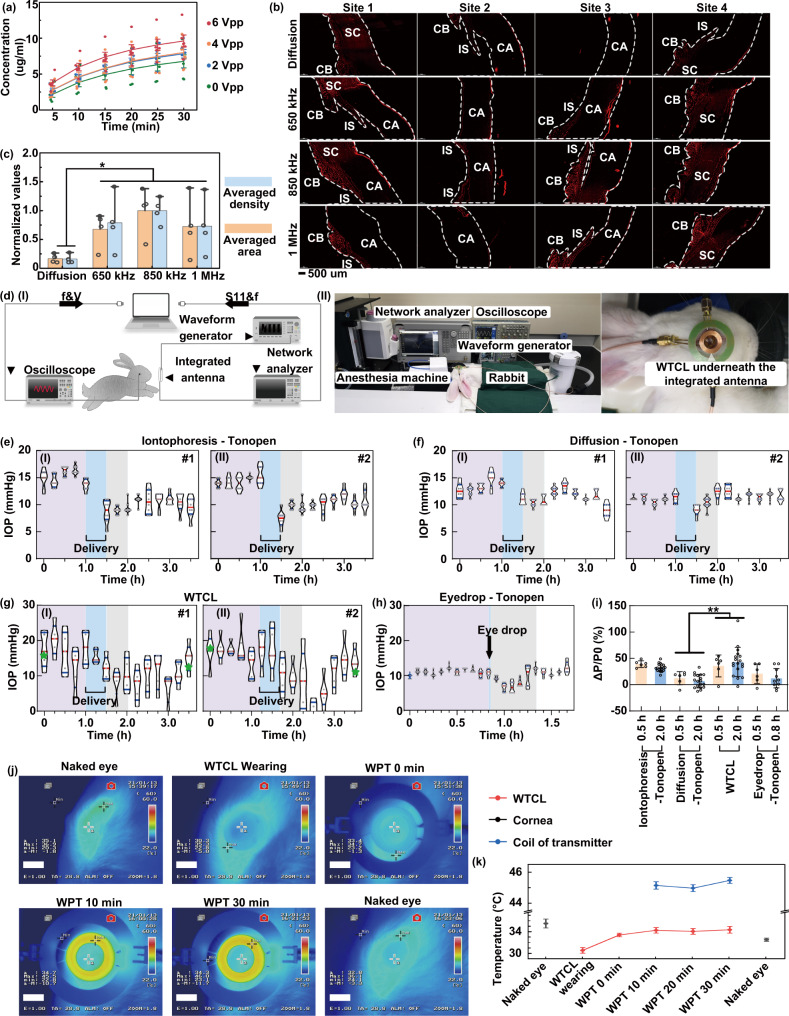
Sensing and therapeutic performance of the integrated WTCL. **a** Quantitative analysis of ex vivo rhodamine B released from WTCL at different alternating voltages for 30 min. *N* = 6 measurements at each time point. **b** Rhodamine B was utilized as the medicine analog in ex vivo experiments on porcine eyes, to examine the influence of iontophoresis on drug delivery across the cornea. CB ciliary body, IS iris, CA cornea. The scale bar is 500 µm. After delivery, fluorescence visualization in the anterior tissue was observed via microscope and (**c**) quantitatively analyzed. *N* = 4 sites per group. Significance was evaluated by one-way ANOVA analysis of variance, *p = 0.0351. **d** (I) Schematic and (II) experimental setup of the in vivo WTCL experiments. The rabbits were anesthetized and worn with WTCL on their eyes, while the signals recording of WTCL and WPT operation were conducted by the integrated antenna. The rabbits’ IOP were monitored with Tonopen, and brimonidine deliveries were performed via (**e**) Wirelessly powered iontophoresis or (**f**) passive free diffusion based on WTCL. *N* = 8 measurements at each time point. **g** Simultaneous IOP sensing and drug delivery using a single WTCL device. The rabbit’s IOP were wirelessly monitored with the WTCL, and brimonidine delivery via wireless iontophoresis on the same WTCL was conducted. The green asterisk indicated the IOP measurements via Tonopen for calibration or accuracy comparison. *N* = 5 measurements at each time point. **h** Rabbit’s eye was treated with eye drops of brimonidine, and the IOP was measured via Tonopen. *N* = 8 measurements per time point. *N* = 2 rabbits in (**e**–**g**) and *N* = 1 rabbit in (**h**). In (**e**–**h**), the purple, blue, gray, and white regions indicated the periods prior to delivery, during delivery, 0.5 h after delivery, and 2 h after delivery, respectively. **i** The IOP reductions effects after 0.5 and 2 h or 0.8 h of drug delivery via iontophoresis, free diffusion, and eye drops were summarized. Data were presented as mean ± SD. From left to right, *N* = 6, 18, 6, 18, 6, 18, 6, 10 data points (2 groups, each possessing 6–18 data points). Significance was evaluated by one-way ANOVA analysis of variance, *p = 0.0027. **j** Monitoring of the thermal effects generated during WPT operation via infrared thermal camera and (**k**) the temperate on the cornea, WTCL, and transmitter coils during WPT process were analyzed. *N* = 4 measurements per group. Data were presented as mean ± SD.

The ex vivo release of brimonidine (Mw 292.1) from pHEMA hydrogel-coated electrode surface at different iontophoretic voltages (alternating voltages with 0–6 Vpp) was performed using a red fluorescent dye, rhodamine B (Mw 479.0), as the medicines analog to facilitate quantifications via optical measurements. The results (Fig. 6a) showed that the dye molecule was continuously released at higher rates when higher voltages were applied, likely due to the fact that the electric field facilitated the diffusion of the dye out of the hydrogel layer. Ex vivo experiments on porcine eyes were performed to examine the influence of iontophoresis on delivery across the cornea, where rhodamine B was utilized as the medicine's analog to facilitate visualization of distribution in tissue. The WTCL was worn on porcine eyes, and voltages with 6 Vpp at 850 kHz (the determined optimal WPT operation frequency) was applied to facilitate dye delivery via iontophoresis, and the anterior tissue was then fixed and sectioned for fluorescence visualization via microscope. Iontophoresis at other frequencies (650 and 1 MHz) or passive free diffusion were tested to optimize the iontophoresis conditions, and the fluorescence intensity and distribution area in the anterior tissues were analyzed. Red fluorescence was clearly observed in the tissues of the ciliary body and anterior chamber angle for all the samples treated via iontophoresis (Fig. 6b), while the group of free diffusion exhibited significantly (>3-folds) lower fluorescence intensity and less (>3-folds) fluorescence distribution compared to the iontophoresis groups (Fig. 6c). Of note, the ciliary body and anterior chamber angle have been proven to be the target sites for suppressing IOP by brimonidine through reducing aqueous humor production and increasing uveoscleral outflow. These results suggested the coupled iontophoresis could facilitate the delivery of drug analog molecules into an anterior segment, and effectively work at the determined optimal WPT operation frequency of 850 kHz.

### In vivo experiments of WTCL performance

Next, in vivo experiments were conducted on rabbits, while the size of WTCL was proportionally scaled down to fit the rabbits’ eyes. The WTCL was worn on the anesthetized rabbits’ eyes, while the signals recording of WTCL and WPT operation were conducted by the integrated antenna (Fig. 6d). The rabbits’ IOP were monitored with either WTCL or commercial tonometry as a standard reference, and brimonidine delivery via wirelessly powered iontophoresis of WTCL was performed to reduce the IOP and compared to that via eyedrop. The initial IOP of rabbits exhibited slight fluctuation within the range of 10–15 mmHg as measured by Tonopen (Fig. 6e), which rapidly (<0.5 h) dropped by 39.2 ± 10.3% (Fig. S30) after brimonidine delivery via wirelessly powered iontophoresis (at 6 Vpp, 850 kHz, for 30 min), and the IOP reduction remained above 20% for the prolonged period (~2 h) after delivery (Fig. S31). In contrast, brimonidine delivery via free diffusion (for 30 min) from WTCL only slightly reduced IOP by 12.4 ± 14.3% within 0.5 h after delivery (Fig. 6f and Fig. S30), and produced negligible effects (6.85 + 14.7%) within 2 h (Fig. S32). These results suggested that the slow diffusion of brimonidine from WTCL might form a basal delivery to stabilize the IOP, while iontophoresis was able to facilitate a bolus delivery to more effectively reduce IOP spikes. Simultaneous IOP sensing and drug delivery using a single WTCL device were next performed (Fig. 6g). The rabbit’s IOP were wirelessly monitored with the WTCL for the first hour, then in situ, brimonidine delivery via wireless iontophoresis on the same WTCL was conducted to reduce IOP, which were still continuously monitored by the WTCL (Figs. S33,  S34). Considering the variations between ex vivo and vivo sensing and the differences between rabbit’ porcine’ eyes, the rabbits’ IOP were measured with Tonopen before experiments (at *t* = 0 h) to calibrate the WTCL’s sensing results (Figs. S35,  S36). The last data points of IOP recorded by WTCL were compared to the reference IOP measured via Tonopen after experiments, and the results showed that the sensing error of WTCL was <42%. The IOP was observed to gradually drop by 32.5 ± 35.9% (Fig. S30) within 0.5 h, and remained reduced by 43.2 ± 38.8% (Fig. S32) for a prolonged period. As a control, eye drops of brimonidine (1 mg/ml, 50 uL) were instilled into the rabbit’s eye, and the IOP measured via Tonopen showed a reduction of 30.9 ± 14.4% during a short period (<30 min) (Fig. S30), followed by rebounding rapidly to the initial IOP state. The IOP reductions effects via iontophoresis, free diffusion, and eye drops were summarized in Fig. 6e–i and Fig. S30–S32, and the results confirmed that the iontophoresis via WTCL rapidly reduce the IOP with pronounced and prolonged effects that were desirable for regulating IOP.

### Thermal characterization

In the end, since WPT operation at high frequency is likely to produce thermal effects that are harmful to animal eyes, the temperatures of rabbits’ eye surface (cornea) and WTCL were monitored via an infrared thermal camera during the process of WPT operation (Fig. 6j and Fig. S37). The temperature of the cornea was not increased, while the temperature of WTCL was observed to increase only by <3 °C (Fig. 6k), respectively, during WPT for 30 min, suggesting negligible thermal effects produced by WTCL.

## Discussion

In this work, a soft, minimally invasive, and battery-free WTCL system for in situ IOP tracking and on-demand medicines administration was developed. The delicate design for the structure and circuits layout of the device enabled integration on a limited area and curved surface without causing vision blockage as well as potential irritations. The compact lens was exploited as a platform for deploying wireless bioelectronics and intimate contact with the human cornea, while the fabrication is compatible with the high-throughput standard manufacturing process. The specialized design of frequency separation enabled individual operations of sensing and delivery modules without cross-coupling. Due to the unique cantilever configuration design of the LCR circuit, the embedded wireless IOP sensor could ultra-sensitively detect IOP fluctuation, while the drug delivery modulus coupled with iontophoresis enabled efficient release of drug permeating across the cornea. Systematic characterizations of IOP sensing, WPT, cross-coupling between individual sub-systems, iontophoretic medicines administration, and in vivo experiments all demonstrated the feasibility and promise of this WTCL platform for real-time monitoring and wireless controlled medical intervention. This smart system provides promising methodologies that could be expanded to other ophthalmic diseases, which would positively promote the emergence of a new generation of a theranostic system for personalized health management.

## Methods

### Theoretical analysis of WPT

Theoretical analysis of mutual inductance (M), power transfer efficiency (η), and the skin effects. Mutual inductance (*M*), a key factor in the technology of WPT, determines voltage in a secondary coil of receiver circuits. The critical parameter could be expressed as^50^:

1 $$M=k{({L}_{1}{L}_{2})}^{\frac{1}{2}}$$

where *L*_1_, *L*_2_ represent the inductance value of the coil integrated into the WPT transmitter and receiver circuit, respectively. *k*, denotes the magnetic coupling coefficient, which means the link of magnetic flux between the WPT transmitter and receiver side^43^. The parameter is approximately equal to the following equation when the radiation distance is comparable to coils dimension^43^.

2 $$k=\frac{1}{{[1+{2}^{\frac{2}{3}}{(\frac{d}{\surd {r}_{1}{r}_{2}})}^{2}]}^{\frac{3}{2}}}$$

where *d* refers to the distance between the WPT transmitter and receiver circuit. Furthermore, r_1_, r_2_ denote the radius of the inductance coil of the transmitter and receiver circuit.

According to these two equations mentioned above, the *M* was inversely proportional to the radiation distance for these two coaxial coils of the transmitter and receiver circuit.

According to Kirchhoff’s voltage law, the equation of the WPT system could be described as

3 $$\left[\begin{array}{c}{U}_{s}\\ 0\end{array}\right]=\left[\begin{array}{cc}{R}_{{PT}}+j\omega {L}_{{PT}}-j\frac{1}{\omega {C}_{{PT}}} & -j\omega M\\ -j\omega M & {R}_{{PR}}+j\omega {L}_{{PR}}+\frac{Z_{L}}{1-j\omega {C}_{{PR}}{Z}_{L}}\end{array}\right]\left[\begin{array}{c}{I}_{{PT}}\\ {I}_{{PR}}\end{array}\right]$$

Where *U*_*s*_, *R*_*PT*_, *L*_*PT*_, *C*_*PT*_, *I*_*PT*_ refer to the alternating voltage supplied for the transmitter, parasitic resistance, inductor, capacitor, and alternating current in the transmitter. Correspondingly, *R*_*PR*_, *L*_*PR*_, *C*_*PR*_, *R*_*L*_ denote the parasitic resistance, inductor, capacitor, and electric load in the receiver circuit. *I*_*PR*_ represents the total alternating current in the receiver circuit. *I*_*L*_ is the alternating current flow through the electric load.

To simplify the matrix, *Z*_PT_ and *Z*_PR_ were introduced as the impedance of the transmitter and receiver circuits and expressed as

4 $${Z}_{{PT}}={R}_{{PT}}+j{{{{{\rm{\omega }}}}}}{L}_{{PT}}-j\frac{1}{{{{{{\rm{\omega }}}}}}{C}_{{PT}}}$$

5 $${Z}_{{PT}}={R}_{{PT}}+j{{{{{\rm{\omega }}}}}}{L}_{{PR}}+\frac{{Z}_{L}}{1-{{{{{\rm{j}}}}}}{{{{{\rm{\omega }}}}}}{C}_{{PR}}{Z}_{L}}$$

Therefore, Eq. (3) could be transformed as

6 $$\left[\begin{array}{c}{I}_{{PT}}\\ {I}_{{PR}}\end{array}\right]=\frac{{U}_{S}}{{Z}_{{PT}}{Z}_{{PR}}+{\omega }^{2}{M}^{2}}\left[\begin{array}{c}{Z}_{{PR}}\\ j\omega M\end{array}\right]$$

And the current consumed by load is illustrated as

7 $${I}_{L}=\frac{j{{{{{\rm{\omega }}}}}}{{{{{\rm{M}}}}}}{U}_{S}}{(1-j{{{{{\rm{\omega }}}}}}{C}_{{PR}}{Z}_{L})({Z}_{{PT}}{Z}_{{PR}}+{{{{{{\rm{\omega }}}}}}}^{2}{M}^{2})}$$

The power transfer efficiency η was regarded to be the ratio of the real power dissipated in the load impedance ${P}_{{{{{{{\mathrm{out}}}}}}}}$ to the power supplied from the source side ${P}_{{{{{{{\mathrm{in}}}}}}}}$,

8 $${{{{{\rm{\eta }}}}}}=\frac{{P}_{{{{{{{\mathrm{out}}}}}}}}}{{P}_{{{{{{{\mathrm{in}}}}}}}}}=\frac{{{{{{{\rm{\omega }}}}}}}^{2}{M}^{2}{Z}_{L}}{{Z}_{{PR}}(1+{{{{{{\rm{\omega }}}}}}}^{2}{{C}_{{PR}}}^{2}{{Z}_{L}}^{2})({Z}_{{PT}}{Z}_{{PR}}+{{{{{{\rm{\omega }}}}}}}^{2}{M}^{2})}$$

As regards high-frequency circuits, alternating high-frequency currents tend to be distributed toward the surface of the conductor. This phenomenon, known as the skin effect, will increase the resistance of the conductor and reduce the effective electric power exerted on the load. The effective cross-section of the conductor for alternating currents was defined as skin depth that could be expressed by the following equation:

9 $$\delta ={({{{{{\rm{\pi }}}}}}{{{{{\rm{f}}}}}}{{{{{{\rm{\mu }}}}}}}_{{{{{{\rm{r}}}}}}}{{{{{{\rm{\mu }}}}}}}_{0}{{{{{\rm{\sigma }}}}}})}^{-\frac{1}{2}}$$

where *δ*, f, ${\mu }_{r}$, ${\mu }_{0}$, and σ represent skin depth in meters, frequency of the alternating current in Hz, the relative magnetic permeability of the conductive matter, the permeability of free space (4π × 10^−7^ H/m), and conductivity of conductor. Detailed parameters (relative magnetic permeability and conductivity of the conductive matter) were listed in Table S6.

### Fabrication of IOP monitoring circuits

The sensing and delivery were designed, and the fabricating process including Copper (Cu) film deposition, photolithography, etching, and laser cutting were performed via a standard flexible printed circuit fabrication process by Shenzhen Gaoyue Electronics Co. Ltd, China. Cu film deposition process (including sputtering and electrical plating) was performed to establish electric film on the surface of the PI substrate. After that, Cu film was patterned by photolithography and the development process. Extra Cu film was etched to form Cu electrodes, which were then covered with nickel (Ni) and gold (Au) to improve biocompatibility for the flexible circuits. Subsequently, an ultraviolet beam excited by a high-energy YAG laser was utilized to cut the PI substrate to form the snowflake-shaped layout for the flexible IOP sensing circuits.

### Fabrication of upper lens

Each capacitive sensing plate (total six plates) of the flexible IOP sensing circuit was aligned with the reference plate and folded manually. Then the folded IOP sensing circuit was positioned into the metal mold for the contact lens. Polydimethylsiloxane (PDMS, Sylgard 184, Dow Corning) and curing agent were prepared according to the ratio of 10:1 and then stirred sufficiently. The transparent PDMS solution was placed in a vacuum with a pressure of 10 Pa for 30 min to remove bubbles, and injected into the metal mold. After vacuum treatment (10 Pa, 30 min), the upper mold and bottom mold were assembled and placed in an oven (80 °C, 1.5 h). Afterward, the contact lens embedded with the IOP monitoring circuit was disassembled from the mold carefully. Finally, sensing plates were detached from the upper contact lens manually. While reference plates and five coils of inductance were kept inside the upper lens. The dangling sensing plates aligned with reference plates served as a cantilever configuration.

### Fabrication, soldering of drug delivery circuits

The fabricating process contained film deposition, electrical plating, chemical plating, photolithography, etching, drilling, and laser cutting of standard flexible printed circuit fabrication process (by Shenzhen Gaoyue Electronics Co. Ltd, China). Cu film deposition process (including chemical and electrical plating) was performed to establish electric film on the surface of the PI substrate. After that, Cu film was patterned by photolithography and development process and etched to form Cu electrodes, and the surface except the iontophoretic electrodes were insulated by a thin layer of PI. Ultraviolet laser with high energy was adopted to fabricate through-hole on PI substrate. Chemical and electrical plating were conducted to deposit Cu film on the surface of PI substrate and through-hole, which enables electric connections between electrodes on the top and bottom layers. Sequentially, photolithography, development, and wet etching process were performed to form patterned Cu electrodes that were then covered with Ni and Au layers to improve biocompatibility for the flexible circuits. High-energy laser was utilized to cut the PI substrate to define the flower-shaped layout for drug delivery circuits. Subsequently, ceramic chip capacitors (1 mm length, 0.5 mm width, 0.5 mm thickness) were attached to their respective sites on flexible circuits using low-temperature solder by an electrical soldering iron.

### Fabrication of bottom lens

The drug delivery circuit was positioned in a metal mold for a contact lens. PDMS solution was placed in a vacuum with a pressure of 10 Pa for 30 min to remove bubbles, and injected into the mold. After vacuum treatment (10 Pa, 30 min), the top and bottom molds were assembled and placed in an oven (80 °C, 1.5 h). Afterward, the PDMS contact lens integrated with the drug delivery circuit was disassembled from the mold carefully. Finally, extra PDMS film covered on iontophoretic electrodes (including delivery and counter electrodes) was removed manually.

### WTCL integration and drug loading

The upper lens integrated with the IOP monitoring circuit and the bottom lens embedded with the drug delivery circuit were assembled with liquid PDMS glue in the oven (60 °C, 3 h). The materials for preparing pHEMA hydrogel preparation included HEMA monomer (1.45 ml), EGDMA (5 μl) as a crosslinker, DI water (0.5 ml), and brimonidine tartrate (10 mg). Darocur (6 mg), a photoinitiator was mixed into a monomer mixture and sonicated. The mixture solution of pHEMA hydrogel (10 μl) loaded with brimonidine tartrate (5 mg/ml) was drop-casted onto the drug delivery electrode. The solution was irradiated with UVB light (365 nm) for 20 min for the hydrogel polymerization and kept at room temperature overnight. The surface of the delivery electrode was coated with a thin layer of pHEMA hydrogel with a thickness of ~50 μm, while the drug molecules were encapsulated in the hydrogel.

### Characterization of circuits

Microscopic images of IOP sensing and drug delivery circuits were captured by an inverted fluorescence microscope (MF52-N, Guangzhou Micro-shot Technology Co., Ltd, China)

### Ex vivo performance of wireless IOP monitoring

The porcine eyes were placed in the lab at room temperature (27 ± 3 °C) and humidity (50 ± 10%) to avoid the shape changes of eyeball induced by water loss. Before the deployment of the wearable smart contact lens, physiological saline solution (200 μl) was dript on the surface of the cornea to build a water film. The layer could be used to stimulate tear film to avoid bubbles between the cornea and contact lens. IOPs ranging from 5 to 50 mmHg were achieved inside the eye by injecting saline solution into the anterior chamber via a disposable intravenous infusion needle (0.45 × 13.5 mm) controlled by a syringe pump (PHD ULTRA, Harvard Apparatus, Inc., USA) During the experiments, a pressure gauge (GM511, Shenzhen Jumaoyuan Science And Technology Co., Ltd, China) was connected to the anterior chamber by a disposable intravenous infusion needle to independently track the value of IOP. The resonance frequency of the IOP monitoring module was recorded wirelessly by IOP reading coil (diameter: 17 mm, turns: 1) of the integrated antenna connected to a network analyzer (E5063A, Agilent Technologies Inc., Santa Clara, CA, USA).

### Dynamical response of wireless IOP monitoring

Physiological saline solution was injected into the anterior chamber of the porcine eye to elevate the IOP from 4.5 mmHg to 30 mmHg. Then, the pressure decreases down to 13 mmHg with the continuous leaking of solution from the eyeball. Sequentially, saline was filled into the ex vivo eyeball again to raise the pressure again. During this process, resonant frequency changes of the IOP monitoring module in WTCL were recorded wirelessly by a network analyzer, and the pressure data in the anterior chamber was validated using a commercial pressure gauge via a disposable intravenous infusion needle.

### WPT performance characterization of the WTCL system

During the measurements, the integrated antenna was immobilized above WTCL deployed on the porcine eye. The ex vivo organ that was posited on cystosepiment board was held by a multi-axis stage to adjust the distance between WTCL and the integrated antenna. During the collection of scattering parameter, two ports of the vector network analyzer (E5063A, Keysight Technologies, USA) was connected to the WPT transmitter of the integrated antenna. Four different receivers (Rec#2, Rec#5, Rec#9, and Rec#17 integrated into four WTCLs) were connected to the network analyzer successively to conduct data collections. During the measurements of wireless voltage-transfer performance, a waveform generator (DG1022, Beijing RIGOL Technology Co., Ltd., China) was adopted as a power source for operating the WPT transmitter of the integrated antenna. The oscilloscope (TDS2014C, Tektronix, USA) was connected to the WPT receiver circuit of WTCL for recording the voltage signal collected wirelessly.

### Cross-coupling characterization

(1) Disturbance generated by the radiation of WPT transmitter to IOP sensing module. The saline solution were injected into the anterior chamber of ex vivo porcine eyes via infusion needles (0.45 × 13.5 mm) controlled by a syringe pump to achieve IOPs ranging from 5 to 50 mmHg. The network analyzer was connected to the IOP reading coil of the integrated antenna to monitor the physiological pressure transduced by WTCL. The distance between the integrated antenna and WTCL was set as 6 mm. A commercial pressure gauge was exploited to validate the shifts of pressure inside of the anterior chamber through an infusion needle (0.45 × 13.5 mm). Moreover, the waveform generator was connected to the WPT transmitter of the integrated antenna. During the process of IOP sensing, the power of the waveform generator to support the WPT transmitter was turned on and off to observe and record the S11 response of the IOP monitoring module. (2) Characterization of WPT performance of Rec#17 integrated into WTCL under the radiation of IOP reading coil. During the measurements, the integrated antenna was immobilized above WTCL deployed on the porcine eye. The distance between the integrated antenna and WTCL was set as 6 mm. The IOP reading coil and Rec#17 integrated in WTCL was connected to two ports of the network analyzer for the recording of the S21 parameters. As a control group, the IOP reading coil was replaced by a WPT transmitter that was coupled with Rec#17 to record S21 parameters. Similarly, The IOP reading coil of the integrated antenna was connected to the waveform generator. While the Rec#17 circuit integrated in WTCL was connected to an oscilloscope for the collecting of voltage signals. Correspondingly, a WPT transmitter was adopted to replace the IOP sensing coil. Moreover, the integrated antenna (including the IOP reading coil and WPT transmitter) was disconnected from the waveform generator, while Rec#17 was still connected with an oscilloscope. The collected data serve as a blank group.

### Theoretical simulations of iontophoretic medicines administration

The theoretical simulations of iontophoretic medicines administration were performed with COMSOL Multiphysics software using the AC/DC module and Chemical Species Transport module. To visualize the effect of active agents administration and spatial distributions of electric potential, the process of cargo delivery were simulated with a 3D model, where the components and geometric layouts mimicked the actual experimental setup. Anterior segment of the eye was modeled as aqueous humor, cornea including epithelial cell, stroma, and endothelial cell layer. The counter electrode and pHEMA hydrogel that served as reservoirs to load bio-active compounds were attached to the cornea. Furthermore, the back surface of hydrogel was set as a drug delivery electrode to generate electric potential. Correspondingly, the counter electrode was labeled with the ground in an electric field. Under the action of constant electrical voltages, the working electrode combined with the counter electrode forms an electric field through the tissue of the cornea. For drug delivery, the drug concentration was set as Cg0 in hydrogel and gradually diffused into aqueous humor through corneal barriers facilitated by an electric field. After that, the average compounds concentration in the anterior chamber (aqueous humor) was calculated to evaluate the drug delivery efficiency. The bio-active molecules' concentration was then normalized by comparing it to the initial cargo’s concentration loaded in a hydrogel. Critical factors in this simulation work involve: (1) the drug diffusivities and the electrical conductivities in the pHEMA hydrogel, corneal layers, and aqueous humor; (2) Electrical charge of drugs. The detailed physic setting of cargo administrations, and related parameters were demonstrated in table S7 in this supporting information file.

AC/DC module was exploited to simulate the steady electric field distribution, which was performed by electric currents interface, following the theoretical equation:

10 $$\nabla {{\cdot }}J=0$$

11 $$J=\sigma E$$

12 $$E=-\nabla V$$

Where V denotes potential, E refers to the intensity of the electric field, J represents current density, σ is the material conductivity, $\nabla$ refers to Hamiltonian. These equations mentioned above contributed to a Laplace equation that could be adopted to calculate electric potential and electric field in this model:

13 $$\nabla \left(\sigma {{\cdot }}\nabla V\right)=0$$

On the top boundary of the drug delivery electrode, a boundary voltage terminal was used to simulate the constant voltage source:

14 $$V={V}_{0}$$

Where ${V}_{0}$ refers to constant voltage.

Then, the dynamic iontophoresis process was simulated by chemical species transport module according to the electric field distribution. The theoretical equation could be expressed as:

15 $$\frac{\partial c}{\partial t}+\nabla \cdot {J}_{{tds}}=0$$

16 $${J}_{{tds}}=-{D}_{e}{\nabla }_{C}-z{u}_{{me}}{Fc}\nabla V$$

Where ${J}_{{tds}}$ refers to diffusion flux vector, c denotes the concentration, z represents the charge number, F refers to Faraday constant, V is electric potential, ${D}_{e}$ corresponding to the effective diffusion coefficient, ${u}_{{me}}$ denotes the effective mobility. Therefore, the relationship of ${D}_{e}$ and ${u}_{{me}}$ can be demonstrated by Nernst–Einstein equation:

17 $${u}_{{me}}=\frac{D{{{{{\rm{e}}}}}}}{{RT}}$$

Where R represents Moore gas constant and T is temperature.

### Quantitative analysis of rhodamine B released from WTCL

In this work, rhodamine B was exploited as the medicines analog to visualize the distribution of medicines in bio-tissue. Rhodamine B in PBS solution with a concentration of 0.5 to 30 ug/ml was prepared, and the standard curve of absorption value and solution’s concentration was established. Then HEMA monomer (1.45 ml), EGDMA (5 μl) as a crosslinker, DI water (0.5 ml), and photoinitiator Darocur (9 mg) were mixed. The mixture solution of pHEMA hydrogel (10 μl) loaded with rhodamine B (0.4 mg/ml) was drop-casted onto the drug delivery electrode of WTCL. After that, the solution coated on WTCL was irradiated with UVB light (365 nm) for 20 min for the hydrogel polymerization and kept at room temperature overnight. The WTCL is connected to an oscilloscope, and powered by the WPT transmitter of the integrated antenna underneath the WTCL (distance: 6 mm). About 200 μl PBS solution was placed on the WTCL to allow dye diffusion, and 100 μl solution was withdrawn every 5 min for analysis of the diffusion rate. Accordingly, fresh PBS with equal volume was re-supplemented into WTCL. These absorbance values of solutions collected at each time point was measured by a microplate reader. According to these absorbance values, the accumulated concentration of released rhodamine B could be calculated according to the equation mentioned above.

### Ex vivo delivery of rhodamine B into porcine eyes by WTCL

The WTCL was worn on an ex vivo porcine eye purchased from a country market in Guangzhou Higher Education Mega Center, and the integrated antenna was deployed on top of the WTCL at a distance of 6 mm. The waveform generator was connected to the WPT transmitter of an integrated antenna as the power source. An oscilloscope was connected to the WPT Rec#17 circuit in WTCL to monitor the voltage adapted for iontophoresis and ensure that the received alternating voltage stabilized around 6 Vpp with the frequency of 650, 850 kHz, and 1 MHz, respectively. Moreover, drug delivery in the manner of free diffusion was performed as a control group. A through-hole (diameter: 4 mm) was created in the central area of WTCL to allow the dropping of PBS solution to the eye surface for maintaining humidity. During experiments, PBS was instilled into the central hole of WTCL at the speed of 30 µL every 30 s to form a thin solution film on the corneal surface. The liquid film has been regarded as a simulant of tears to ensure a reliable connection between drug delivery and cornea electrically, and also prevent drying of ocular surface tissue^39^. After the completion of examinations, the corneal surface of the porcine eye was irrigated by PBS. Then extra tissues (muscle, fat) outside of the eyeball were removed by dissecting scissors. The whole eyeball was fixed in a paraformaldehyde solution (Fixative Solution, 4% formaldehyde, methanol-free, Biosharp Co., Ltd, China), sectioned, stained by Wuhan Servicebio Technology Co., Ltd. Sequentially, fluorescent microscopic images including bio-tissue of the ciliary body, iris, the cornea (site 1–4) in each experiment condition (free diffusion, and 6 Vpp with the frequency of 650, 850 kHz, 1 MHz) were taken and processed by Image J program to quantify the fluorescence intensity and distribution area in the anterior tissues. The mean distribution area and the integrated density of rhodamine B in the sample treated by iontophoretic drug administration with 20 Vpp at 850 kHz were set to be a base reference of 1 for normalization. Correspondingly, normalized values of distribution area and the integrated density of rhodamine B in the sample treated by other drug delivery conditions could be quantified.

### Animal experiments

Female New Zealand white rabbits (4–5 months old) weighing about 2 kg (Animal Center, Sun Yat-sen University, Guangzhou, China), adopted for in vivo experiments, were maintained in a climate-controlled independent room with 12 h/12 h light/dark cycle separately. Eight rabbits were used for in vivo WTCL data collection. All in vivo experiments in this study were reviewed, permitted, and supervised by the Institutional Animal Care and Use Committee of the Sun Yat-sen University (SYSU-IACUC-2020-B0071, SYSU-IACUC-2021-000509) and by Animal Experimental Ethics Committee of Zhongshan Ophthalmic Center at Sun Yat-sen University (No. 2020-004). For all in vivo experiment processes, the rabbits were deeply anesthetized with pentobarbital sodium solution (0.8 ml/kg body weight). To minimize side effects, the administration of anesthetic solution was separated into three times through ear venous and twice intramuscular injections every ten minutes successively. Moreover, Isoflurane and oxygen were supplied through a gas anesthesia machine for rabbits to obtain prolonged anesthesia effects. Propivacaine hydrochloride eye drops (S. A. ALCON-COUVREUR N.V. Belgium) were dropped onto the rabbit cornea surface for topical anesthesia to avoid ocular movement including blinking, facilitate WTCL wearing, and IOP measurement by commercial ophthalmotonometer.

### In vivo experiments of WTCL performance

Pentobarbital sodium (Nembutal, Ovation Pharmaceuticals Inc. Deerfield, USA) solution in saline (0.3 wt%) was prepared. New Zealand white rabbits were initially anesthetized with an appropriate dose of pentobarbital sodium solution (0.8 ml/kg body weight), and continuously anesthetized with an Isoflurane anesthesia machine. To avoid unexpected situations, the administration of anesthetic solution was divided into three times through ear venous and twice intramuscular injections every ten minutes successively. After anesthesia, the rabbits were covered with a blanket to maintain body temperature. Propivacaine hydrochloride eye drops were dropped onto the rabbit cornea surface for local anesthesia to further avoid ocular movement including blinking. A commercial applanation tonometer (TonoPen Avia; Reichert, Inc., Depew, NY) was applied to acquire IOP measurement as a reference. WTCL was worn on a rabbit’s eye, and an oscilloscope was connected to the WTCL to monitor the Vpp between delivery and counter electrode during the WPT process. An integrated antenna connected to a network analyzer and waveform generator was posited above WTCL with a distance of 6 mm. Sequentially, measurements of return loss by the IOP reading coil was collected by a network analyzer to wirelessly detect IOP. After 1 h, square voltage with 20 Vpp at 850 kHz produced from the waveform generator was exerted on the WPT transmitter to trigger iontophoretic delivery wirelessly. Meanwhile, wireless IOP monitoring was continuously performed until the end of the experiments.

### Thermal characterization

The rabbit was anesthetized with an appropriate dose of pentobarbital sodium solution (0.8 ml/kg body weight) through ear venous injections. After general anesthesia, the rabbits were covered with a blanket to maintain body temperature. Then anesthesia machine was further adopted to supply isoflurane and oxygen via facemask for the rabbit, which could obtain prolonged anesthesia effects. WTCL was worn on a rabbit’s eye, and an integrated antenna connected to a waveform generator was posited above WTCL at a distance of 6 mm. Square voltage with 20 Vpp at 850 kHz produced from the waveform generator was exerted on the WPT transmitter. An infrared camera (T650sc, FLIR Systems, Wilsonville, OR, USA) was exploited to monitor thermal changes of ocular surface tissue, WTCL, and integrated antenna during the experimental process.

### Statistics and reproducibility

One-way analysis of variance (ANOVA) among multiple groups was performed for statistics. All the data were presented as the mean ± SD. *P* values were calculated by PRISM software (GraphPad). No animals were excluded from the analysis.

### Reporting summary

Further information on research design is available in the Nature Research Reporting Summary linked to this article.

## Supplementary information

Supplementary Information

Reporting Summary

## Data Availability

All relevant data supporting the key findings of this study are available within the article and its Supplementary Information files or from the corresponding author upon reasonable request. Source data are provided with this paper.

## Source data

Source Data
