# Supplementary material for: Intelligent wireless theranostic contact lens for electrical sensing and regulation of intraocular pressure
Source: Nat Commun. 2022 May 17;13:2556. doi: 10.1038/s41467-022-29860-x (PMC9114010; doi:10.1038/s41467-022-29860-x)
Supplement: Supplementary file 1 — Supplementary Information [file 41467_2022_29860_MOESM1_ESM.pdf]

Supplementary Information

Intelligent wireless theranostic contact lens for electrical sensing and regulation of intraocular pressure

Cheng Yang[1], Qianni Wu[2], Junqing Liu[4], Jingshan Mo[1], Xiangling Li[1,5], Chengduan Yang[1,3], Ziqi Liu[1], Jingbo Yang[1,5], Lelun Jiang[5], Weirong Chen[2], Hui-jiuan Chen[1], Ji Wang[3], Xi Xie[1,3]*

1. State Key Laboratory of Optoelectronic Materials and Technologies, Guangdong Province Key Laboratory of Display Material and Technology, School of Electronics and Information Technology, Sun Yat-Sen University, Guangzhou, 510006, China
2. State Key Laboratory of Ophthalmology, Zhongshan Ophthalmic Center, Sun Yat-Sen University, Guangzhou, 510006, China
3. The First Affiliated Hospital of Sun Yat-Sen University, Sun Yat-Sen University, Guangzhou, 510006, China
4. Department of Cardiology, the First Affiliated Hospital of Jinan University, Guangzhou 510630, China
5. School of Biomedical Engineering, Sun Yat-Sen University, Guangzhou, 510006, China

*Corresponding to email: xiexi27@mail.sysu.edu.cn

List of Contents:

S1. Discussion on the potential clinical applications of WTCL.

S2. Advances of WTCL

S3. The fabrication and structure of the WTCL.

S4. Supporting Information for the performance characterization of the IOP sensing module.

S5. Supporting Information for the design and performance characterization of the WPT module of the WTCL.

S6. Theoretical calculations for the performance of WPT groups.

S7. Characterization of cross-coupling between IOP monitoring and WPT activated iontophoresis of the WTCL.

S8. Theoretical simulations of iontophoretic medicines administration via COMSOL Multiphysics 5.5.

S9. Supporting information for in vivo experiments.

S10. Thermal analysis

S1. Discussion on the potential clinical applications of WTCL:

1) **WTCL for the acute angel-close glaucoma before surgery:** for acute angle-closure glaucoma featured with sudden rise of IOP, it is usually accompanied by headache, nausea and vomiting that hinders manually self-administrations by patients, while the delayed reduction of IOP will inevitably causes ischemic infarcts and damage optic nerve[1]. The WTCL may provide a new theranostic strategy for patients who suffer from acute angle-closure patients during the emergent occurrence of high IOP, especially for those patients lack accompanies or nursing in-house. This would reduces the risks of vision damages caused by sharply increased IOP and endows precious time for patients to appeal further medical intervention. Although the patients may not like to wear the WTCL for long term, this device can be used for acute angle-closure glaucoma patients before surgery. In clinical cases, many acute angle-closure glaucoma patients need to be treated with medicines to reduce IOP until it is stable and suitable for surgery, and this pre-surgery period generally takes ~1 week. This scenario would be easier for patients to accept the use of WTCL since a surgery has been scheduled. The WTCL can be applied on this pre-surgery period, which could continuously monitor the IOP states and prevent emergent occurrence of high IOP.

2) **WTCL used as contact lens bandage for glaucoma combined diseases after surgery:** the WTCL is essentially a smart ophthalmic bandage, which could be applied in clinical scenario that bandage contact lens needs to be used after ophthalmic surgery. For example, many patients experience combined cataract-glaucoma, where cataract surgeries are performed to treat the acute angle-closure glaucoma. Owing to release of proteins liberated by manipulation of intraocular structures during surgery, obstruction of trabecular outflow by inflammatory and viscoelastic material may occur and induce transient high IOP spikes after cataract operation[2]. The postoperative IOP rise can triggers ocular pain, corneal oedema, glaucomatous nerve damage or anterior ischaemic optic neuropathy, if not treated in time[3]. For example, in one clinical case (July, 2021) at Zhongshan Ophthalmic Hospital of Sun Yat-sun University, a patient (male, ~60-years-old) possessed glaucoma combined with cataract was treated with cataract surgery. During the night right after the cataract surgery, the nurse found his IOP suddenly increased to ~47 mmHg. After emergent treatments to reduce his IOP, his IOP was found to be ~45 mmHg on the next day, where the doctors were unclear when the IOP occurred again. These patients would benefit if the high IOP states could be monitored and regulated timely. Since many patients are suggested to wear bandage contact lens after cataract surgery to protect the wound for couple of days, the replacement of conventional contact lens with smart contact lens IOP sensor after surgery would be easier to be accepted for patients. Similarly, after glaucoma laser surgery, there are risks that the blood or tissue residues after surgery may cause obstruction of lachrymal duct that induced emergent case of high IOP. The WTCL can be applied on this post-surgery period to reduce the IOP risks.

3) **WTCL as drug release platform for ophthalmic diseases related to high IOP:** contact lens for continuous drug releases have been widely reported[4], which possesses

clinical significance in that the contact lens platform could enhance the bioavailability of ophthalmic drugs for the therapies on chronic ocular diseases. For example, controlled-release systems of ophthalmic drugs for the treatment of uveitis are desirable to reduce side effects of pharmaceutical agents, while uveitis and the treatment can induce elevated IOP clinically. The combined functions of contact lens-based sustained drug delivery and intelligent IOP sensors of WTCL would find some clinical utilities if the ophthalmic disease is associated with high IOP.

4) **WTCL as scientific tools for in vivo animal experiments:** the WTCL could also serve as scientific tools for in vivo animal experiments, where the wearing of contact lens on animals during the experimental period can be ensured. For the biomedical researchers study how disease progress or therapeutic techniques induce IOP fluctuation[5], this WTCL platform could provide continuous IOP monitoring on animals.

S2. Advances of WTCL

Challenges of integrated wireless contact lens for IOP monitor and regulation: Although the contact lens sensing and delivery technologies have been independently developed by other investigators, the integration of these two wireless functionalities possesses critical challenge on the device structure, rather than a straightforward process of "sensing" plus "delivery". The key challenge is how to achieve wireless channel separation to avoid cross-coupling between two wireless functionalities on the curved contact lens with limited surface, while the electrode surface area needs to be sufficiently large in order to maintain sensor sensitivity and delivery efficiency. The key merits of our WTCL include the proposed highly sensitive cantilever sensor that allows integration of delivery coils/electrode that compete with sensor surface and even partially block the sensor. We explain these in detail below and compare our device to commercial IOP contact lens sensor or other groups' sensors to clarify the critical challenges.

First, in order to achieve dual- or multi-functions on a single contact lens device, the wireless channels need to be sufficiently separated. For conventional devices with sufficient size, the cross-coupling can be avoided by spatially separation of different channels. However, for contact lens or implantable devices with ultra-small surface area, spatial separation is rarely possible. Instead, we employed the strategy of frequency separation for our WTCL. The wireless coupling frequencies for sensing and delivery need to be sufficiently separated, for example, one in the range of 1 MHz and the other one in the range or 100 MHz or even GHz. Otherwise, the wireless radiation for sensing could undesirably trigger non-target drug delivery if the frequency separation is insufficiently large.

According to the wireless radiation equation $f=\left(2\pi\sqrt{LC}\right)^{-1}$, lower coupling frequency requires larger number of coils, larger coil diameter, or larger capacitance. In order to accommodate operations at MHz for delivery and other one for sensing at higher frequency, the total coils area of these two coils on a single circuit plane would

exceed the surface area of contact lens (as we showed in Supporting Information S5.1, where we designed several coils parameters during device optimization). Many reported contact lens IOP sensors are very unlikely to directly add a second coils next to the first coils on the same circuit plane for wireless drug delivery (**Figure S1a**).

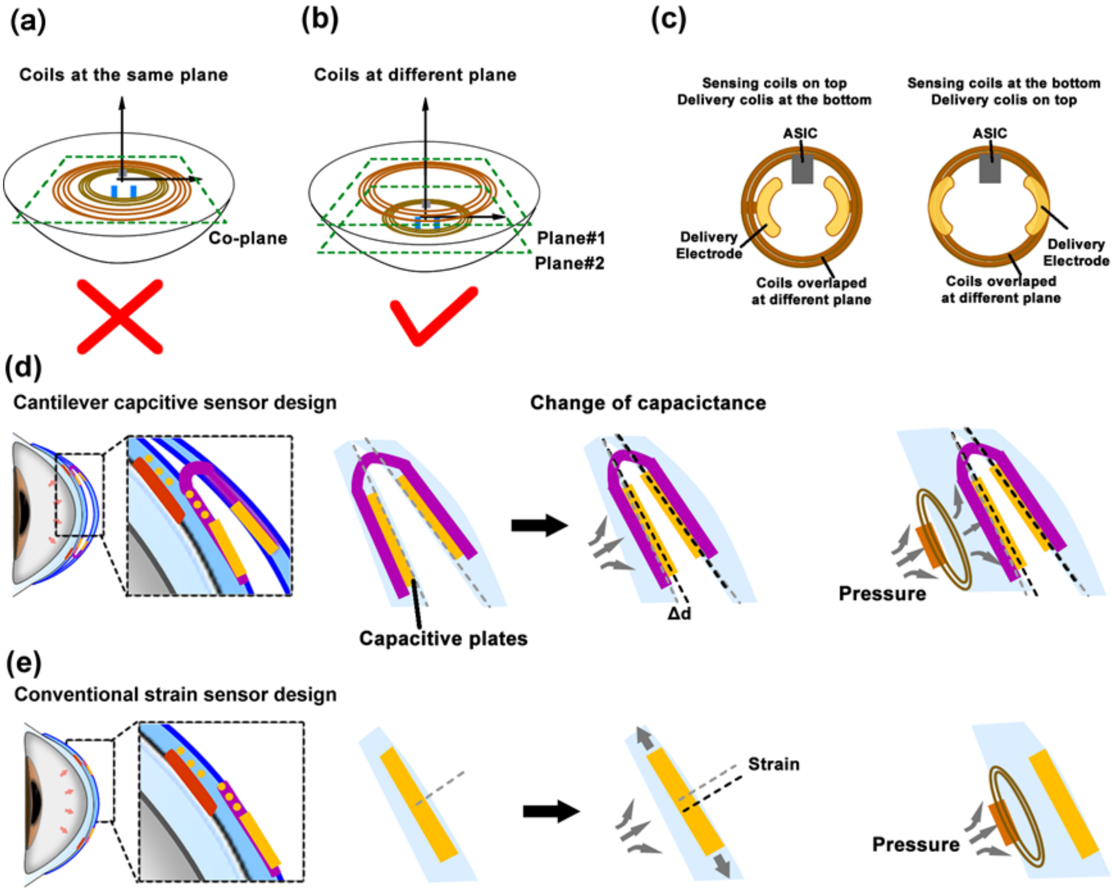

Supplementary Figure S1. (a) Schematic showing the design with both wireless drug delivery and wireless sensing circuits directly deployed on the same circuit planes. (b) Schematic showing the design with wireless drug delivery and wireless sensing circuits deployed on different circuit planes. (c) Left: Schematic showing the designs of delivery coils underneath the sensing coils. Right: Schematic showing the designs of delivery coils on top of the sensing coils. (d) Schematic showing the sensing mechanism of the cantilever capacitive sensor-based contact lens, where the capacitive plates were sensitively displaced upon pressing. (e) Schematic showing the sensing mechanism of strain sensor-based contact lens, where the sensor measured the resistance change of strain gauge embedded into the periphery of a soft silicone contact lens upon pressing.

Second, in order to ensure that IOP sensing module is able to detect mechanical signals sensitively, while drug delivery module can achieve wireless power supply stably, we proposed a multi-layers circuit design for our WTCL (**Figure S1b**). However, this multi-layers circuit design is also challenging to employ for many other contact lens sensors, because the lower layer of delivery coils would easily block the upper layer of sensors. Some commercial sensors measured the circumferential

changes of the eyeball at the corneoscleral interface by an active strain gauge embedded into the periphery of a soft silicone contact lens. The output is an equivalent of the electric voltage (mV) that are transmitted to the recorder via an integrated ASIC chip and coils. This strain gauge is less compatible with a secondary layer of delivery coils with electrodes. The drug delivery electrodes need to be in direct contact with the corneal surface, while the sensor is also more favorable to be close to the cornea. If the delivery coils are underneath the sensing coils, the delivery electrodes need to extrude from the coils to be in close contact with the eye surface (**Figure S1c-left**). In this case, both delivery electrodes and sensor chip would occupy a large amount of space of eye surface, and the rigidity of the electronics near the eye centers would cause more uncomfortable feeling. On the other hand, if the sensing coils are underneath the delivery coils (**Figure S1c-right**), the delivery electrodes could be overlapping with the coils positions to be in close contact with the eye surface. However, the delivery coils blocking the underneath sensor could greatly reduce the sensitivity especially for strain sensor, since the strain could be significantly buffered by the delivery coils (**Figure S1d** and **S1e**).

Limits of established contact lens devices for IOP monitoring and drug release: Although optical methods for IOP monitoring could also be wireless, the optical methods are less likely to be developed as continuous monitoring sensor for contact lens because the requirements of bulky recording modules of spectrometer or camera placed on the face. Camera is also difficult to be close to the eye as wearable recording system due to the requirement of certain focal distance. Moreover, the spectrometer or camera need to be directly aligned to the eyes, which will block the visions. Therefore, the spectrometer or camera are unsuitable to be worn as continuous recorders. The optical methods are usually less compatible with patients' motion since they can be easily interfered by the environmental lightness. The sensing sensitivity of optical methods are often lower than electrical methods, requiring large change of eyeball curvature to induce readable signals. The accuracy is also highly susceptible to the environments.

Additional challenge of the device continuing for practical applications: The thickness, curvature, diameter, edge design, diameter of the optical area, and oxygen transmission rate of the contact lens materials are important factors for the practical clinical application of contact lens-based bioelectronics. In this work, the circuits are integrated with PDMS contact lens by a cast-molding technique, where the contact lens substrate can be flexibly replaced with other polymer materials using similar integration technique. While the key advances of the WTCL are solving the critical challenges on highly sensitive IOP, wireless frequency separation and wireless-regulated iontophoresis, the substrate materials of PDMS could be further optimized to increase biocompatibility when this device is moving toward clinical tests.

S3. The fabrication and structure of the WTCL.

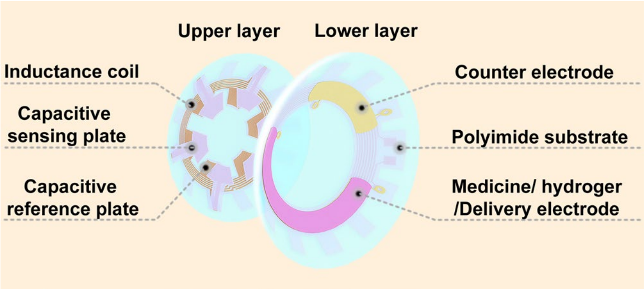

Supplementary Figure S2. Structure of the WTCL. The reference plates and 5 coils of inductance were embedded in the upper lens, while dangling sensing plates that aligned with reference plate contacted the front surface of the lower lens, with a dielectric air film between the reference and sensing plates forming a variable capacitor. Drug delivery circuit was embedded in the lower lens, while the drugs-coated iontophoretic electrodes on the bottom side of delivery circuit were exposed and would be in contact with the cornea.

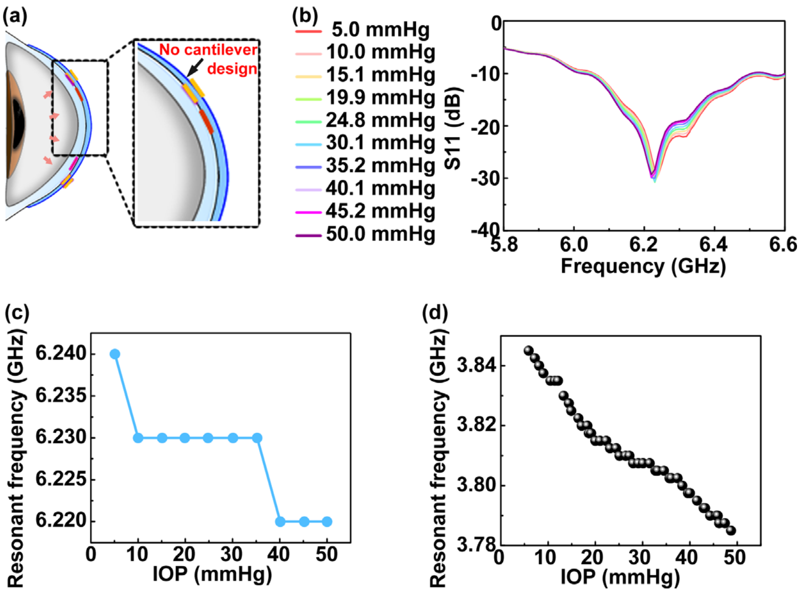

Supplementary Figure S3. Data showing that the sensitivity of non-cantilever-based capacitive pressure sensor embedded with PDMS between capacitive plates is significantly lower. (a) Schematic IOP sensing performance of the WTCL without cantilever design. (b)The reflection spectra and (c) Resonant frequency versus IOP value of contact lens capacitive sensor without cantilever-based design. The measurements were performed on porcine eyeball at different IOP. (d) Resonant frequency versus IOP value of WTCL devices with cantilever-based sensor design

(Device-I from Figure 3d of Manuscript), for comparison to (c). These results shown that cantilever design enables linear and sensitive IOP responsion.

Table S1. Detailed parameters and size of WTCL

**Contact lens**	Contact lens diameter (mm)	14.15
	Base curve of lower contact lens (mm)	8.6
	Base curve of upper contact lens (mm)	8.24
	Thickness of each contact lens (μm)	120
**IOP Monitoring circuits**	Outer diameter of IOP sensing circuit d_1 (μm)	10300
	Inner diameter of IOP sensing circuit d_2 (μm)	7900
	Inner diameter of capacitor d_3 (μm)	5000
	Branch length of IOP sensing circuit d_4 (μm)	2566
**WPT circuits**	Branch width of WPT circuit d_5 (μm)	1222
	Branch length of WPT circuit d_6 (μm)	986
	Outer diameter of WPT circuit d_7 (μm)	10800
	Inner diameter of WPT circuit d_8 (μm)	7450
	Space of the inductance electrode (μm)	70
	Width of the inductance electrode (μm)	80
	Central angle of drug carrier's electrode θ (deg)	180
	Central angle of counter electrode α (deg)	50

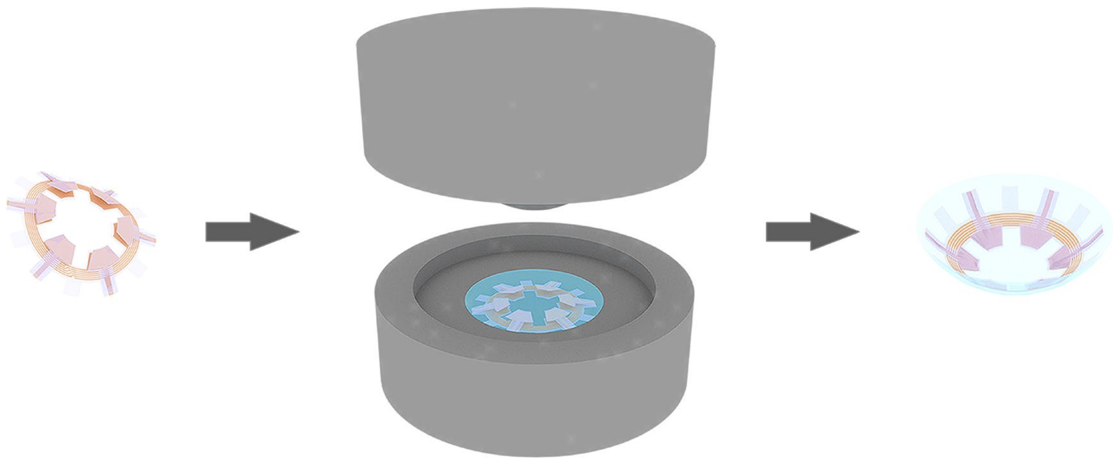

Supplementary Figure S4. (a) Schematic illustration of the cast-molding technique.

Cast-molding technique: the flexible IOP sensing circuit was folded, and positioned into the metal mold of contact lens. Polydimethylsiloxane (PDMS, Sylgard 184, Dow Corning) and curing agent were mixed according to the ration of 10:1 and then stirred sufficiently. The PDMS solution was placed in vacuum with pressure of 10 Pa for 30 min to remove bubbles, and then injected into the mold. After vacuum treatment (10 Pa, 30 min), the bottom mold and top mold were assembled and placed into oven (80 °C, 1.5 h). Finally, the contact lens embedded with circuit was detached from mold carefully.

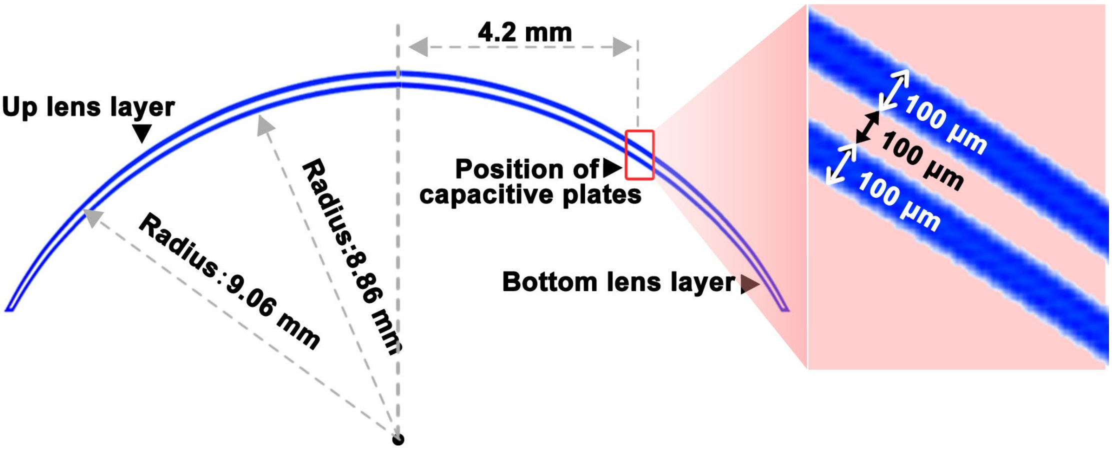

Supplementary Figure S5. (a) Schematic illustration of the WTCL's double contact lens structure.

Basic curves of Up and bottom lens were 9.06 mm, and 8.86 mm. And their thicknesses were 100 μm.

S4. Supporting Information for the performance characterization of the IOP sensing module.

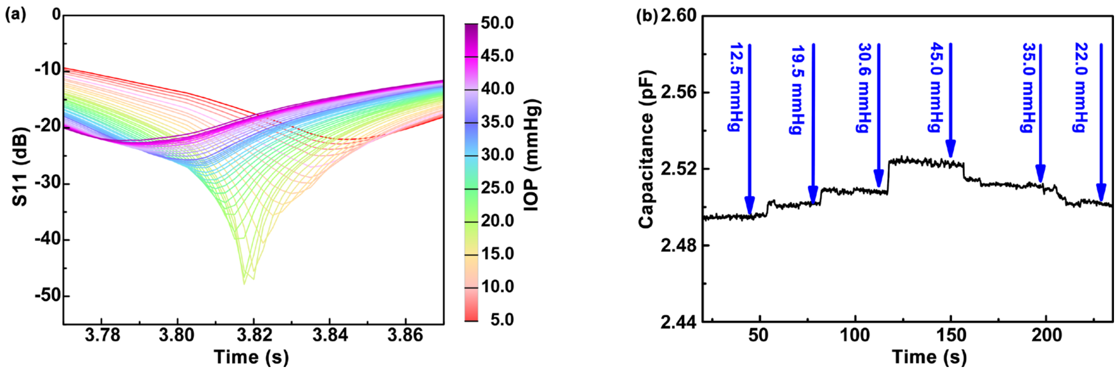

Supplementary Figure S6. (a) The detailed reflection spectra of device-I following

IOP ranging from 5 to 55 mmHg with the step of 1 mmHg. The static sensing performance was conducted by stepwise increase of IOP, which was controlled by microinfusion pump that drove saline solution into the anterior chamber and monitored by a pressure gauge. Meanwhile, the resonance frequencies of WTCL worn on the porcine eyeball at different IOP (5-50 mmHg) were recorded by Network analyzer connected to IOP reading coil (diameter: 17 mm, turns: 1) of the integrated antenna that was positioned above WTCL with nearly 6 mm-distance, where the resonant frequency of IOP monitoring module was found to shift to the lower frequency at higher IOP. (b) Capacitance change of the pressure sensing module as a function of time when the IOP was changed. During experiments, the WTCL was worn on porcine eyeball, and the IOP in porcine eye was controlled by the volume of infused saline solution with microinfusion pump. Meanwhile, a pressure gauge was adopted to track the IOP value. The capacitance formed by reference, sensing plates, and air film was connected with LCR meter (Changzhou Tonghui Electronic Co. Ltd., China) to recorded the value changes. Theoretically the resonant frequency is in inversely related to the capacitance according to the LCR circuit equation $f=\left(2\pi\sqrt{LC}\right)^{-1}$. The results were consistent with the theoretical prediction in that the increase of IOP would reduce the distance between the reference and sensing plates, hence led to the elevation of capacitance value.

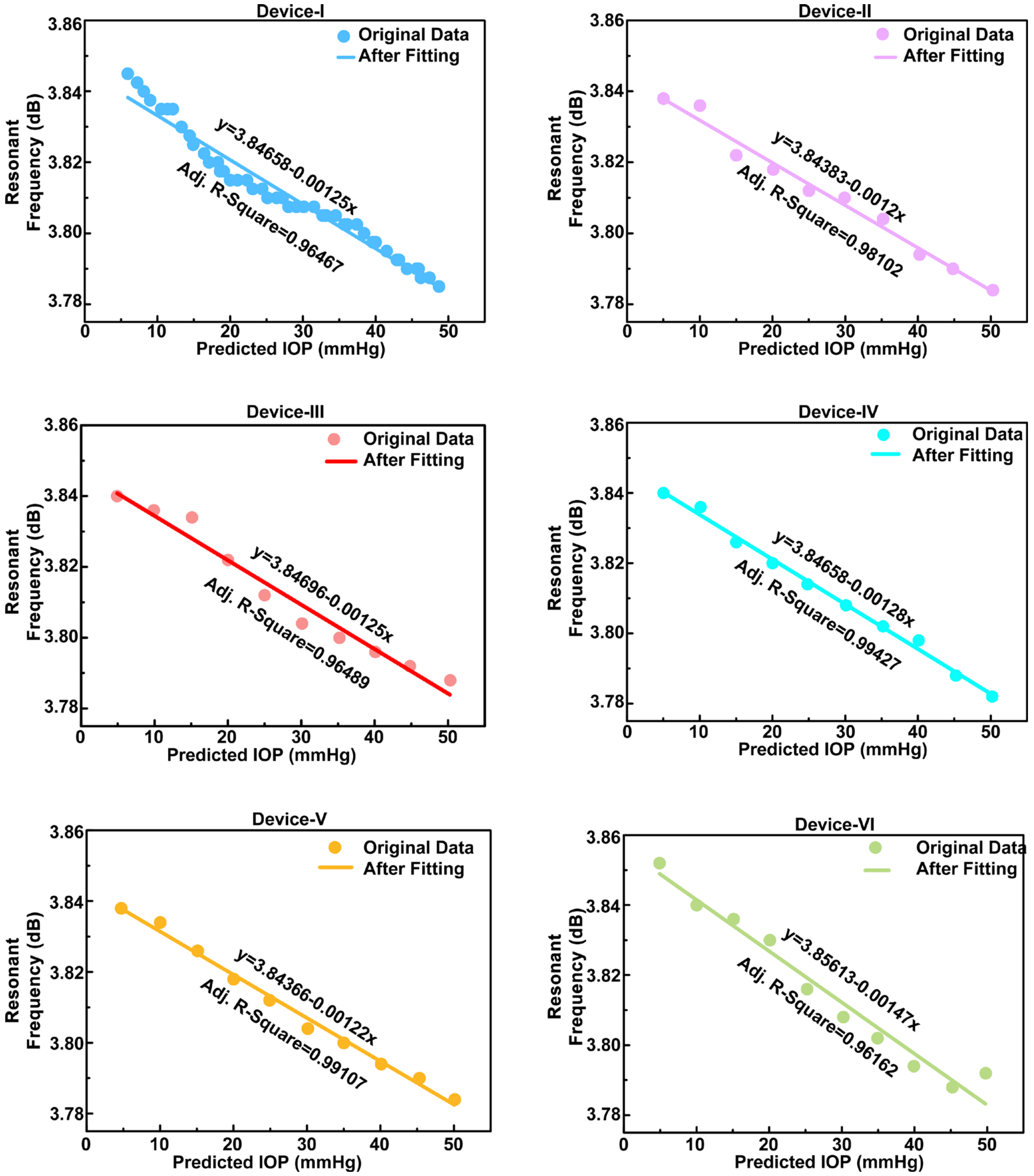

Supplementary Figure S7. The relation of resonant frequency versus IOP value of six representative WTCL devices worn on the porcine eyeball at different IOP (5-50 mmHg). The values of resonant frequency were collected from reflection spectra of each representative WTCL devices. The IOP values were validated by commercial gauge. The relations of resonant frequency versus IOP value were processed by linear fitting. These results demonstrated that signal responses of these sensors were linearly correlated to the reference IOP.

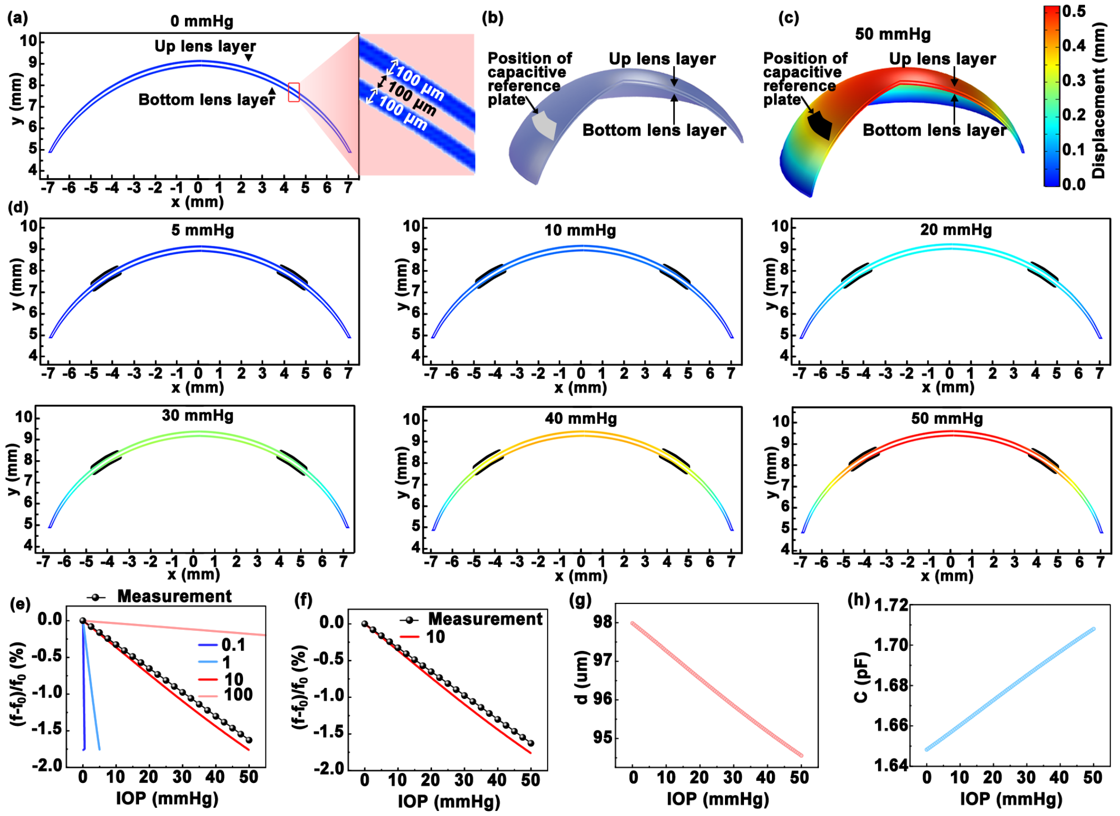

Supplementary Figure S8. Theoretical simulations of wireless IOP responsion via COMSOL Multiphysics 5.5. (a) Two-dimensional cross section view of double layer contact lens. The thickness of upper, lower lens layer and air dielectric layer were labeled in the insert figure. (b) Three-dimensional double layer contact lens model. Up, lower lens layer and position of capacitive reference plate were marked. (c) Deformation of double layer contact lens at the condition of 50 mmHg IOP. (d) Deformation of double layer contact lens with the IOP elevated from 5 mmHg to 50 mmHg. (e) Wireless IOP responsion with correction factors (δ) from 0.1, 1, 10 to 100. Black dots analyzed from Figure 3e was served as counter group. (f) Contrast between measurement result (black dots) and simulation data after correction with δ=10. (g) The distance between the upper and lower capacitor plates with the IOP elevated from 5 mmHg to 50 mmHg after correction treatment (δ=10). (h) the value of capacitance variation with the IOP elevated from 5 mmHg to 50 mmHg after correction treatment (δ=10).

Table S2. Detailed parameters of COMSOL simulations for contact lens deformation

Components	Symbol	Value	Definition

Top lens layer	H_t	100 μm	The thickness of top lens layer.
Top lens layer	$C_{t\text{-}B}$	9.06 mm	The basic curve of top lens layer.
Bottom lens layer	H_b	100 μm	The thickness of bottom lens layer.
Bottom lens layer	$C_{b\text{-}B}$	8.86 mm	The basic curve of bottom lens layer.
Contact lens	E	750 kPa	Young's modulus of top and bottom lens layer
Capacitance	C_x	4.2 mm	The position of capacitance from symmetrical axis of contact lens.
Capacitance	S	3.04 mm^2	The area of capacitance electrode.
Capacitance	C_N	6	The numbers of capacitance.
Capacitance	ε_0	8.85×10^{-12} F/m	The permittivity of vacuum.
Capacitance	ε_r	1	The relative dielectric constant of air.
Capacitance	d	Variable	The distance between pairs of capacitance electrode.
Inductance	d_{in}	8.6 mm	The diameter of inner coil.
Inductance	d_{out}	9.8 mm	The diameter of out coil.
Inductance	w_1	80 μm	The width of coil electrode.
Inductance	w_2	70 μm	The gap width of coil electrode.
Inductance	L_N	5	The turns of inductance coils.
IOP	P	Variable	

The theoretical simulations of contact lens deformation were performed with COMSOL Multiphysics software using the Solid Mechanics module. To visualize the strain effect of contact featured with double lens layer, the deformation of contact lens was simulated with a 3D model, where the components and geometric layouts mimicked the actual experimental setup.

We used COMSOL to build a 3D model of the double-layer contact lens structure as shown in **Figure S8a** and **Figure S8b**. The results shown that deformation of both the upper and lower contact lens occurred when the IOP varies in the range of 5 mmHg-50 mmHg (**Figure S8c** and **Figure S8d**), where the position of the capacitor plate in contact lens shifted. Correspondingly, the distance between the upper and lower capacitor plates at different IOP could induces capacitance fluctuations:

$$C = \frac{\varepsilon_r \varepsilon_0 S C_N}{d}$$

Where ε_r denotes relative dielectric constant of air, ε_0 refers to permittivity of vacuum, S represents area of capacitance electrode, C_N is the numbers of capacitance, d refers

to distance between pairs of capacitance electrode.

The inductance of the sensing module of WTCL can be calculated by.:

$$L=\left(\ln\left(\frac{2.46\left(d_{out}+d_{in}\right)}{d_{out}-d_{in}+2W_1}\right)+0.2\left(\frac{d_{out}-d_{in}+2W_1}{d_{out}+d_{in}}\right)^2\right)\frac{4\pi\times10^{-7}L_N{}^2\left(d_{out}+d_{in}\right)}{4}$$

Where d_{out} denotes diameter of out coil, d_{in} refers to diameter of inner coil, W_1 represents width of coil electrode, L_N is the turns of inductance.

Based on the calculated capacitance and inductance, the resonant frequency of the LC oscillation circuit in the sensing module of WTCL can be calculated by:

$$f=\frac{1}{2\pi\sqrt{LC}}$$

The normalized resonant frequencies of the LC oscillation circuits at different IOP can thus be calculated. However, the actual WTCL device is composited of flexible circuit with a polyimide (PI) substrate embedded in the PDMS contact lens. These polyimides, as well as the metal electrodes, cause changes in the Young's modulus of the double layer PDMS contact lens. We used the experimental results of resonant frequency-IOP response measured in main text **Figure 3e** (black dots in **Figure S8e**) to correct the theoretical model. Taking these material effects into account, a series of correction factors (δ = 0.1, 1, 10, 100) were used to correct the COMSOL model. It shown that the result with a correction factor δ=10 (blue curve) is closer to the actual experimental results (purple curve) as demonstrated in **Figure S8f**. Based on this correction factor δ=10, the inverse proportional relationship between IOP and distance between capacitor plates were shown in **Figure S8g**, and the relation between the capacitance an individual capacitor plate and the IOP were also presented in **Figure S8h**.

Table S3. Summary of the performance of wearable and implantable IOP sensors reported in the literature.

Wearable / Implantable	Sensing mechanism	Dielectric material	Response characteristics	Ref

Wearable	Capacitance	Ecoflex	About 2.2 MHz/mmHg	[6]
	Capacitance	Air	23 kHz/mmHg	[7]
	Inductor	Phthalocyanine	8 kHz/mmHg	[8]
	Inductor	Parylene C	57 kHz/mmHg	[9]
	Inductor	Parylene C	35.1 kHz/mmHg	[10]
	Capacitance	Air	1.25 MHz/mmHg	This work
Implantable	Capacitance		243 kHz/mmHg	[11]
	Capacitance		1.14 MHz/mmHg	[12]
	Capacitance		156 kHz/mmHg	[13]

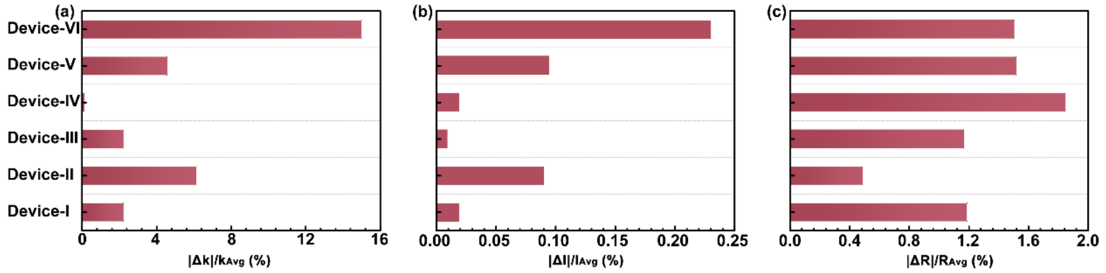

Supplementary Figure S9. Assessment of the reproducibility of the six IOP sensors, where the (a) Normalized slope, (b) normalized intercept, and (c) normalized R-Square of each sensor were quantified. Normalized slope was defined as $\Delta k/k_{Avg} = (k - k_{Avg}) \times 100\%/k_{Avg}$, where k represents linear fitting slope of IOP response from each sensor, k_{Avg} denotes the average value of all slopes. Normalized intercept was defined as $\Delta I/I_{Avg} = (I - I_{Avg}) \times 100\%/I_{Avg}$, where I represents linear fitting intercept of IOP response from each sensor, I_{Avg} denotes average value of all intercept. Normalized R-Square was defined as $\Delta R/R_{Avg} = (R - R_{Avg}) \times 100\%/R_{Avg}$, where R represents linear fitting R-Square of IOP response from each sensor, R_{Avg}

denotes average value of all R-Square. These results showed that the linear relations of all the six devices overlapped well (variations of the normalized slope, intercept, and R-Square were <15%, <0.25%, and <2%, respectively) with each other, indicating the reliability and repeatability of the fabricated device using our design.

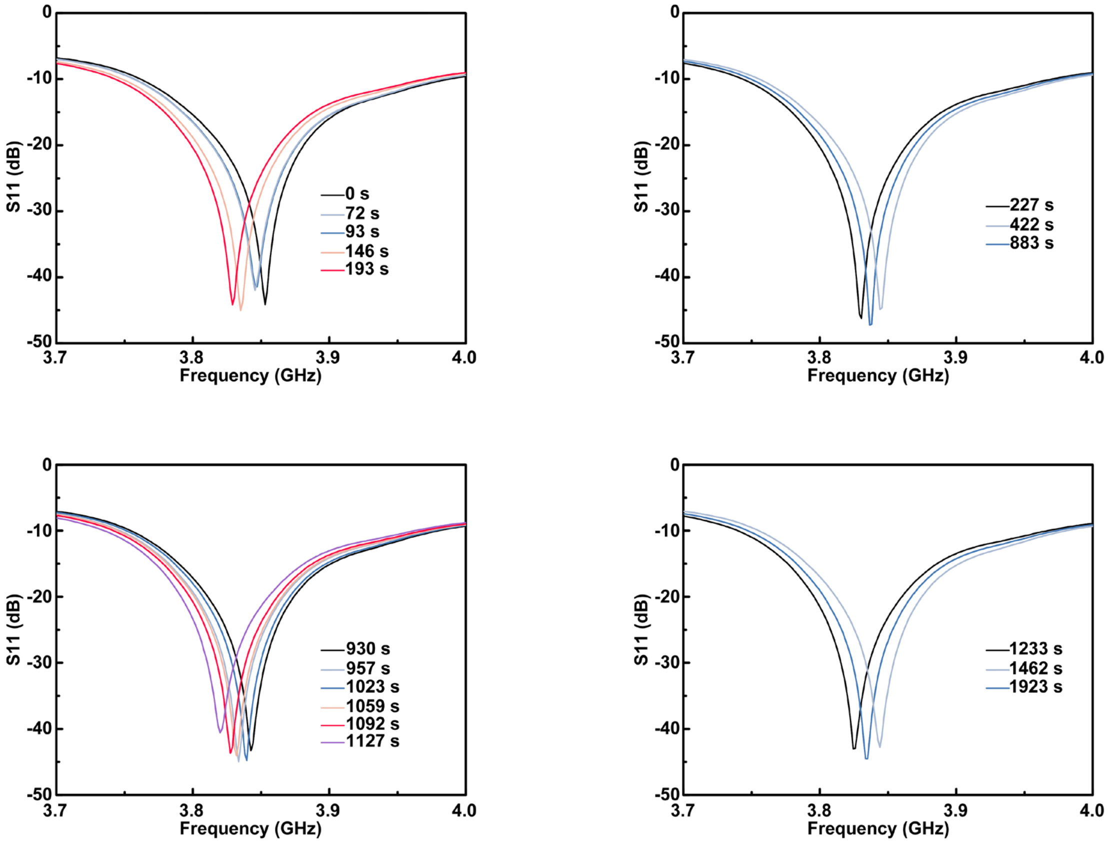

Supplementary Figure S10. The detailed reflection spectra recorded during the continuous monitoring of IOP via WTCL. During the experiment process, saline solution was injected into the anterior chamber of porcine eye to elevate the IOP from 4.5 mmHg to 30 mmHg. Then, the pressure decreases down to 13 mmHg with the continuous leaking of solution from eyeball. Sequentially, saline was filled into eyeball to raise the pressure again. The corresponding reflection spectra in these four phases involving IOP elevation and decreasing collected wirelessly.

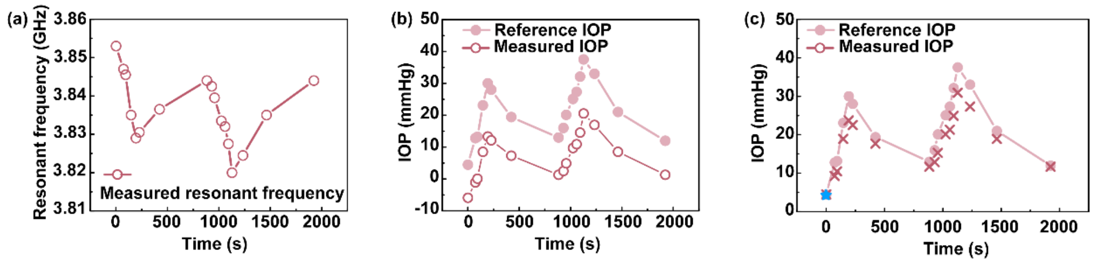

Supplementary Figure S11. Illustration and analysis of the wireless IOP sensing experiment on the porcine eyeball during the continuous IOP monitoring process. (a) Wireless recorded resonant frequency of IOP sensing module during the continuous IOP monitoring. In the experiment process, saline solution was intermittently injected into the anterior chamber of porcine eye to induce IOP fluctuations. Meanwhile, IOP reading coil of integrated antenna could recorded the changes of resonant frequency wirelessly by WTCL worn on the ex vivo eye. In addition, commercial pressure gauge was connected to the anterior chamber by a disposable intravenous infusion needle to independently track the reference IOP. (b) Reference IOP recorded by pressure gauge and measured IOP calculated from resonant frequency during the measurement process without calibration. (c) Measured IOP after calibration. Reference IOP was also shown as a control group. In the calibration process, the measured IOP calculated from resonant frequency was calibrated to the reference IOP (indicated with blue asterisk). Specifically, the measured IOP were proportionally scaled up according to the difference between the first measured IOP (at time points t=0 s) and the first reference IOP.

Discussion of the sensor calibration to address signal shifting in practical applications: In practice, before each wearing of the WTCL, patients could make one measurement of IOP using the conventional tonopen and then calibrate the standard curve of the WTCL to overcome the signal shift due to the difference of wearing. As the results of IOP continuous assays we showed in Figure 3h and Figure 6g, we both measured the reference IOP using tonopen at the starting time point of the IOP monitor, and then wore the WTCL on eye for sensing, whose signals were calibrated

with the reference IOP measured by tonopen. On the other hand, one more potential calibration method is using the positive and negative electrodes of the drug delivery module as additional calibration electrodes. We can measure the change of impedance between the positive and negative electrodes of the delivery module, which could reflect the change of contact between the WTCL and cornea. The sensing signal of WTCL could be compensated for the signal shift caused by change of contact. According to the literature, changes in edema, endothelial and epithelial permeability would be characterized by noninvasive measurements of corneal impedance[14,15]. The cornea can be equated to an impedance, which is connected to a wireless drug delivery circuit to form a composite impedance. The state of corneal and electrode attachment can be inferred by testing the composite impedance and the equivalent impedance of the powered coil by a wireless method, and the acquired corneal physiological parameters can then be fed back to the IOP test data for calibration. This method allows the sensor to reach a good accuracy. Sensor calibration is also commonly applied in many continuous physiological monitoring systems. For example, continuous glucose monitors (CGM) also often use fingerstick to measure blood glucose every day to calibrate the signal of CGM.

S5. Supporting Information for the design and performance characterization of the WPT module of the WTCL.

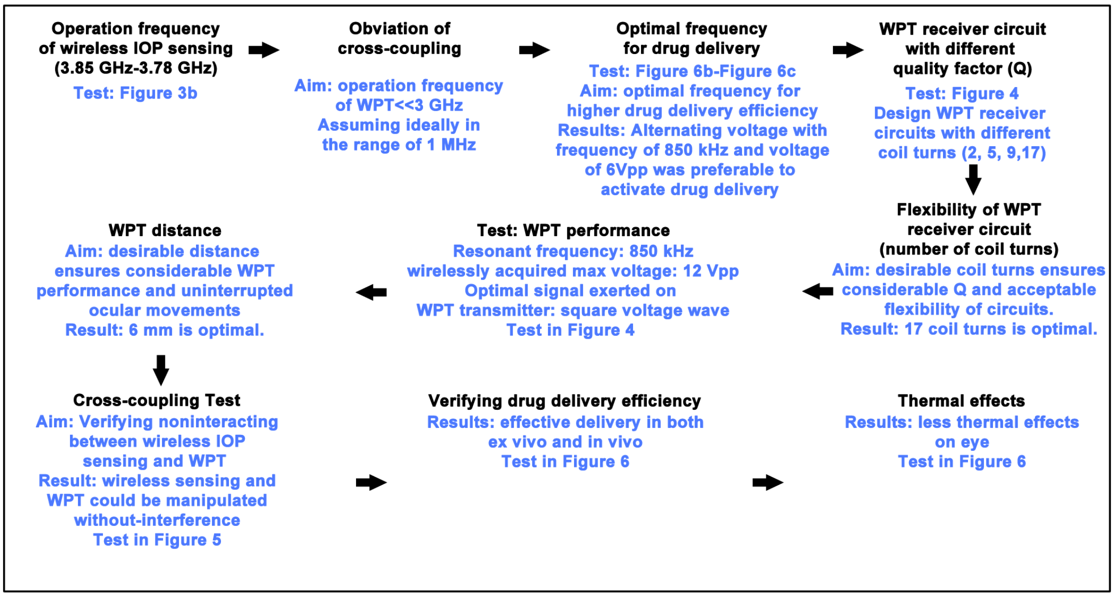

Supplementary Figure S12. Illustration of the WPT device design and optimization process.

To improve the efficiency of wireless power supply, more turns of receiving coils are not necessarily improve the efficiency. This is because increasing the number of coil turns will lead to higher resistance and parasitic capacitance of the inductor coils. This will cause the increase of impedance of the receiving coil, while the wirelessly transmitted power will be excessively consumed by the high-impedance receiving coil. In this work, two wireless modules (IOP sensing and WPT driven drug delivery) need to be manipulated synergistically. In order to avoid cross-coupling between the two wireless modules, the operating frequency of the wireless power supply module and the wireless IOP sensing module need to be sufficiently separated in frequency band. In order to achieve effective frequency separation, the operating frequency of the wireless power supply module is designed to be much lower than the operating frequency of the wireless IOP sensing module (3.85 GHz-3.78 GHz). In this work, we set the operating frequency of the wireless powered drug delivery module in the range of 1 MHz, which is well separated from the operating frequency of wireless IOP sensing (3.8 GHz). Secondly, we investigated the of the iontophoretic efficiency effect activated by alternating voltage-mode with different frequencies (0.65-1.2 MHz range) to drive the fluorescent drug rhodamine B into the anterior chamber tissue of the eye. We found that alternating voltage-mode was able to facilitate the diffusion of molecules through the cornea into the aqueous humor than free diffusion. In our experiments, we found that alternating voltage (850 kHz, 6Vpp) possessed an optimal iontophoresis-promoted drug delivery effects (Figure 6b and 6c). Therefore, we used 850 kHz as the operating frequency of the wireless power supply module. Improving the quality factor of the winding is an effective way to improve the efficiency of wireless power supply. To achieve the wireless operating frequency of the delivery module in the 850 kHz range, we further design the receiving circuit resonated at 850 kHz with different coil number to optimize the quality factor. We increased the number of coils by a factor of nearly 2-folds, where 2,5,9,17 turns were designed,

respectively. Increasing number of coils would lead to thicker circuits, reducing device flexibility. Our studies finally determined that using a coil with 17 turns of inductance allowed for a higher quality factor in the receiver circuit, as shown in the following table. With our current device architecture, a 17-turn coil design possessed the optimal performance. Too many coils would result in thicker circuit, compromising the device flexibility for practical applications.

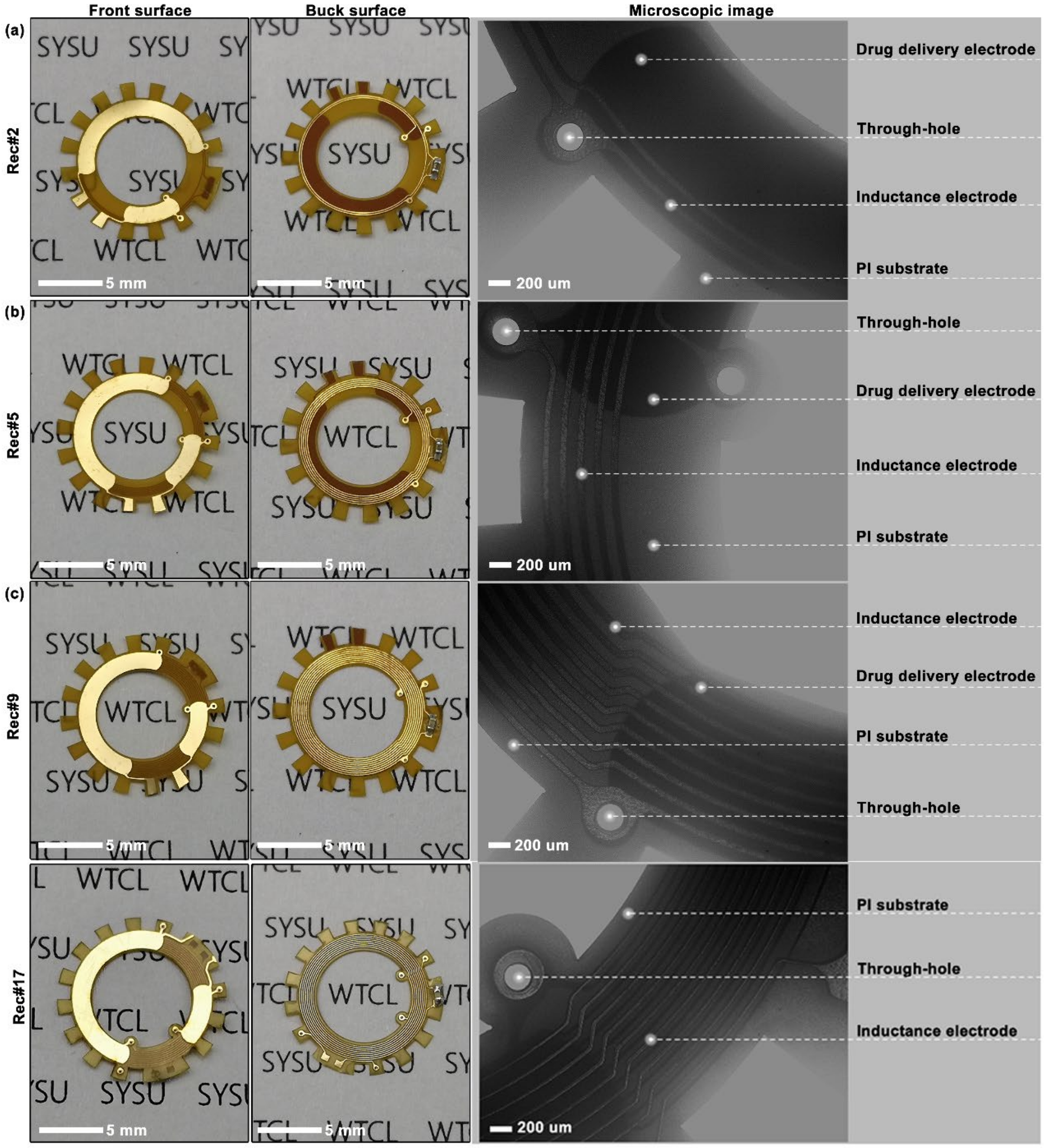

Supplementary Figure S13. Optical and microscopic images of four WPT receive circuits (Rec#2, Rec#5, Rec#9, Rec#17). These four types of WPT receivers with 2, 5,

9, and 17 coils-design (namely Rec#2, Rec#5, Rec#9, and Rec#17, respectively) were designed to achieve optimal performance coupled with transmitter.

Table S4. Detailed parameters and size of four WPT receiver circuits.

	Rec#2	Rec#5	Rec#9	Rec#17
Number of layers	2	2	2	3
Number of coil turns	2	5	9	17
Width of the inductance electrode (μm)	60	60	60	60/30
Space of the inductance electrode (μm)	90	90	90	90/120
The inner diameter of inductance coil (mm)	9.44	8.54	7.34	7.82
The outer diameter of inductance coil (mm)	9.86	9.86	9.86	10.43
Parasitic resistance (Ω)	1.4	2.9	4.9	22.1
Capacitor matched with inductance coil (nF)	82	39	22	3.3
Quality Factor	1.63	1.66	1.74	2.57

Table S5. Detailed parameters and size of WPT transmitter circuits.

Inductor (μH)	Capacitor matched with inductance coil (pF)	Parasitic resistance (Ω)
180	196	2.9

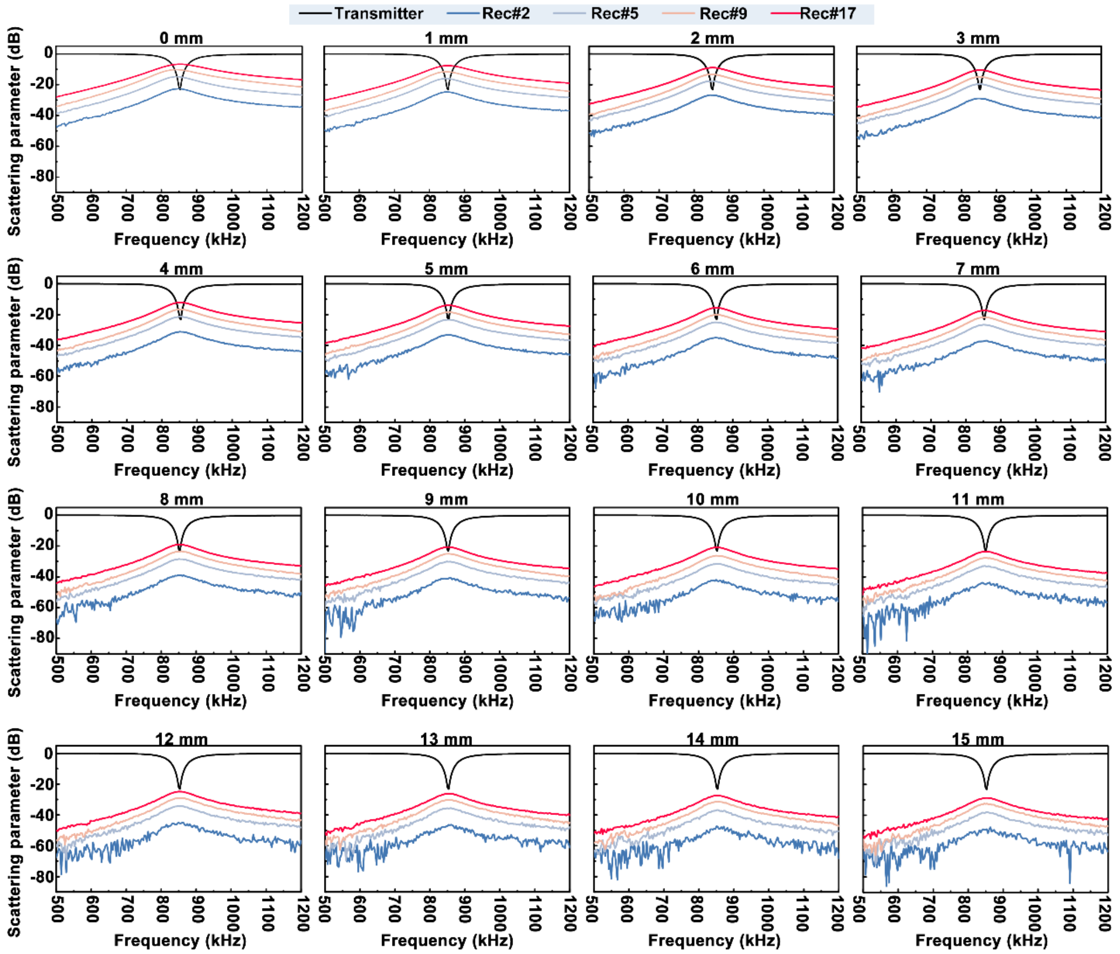

Supplementary Figure S14. The influence of radiation distanced (0 mm to 15 mm with step of 1 mm) on scattering parameter spectra between the WPT coil/four receivers. In these experiments, the integrated antenna and WTCL, aligned with identical axis, were connected with network analyzer. The results of all receivers showed that the resonant frequency of transmitter and all receivers remain at 850 kHz. Insert loss (S21) of all receivers revealed that S21 of Rec#17 was significantly higher than other circuits under identical radiation distance. It indicated that the Rec#17 was better matched with the WPT transmitter compared to other receive circuits. Hence, the combination of transmitter and Rec#17 possessed the potential to provide optimized electric power for iontophoretic delivery.

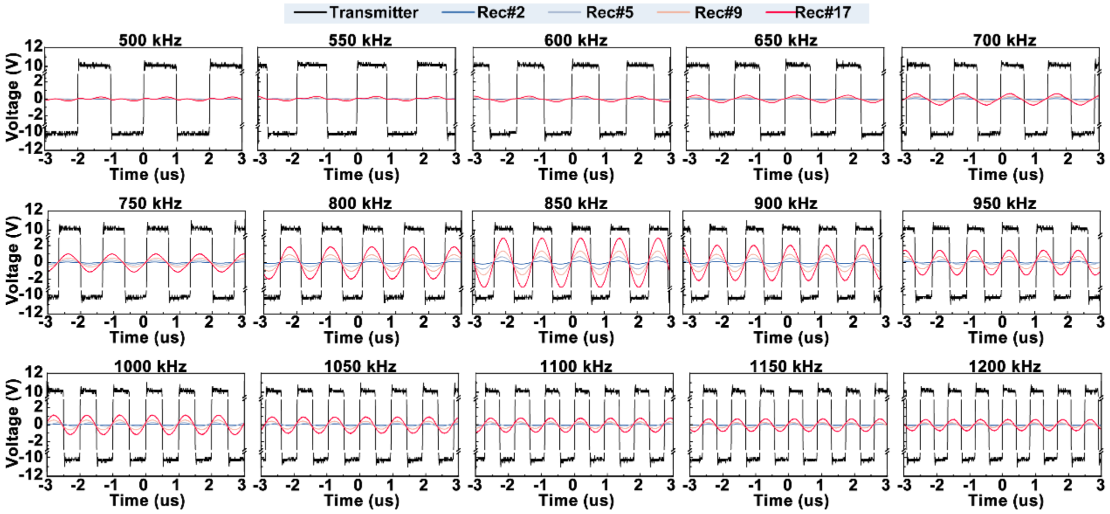

Supplementary Figure S15. The influence of frequencies (500 KHz to 1200 KHz with step of 50 KHz) on wireless voltage supply performance of four WPT systems. In these tests, the integrated antenna and WTCL, aligned with identical axis, were connected with waveform generator and oscilloscope, respectively. The distance between integrated antenna and WTCL was set at 6 mm. Sequentially, a series of square wave with different frequency (500 kHz to 1200 kHz with step of 50 kHz) were exerted on transmitter. Then the collected voltage of four receivers were recorded by oscilloscope in turn. Results shown that all of receivers exhibit higher values of voltage peak to peak (Vpp) at 850 kHz.

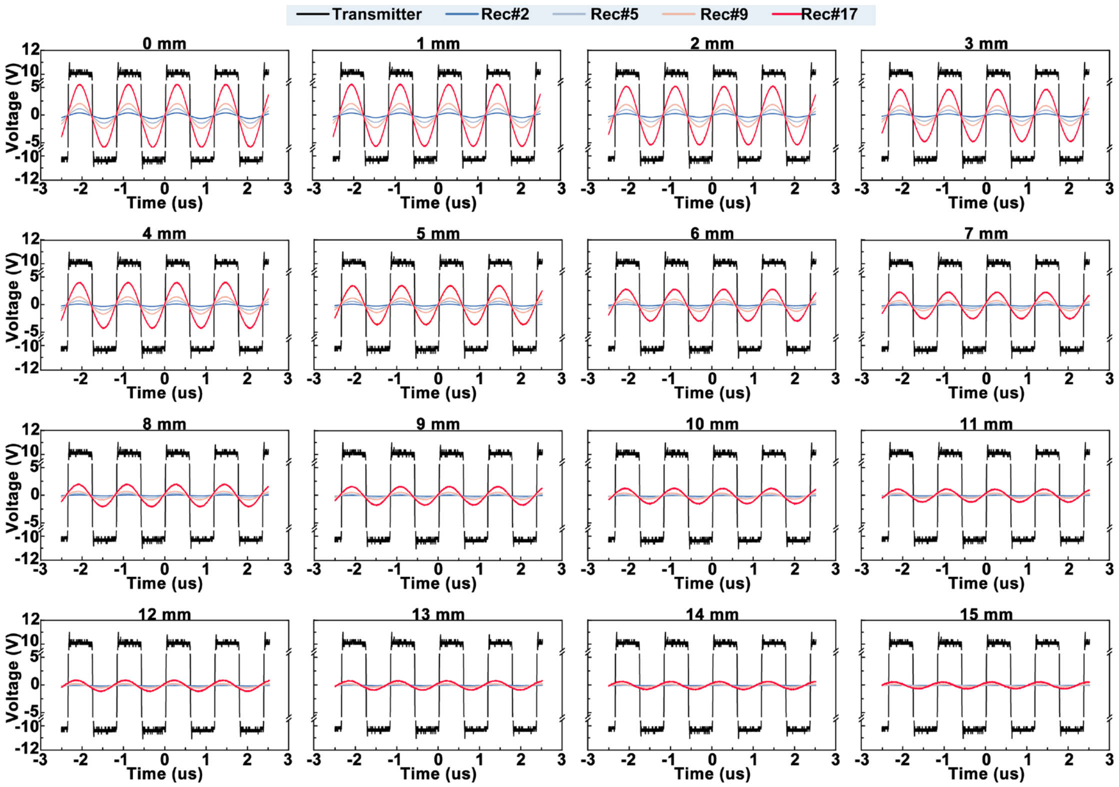

Supplementary Figure S16. The influence of radiation distances (0 mm to 15 mm with step of 1 mm) on wireless voltage supply performance of four WPT systems. In these tests, the integrated antenna and WTCL, aligned with identical axis, were connected with waveform generator and oscilloscope, respectively. The square voltage wave exerted on transmitter of integrated antenna was kept with 20 Vpp at 850 kHz. In these tests, the Rec#17 exhibited higher voltage compared to the other three circuits under conditions of same radiation distance.

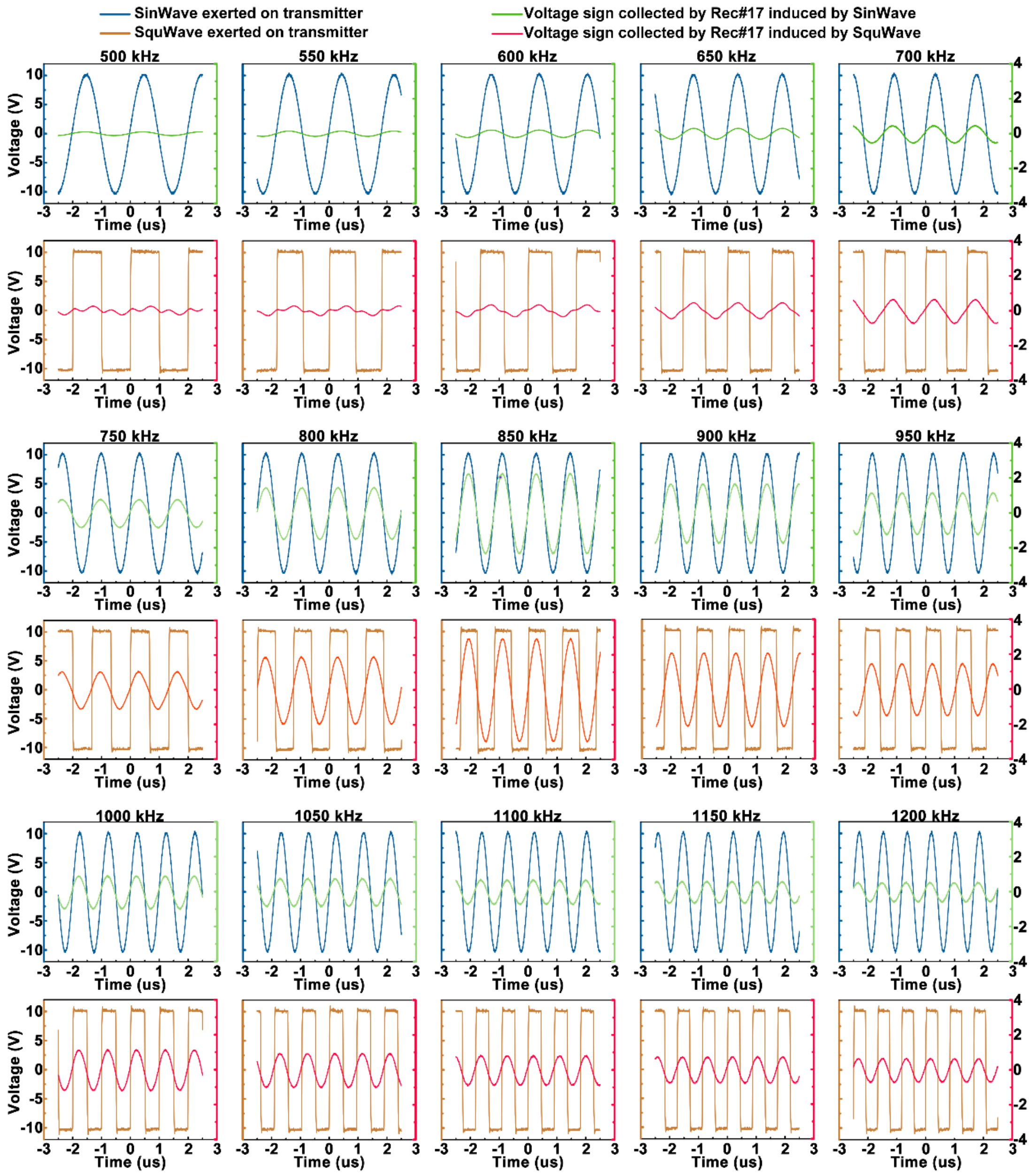

Supplementary Figure S17. The influence of square wave and sine wave's frequencies (20 Vpp, 500 KHz to 1200 KHz with step of 50 KHz) on the performance of wireless power supply via WPT systems (Transmitter and Rec#17 with the radiation distance of 6 mm). In these tests, the integrated antenna and WTCL, aligned with identical axis, were connected with waveform generator and oscilloscope, respectively. The distance between integrated antenna and WTCL was kept at 6 mm. These figures reveal that 850 kHz remain was the optimal frequency for the WPT

system consist of the Rec#17 and transmitter to acquire higher voltage regardless of voltage waveform.

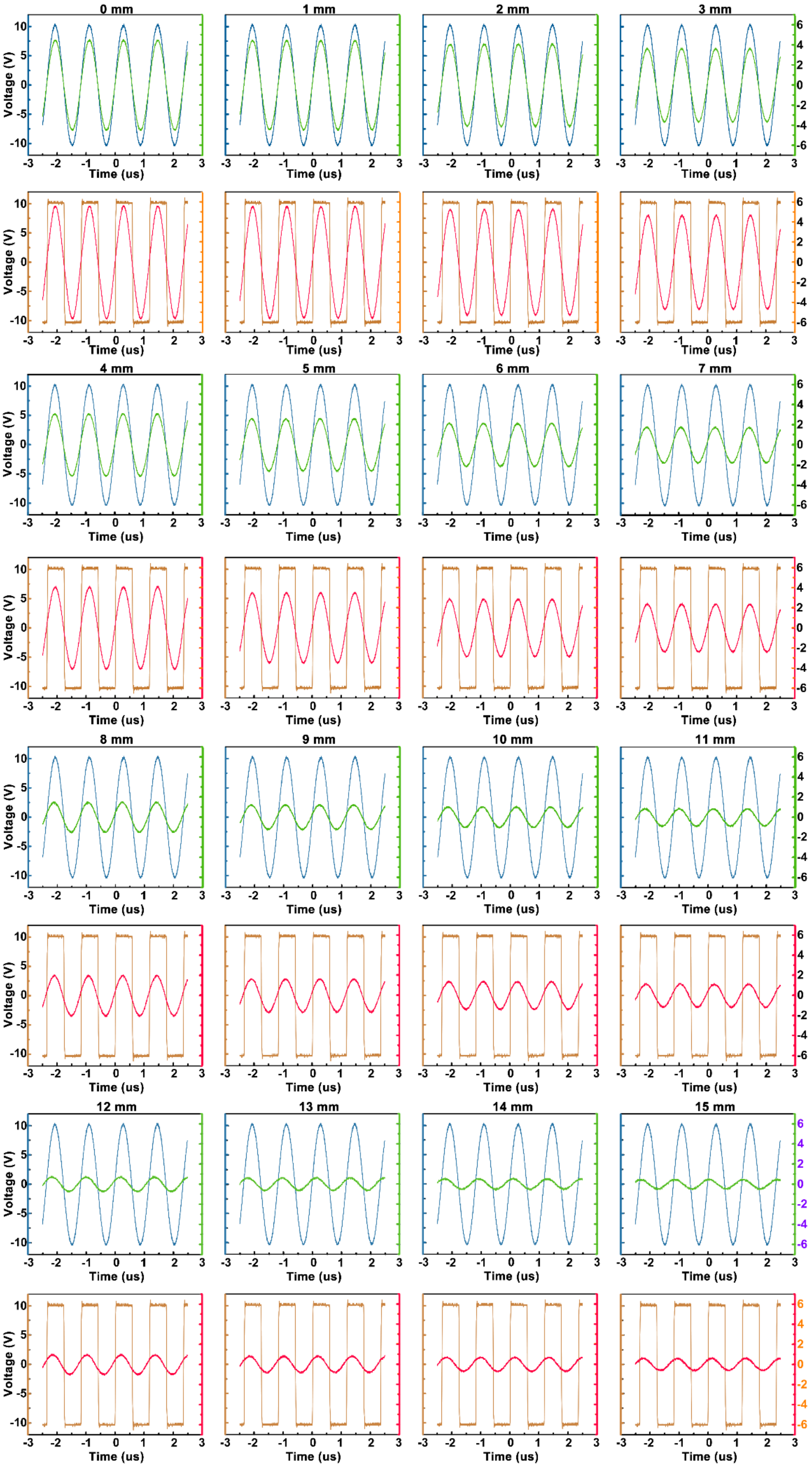
0 mm
1 mm
2 mm
3 mm
4 mm
5 mm
6 mm
7 mm
8 mm
9 mm
10 mm
11 mm
12 mm
13 mm
14 mm
15 mm
Voltage (V)
Time (us)

Supplementary Figure S18. The influence of radiation distances (0 mm to 15 mm with step of 1 mm) on wireless power supply performance of the fourth WPT systems (Transmitter and Rec#17). In these tests, the integrated antenna and WTCL, aligned with identical axis, were connected with waveform generator and oscilloscope, respectively. The voltage wave exerted on transmitter of integrated antenna was set at 20 Vpp and 850 kHz. Results shown that Vpp of Rec#17 induced by the sine voltage wave exerted on transmitter was slightly lower than the Vpp via SquWave. It further demonstrated that the square voltage wave exerted on transmitter possessed the potential to provide higher transferred voltage in receiver circuit.

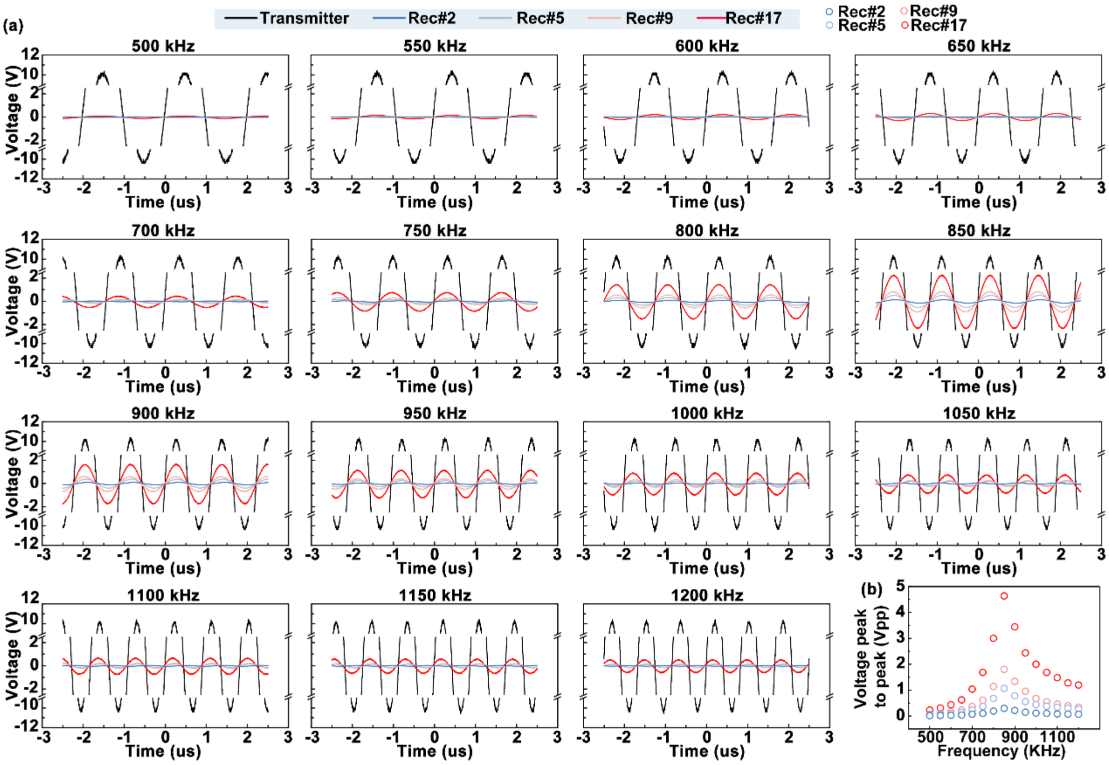

Supplementary Figure S19. (a) Vpp recorded from different receivers induced by sine voltage (20 Vpp) with different frequencies exerted on transmitter. In these tests, the integrated antenna and WTCL, aligned with identical axis, were connected with waveform generator and oscilloscope, respectively. The distance between integrated

antenna and WTCL was set at 6 mm. (b) The Vpp were plotted as a function of frequency. Similar to the activation by square wave voltage, the acquired Vpp on receiver circuit induced by transmitter with sine wave were also maximized at 850 kHz. The results reveal that 850 kHz was the optimal operation frequency of these receiver circuits for supplying higher Vpp.

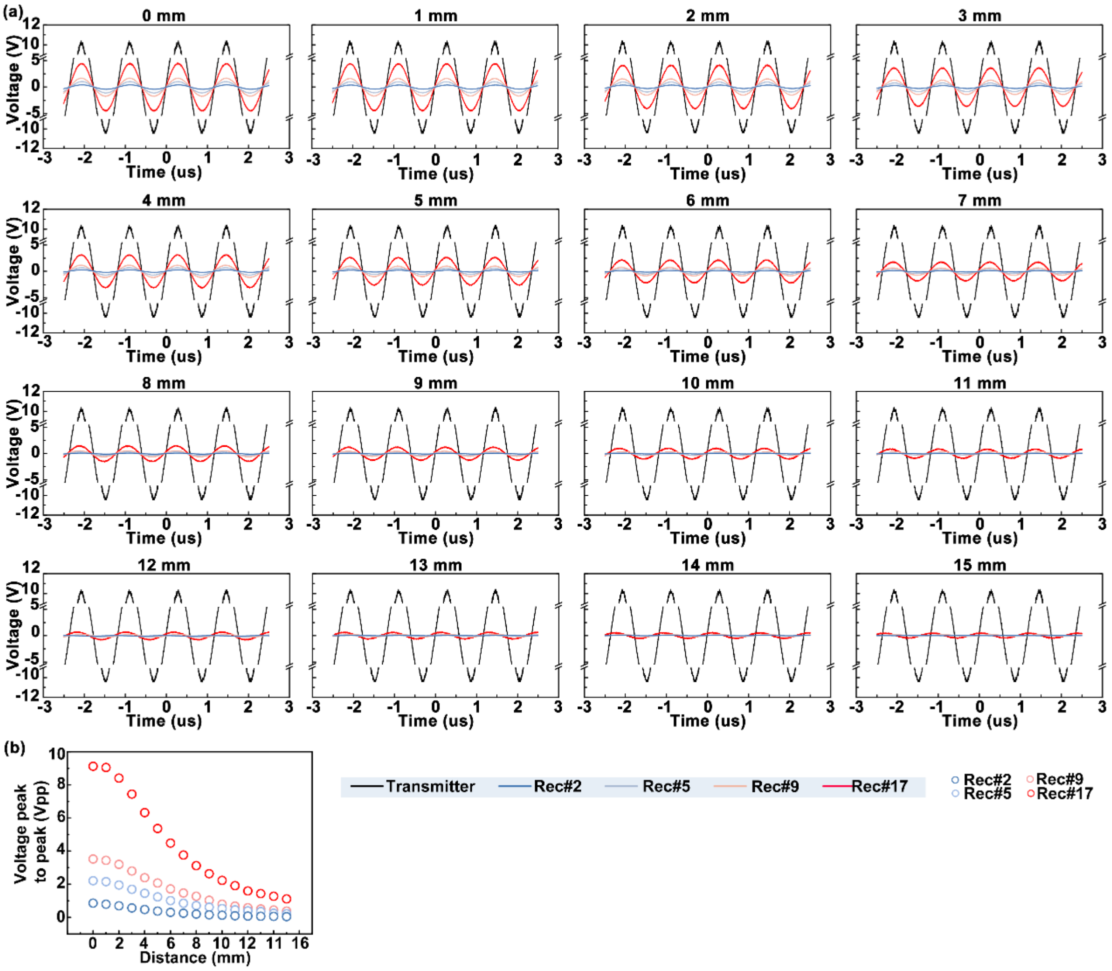

Supplementary Figure S20. (a) Vpp recorded from different receivers induced by sine voltage applied on transmitter under different radiation distances (0 to 15 mm with the step of 1 mm). In these tests, the integrated antenna and WTCL, aligned with identical axis, were connected with waveform generator and oscilloscope, respectively. The voltage wave exerted on transmitter of integrated antenna was kept with 20 Vpp at 850 kHz. (b) The Vpp values were plotted as a function of radiation distance.

S6. Theoretical calculations for the performance of WPT groups.

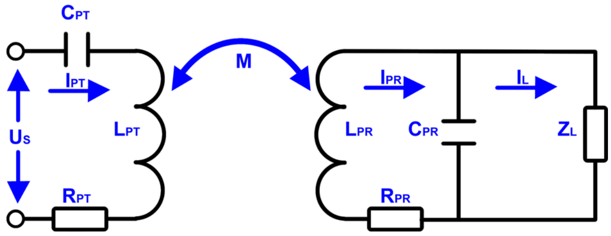

Supplementary Figure S21. Schematic circuit for the derivation of the power transfer efficiency equation. U_s, R_{PT}, L_{PT}, C_{PT}, I_{PT} refer to the alternating voltage supplied for the transmitter, parasitic resistance, inductor, capacitor and alternating current in transmitter. Correspondingly, R_{PR}, L_{PR}, C_{PR}, R_L denote the parasitic resistance, inductor, capacitor and electric load in receiver circuit. I_{PR} represents the total alternating current in receiver circuit. I_L is the alternating current flow through electric load.

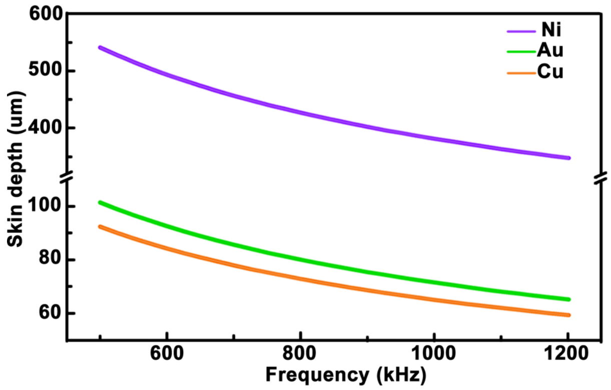

Supplementary Figure S22. Calculated skin depth of conductor composited of Ni, Au, and Cu in the frequency range from 500 kHz to 1.2 MHz. Detailed parameters (relative magnetic permeability and conductivity of the conductive matter) were listed in Table S6. Calculation results show that the skin depth decreased gradually as the operation frequency increases from 500 kHz to 1.2 MHz. Moreover, the skin depth of Au, Cu, and Ni are 78.19 um, 71.23 um, and 415.19 um, respectively at alternating current in 850 kHz. The parameters of these conductive materials are thicker than its practical thickness in WPT receiver circuits. It indicated that alternating current with frequency of 850 kHz was distributed in the whole cross-section of the conductor,

which suggested almost negligible power loss caused by skin effect needed to be considered in the WPT subsystem.

Table S6. Relative magnetic permeability and conductivity of the conductive matter[16].

Conductive materials	Relative magnetic permeability μ_r	Conductivity σ (S/m)
Copper (Cu)	0.999900	5.88×10^7
Nickle (Ni)	120	1.44×10^4
Gold (Au)	0.999983	4.88×10^7

S7. Characterization of cross-coupling between IOP monitoring and WPT activated iontophoresis of the WTCL.

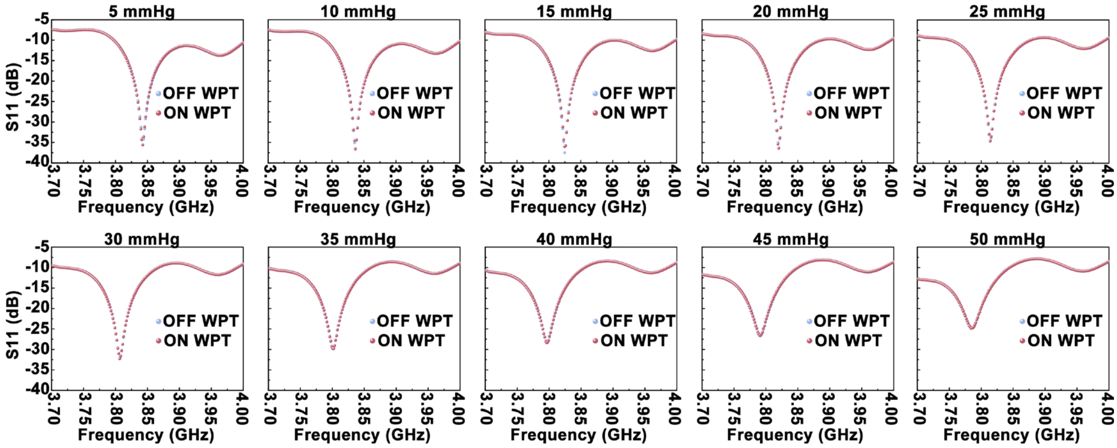

Supplementary Figure S23. The influence generated by the radiation of WPT transmitter to IOP sensing performance. The distance between integrated antenna and WTCL was set at 6 mm. Network analyzer was connected to the IOP reading coil of the integrated antenna to monitor the physiological pressure transduced by WTCL. Saline solution were injected into the anterior chamber of porcine eyes via a infusion needles (0.45×13.5 mm) controlled by syringe pump to achieve IOPs ranging from 5 to 50 mmHg with the step of 5 mmHg. During the process of IOP sensing, the power

generated by waveform generator for WPT transmitter was turned on and off to evaluate the S11 response of the IOP monitoring module.

S8. Theoretical simulations of iontophoretic medicines administration via COMSOL Multiphysics 5.5.

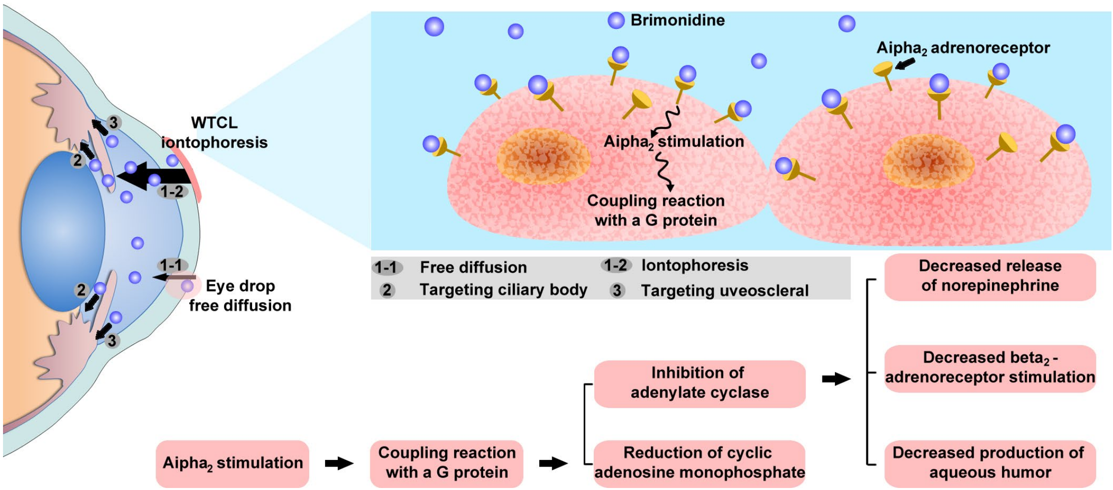

Supplementary Figure S24. Schematic of the action mechanism of brimonidine on reducing IOP.

Brimonidine as an alpha-adrenergic receptor agonist, a common medicine used clinically for the treatment of glaucomatous diseases. Topically applied brimonidine drops on the ocular surface are able to cross the cornea barriers, conjunctiva and sclera by free diffusion into the atrial water of the anterior chamber of the eye (process 1-2). This positively charged brimonidine drug can efficiently cross the corneal barrier into the anterior chamber of the eye driven by iontophoresis (process 1-1) in a relatively short period of time. Brimonidine in the atrial water would bind to the α2 adrenergic receptors present in abundance in the ciliary epitheliumresults (process 2) in a coupling reaction with a G protein, resulting in inhibition of adenylate cyclase and reduction of cyclic adenosine monophosphate levels. In addition to reducing the production of atrial fluid, brimonidine also promotes the efflux of atrial fluid through the uveoscleral (process 3). The decrease in atrial fluid production will hinder the volume of atrial fluid that accumulates in the anterior chamber of the eye from rising further. In addition, as the atrial fluid flows out through the uveosclera, the

volume of atrial fluid in the anterior chamber of the eye will gradually decrease. As a result, the intraocular pressure (IOP) of the eye gradually decreases.

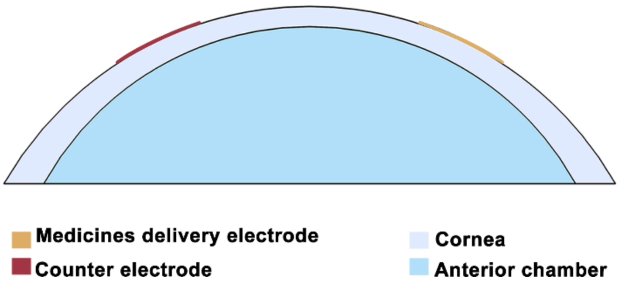

Supplementary Figure S25. Sectional view of 3D model of ocular drug delivery with the structures and components imitating the actual scenario, which was simulated using COMSOL Multiphysics 5.5 to evaluate the feasibility of enhanced trans-corneal drug delivery by the iontophoretic electrode on WTCL.

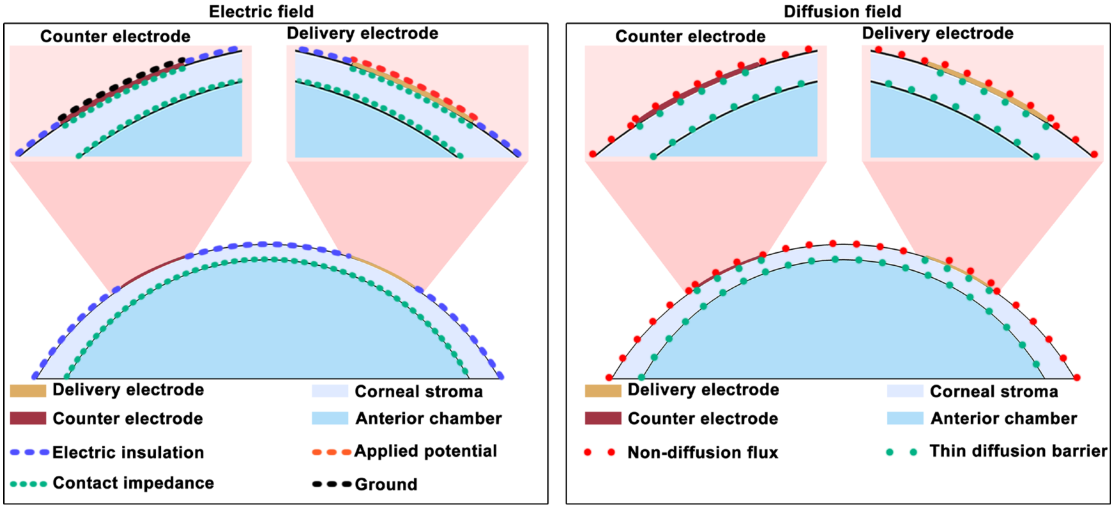

Supplementary Figure S26. Boundary condition of the COMSOL delivery model. Anterior segment of the eye was modeled as aqueous humor, cornea including epithelial cell, stroma, and endothelial cell layer. The cornea presented a diffusion barrier for the drug molecules. The total drug amount in this system was set as constant, initially 100% loaded in the hydrogel. The average concentration of the drugs in the aqueous humor volume was quantified to evaluate the delivery efficiency.

Table S7. Detailed parameters of COMSOL simulations for transcorneal delivery of brimonidine.

Components	Symbol	Value	Definition
pHEMA hydrogel	Hh	50 μm	The thickness of pHEMA hydrogel.
pHEMA hydrogel	σh	0.2 S/m	The electrical conductivity of pHEMA hydrogel.
pHEMA hydrogel	Dh	0.1	The relative diffusivity of brimonidine in pHEMA hydrogel.
pHEMA hydrogel	Ap	16.98 mm^2	The area of pHEMA hydrogel
pHEMA hydrogel	Cg0	1 $mmol/m^3$	Medicinesof brimonidine concentration in hydrogel
Counter electrode	An	4.78 mm^2	The area of counter electrode
Corneal stroma	σc[17]	0.2 S/m	The electrical conductivity of corneal stroma.
Corneal stroma	Dc	0.1	The relative diffusivity of brimonidine in corneal stroma.
Corneal stroma	Hst	300 μm	The thickness of corneal stroma.
Corneal epithelial cell layer	Hepc	100 μm	The thickness of corneal epithelial cell layer (modeled as the top boundary of the corneal layer).
Corneal epithelial cell layer	σepc	0.021 S/m	The electrical conductivity of corneal epithelial cell layer.
Corneal epithelial cell layer	Depc	0.01	The relative diffusivity of brimonidine in corneal epithelial cell layer.
Corneal endothelial cells layer	Hepe	100 μm	The thickness of corneal endothelial cells layer (modeled as the bottom boundary of the corneal layer).
Corneal endothelial cells layer	σepe	0.021 S/m	The electrical conductivity of corneal endothelial cells layer.
Corneal endothelial cells layer	Depe	0.01	The relative diffusivity of brimonidine in corneal endothelial cells layer.
Aqueous humor	σa	2.1 S/m	The electrical conductivity of aqueous humor.
Brimonidine	Da	1E-9 m^2/s	The diffusivity of brimonidine in aqueous humor.
Brimonidine	Z	2	Charge of brimonidine

Electric potential	U0	Variable

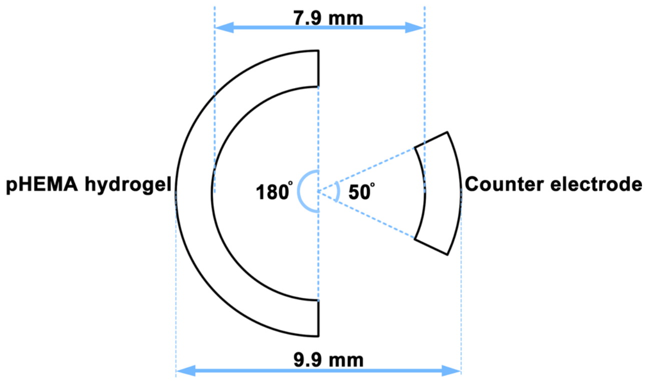

Supplementary Figure S27. The CAD diagram showing the 2D dimensions of iontophoretic electrode in the simulation model. Counter electrode and pHEMA hydrogel that served as reservoir to load bio-active compounds were attached on cornea. Furthermore, the back surface of hydrogel was attached to the drug delivery electrode. The counter electrode was labeled with ground in electric field.

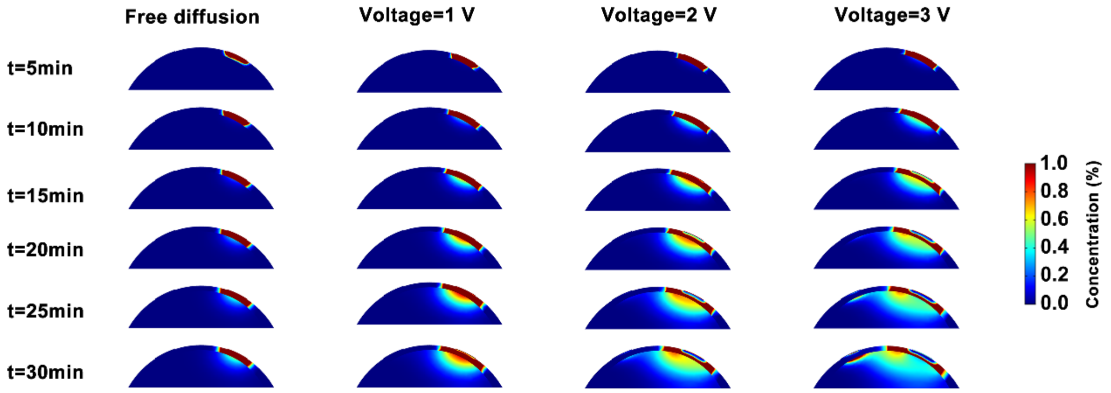

Supplementary Figure S28. Illustration of the diffusion profile of drug molecules in the anterior region of the eye after iontophoresis at 0, 1, 2 and 3 V for t=5 min to 30 min with the step of 5 min via WTCL. Under constant electrical voltages, the electrode generated electric field across cornea. For drug delivery, the initial drug concentration was set as Cg0 in hydrogel. Drug molecules were driven into aqueous humor across corneal barriers by the effect of electrophoresis under the means of this

electric field. The average drug concentration in the anterior chamber (aqueous humor) was calculated to evaluate the medicines administration efficiency.

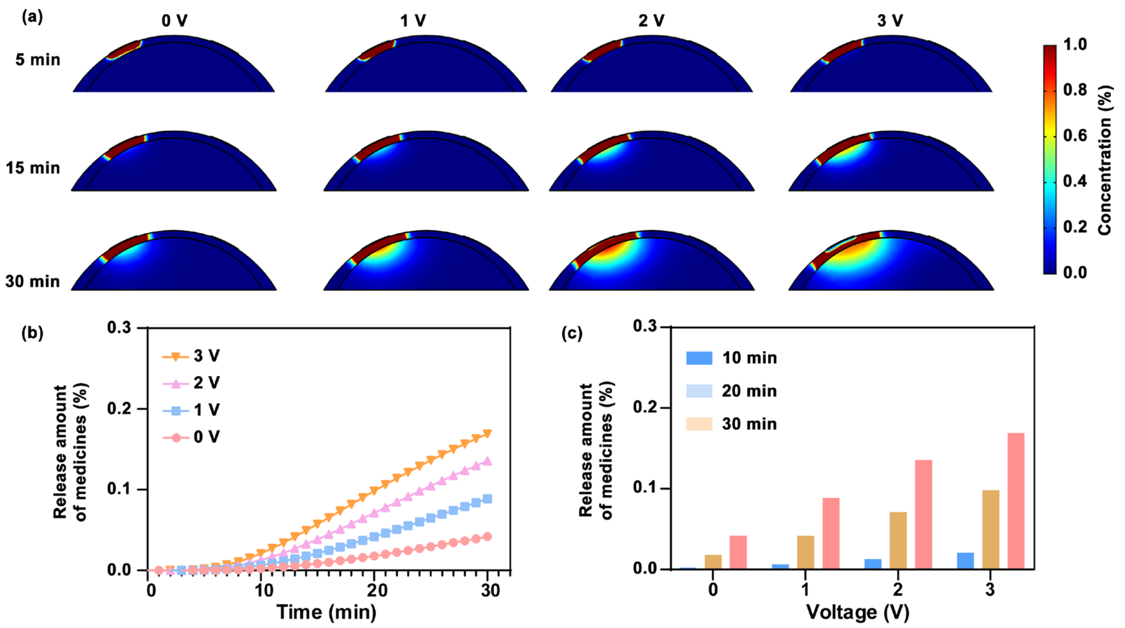

Supplementary Figure S29. (a) The time slots of Rhodamine B concentration profile delivered by WTCL at various applied voltages (0, 1, 2, and 3 V). The delivered amounts of drugs at different conditions, including the (a) applied voltages, (b) iontophoretic duration.

For molecules that are difficult to observe or characterize directly, many studies often use fluorescent molecules to mimic drug molecules to show the distribution of drugs in tissues. [*Sci. Adv. 2020; 6 : eaba3252*] Rhodamine B has a relative molecular mass of 479.01 and is a positively charged red fluorescent dye, while brimonidine tartrate has a relative molecular mass of 422.22 and is also a small positively charged molecule. Thus, Rhodamine B has similar molecular weight and charge properties as brimonidine tartrate, and theoretically they would possess similar kinetics to penetrate cornea. In this work, we deliver the fluorescent Rhodamine B to visually demonstrate that iontophoresis can facilitate the transport of charged molecules across the cornea. Brimonidine tartrate theoretically has slightly better performance than Rhodamine B in penetrating the corneal layer under iontophoresis since it is divalent charged. We have already developed a COMSOL simulation model in manuscript to study the penetration of brimonidine tartrate into the anterior region under iontophoresis. We further supplemented the COMSOL model to simulate the penetration of Rhodamine B (molecular weight 479, charge +1 valence) through the corneal layer as shown in Figure R1-5. Based on the results, it can be observed that Rhodamine B and brimonidine tartrate possessed similar diffusion behavior under iontophoresis.

Table S8. Detailed parameters of COMSOL simulations for transcorneal delivery of Rhodamine B.

Components	Symbol	Value	Definition
pHEMA hydrogel	Hh	50 µm	The thickness of pHEMA hydrogel.
pHEMA hydrogel	σh	0.2 S/m	The electrical conductivity of pHEMA hydrogel.
pHEMA hydrogel	Dh	0.1	The relative diffusivity of brimonidine in pHEMA hydrogel.
pHEMA hydrogel	Ap	16.98 mm^2	The area of pHEMA hydrogel
pHEMA hydrogel	Cg0	1 $mmol/m^3$	Medicinesof brimonidine concentration in hydrogel
Counter electrode	An	4.78 mm^2	The area of counter electrode
Corneal stroma	σc[17]	0.2 S/m	The electrical conductivity of corneal stroma.
Corneal stroma	Dc	0.1	The relative diffusivity of brimonidine in corneal stroma.
Corneal stroma	Hst	300 µm	The thickness of corneal stroma.
Corneal epithelial cell layer	Hepc	100 µm	The thickness of corneal epithelial cell layer (modeled as the top boundary of the corneal layer).
Corneal epithelial cell layer	σepc	0.021 S/m	The electrical conductivity of corneal epithelial cell layer.
Corneal epithelial cell layer	Depc	0.01	The relative diffusivity of brimonidine in corneal epithelial cell layer.
Corneal endothelial cells layer	Hepe	100 µm	The thickness of corneal endothelial cells layer (modeled as the bottom boundary of the corneal layer).
Corneal endothelial cells layer	σepe	0.021 S/m	The electrical conductivity of corneal endothelial cells layer.
Corneal endothelial cells layer	Depe	0.01	The relative diffusivity of brimonidine in corneal endothelial cells layer.
Aqueous humor	σa	2.1 S/m	The electrical conductivity of aqueous humor.
Brimonidine	Da	1E-9 m^2/s	The diffusivity of brimonidine in aqueous humor.
Brimonidine	Z	1	Charge of brimonidine
Electric potential	U0	Variable	

S9. Supporting information for in vivo experiments.

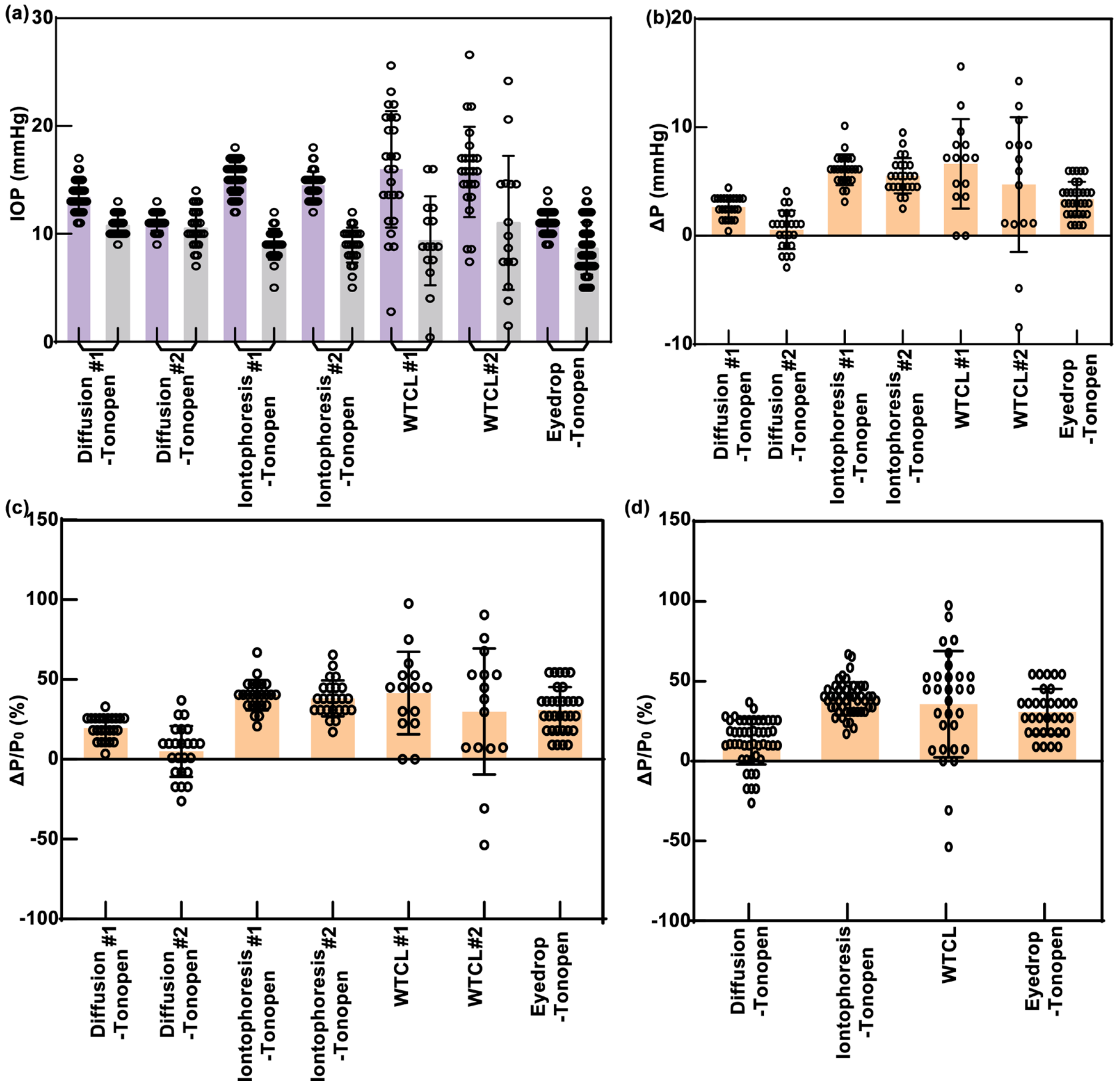

Supplementary Figure S30. Assessment of IOP reduction effect after 0.5 hour of drug administration, summarizing based on Figure 6e-6h in main text. (a) Analysis of the measured IOP value (either by WTCL or TonoPen) recorded in the different period of experiments. The purple columns refer to the IOP data in the initial period (in 1 hour) before drug delivery. The gray columns represent the IOP data recorded right (in 0.5 hour) after drug delivery. From left to right, N=40, 24, 40, 24, 40, 24, 40, 24, 25, 15, 25, 15, 88, 48 data points. (b) IOP changes (ΔP) at different delivery experiments. ΔP could be expressed as: $\Delta P = P - P_{0Avg}$, where P denotes the real-time IOP recorded in 0.5 hour after drug delivery, P_{0Avg} represents the average IOP before drug delivery. N=24, 24, 24, 24, 15, 15, 32 data points. (c) Normalized IOP changes ($\Delta P/P_{0Avg}$) in different drug delivery experiments. $\Delta P/P_{0Avg}$ could be expressed as:

$\Delta P/P_{0Avg} = (P - P_{0Avg}) * 100/P_{0Avg}$, where P denotes the real-time IOP recorded in 0.5 hour right after drug delivery, while P_{0Avg} represents the average IOP before drug delivery. N=24, 24, 24, 24, 15, 15, 32 data points. (d) Assessment of IOP reduction effect by different drug delivery methods. ΔP recorded from different experimental animal groups in the same delivery approach were averaged to calculate $\Delta P/P_{0Avg}$ for analyzing IOP reduction effect. N=48, 48, 30, 32 data points. All data were presented as mean ± s.d..

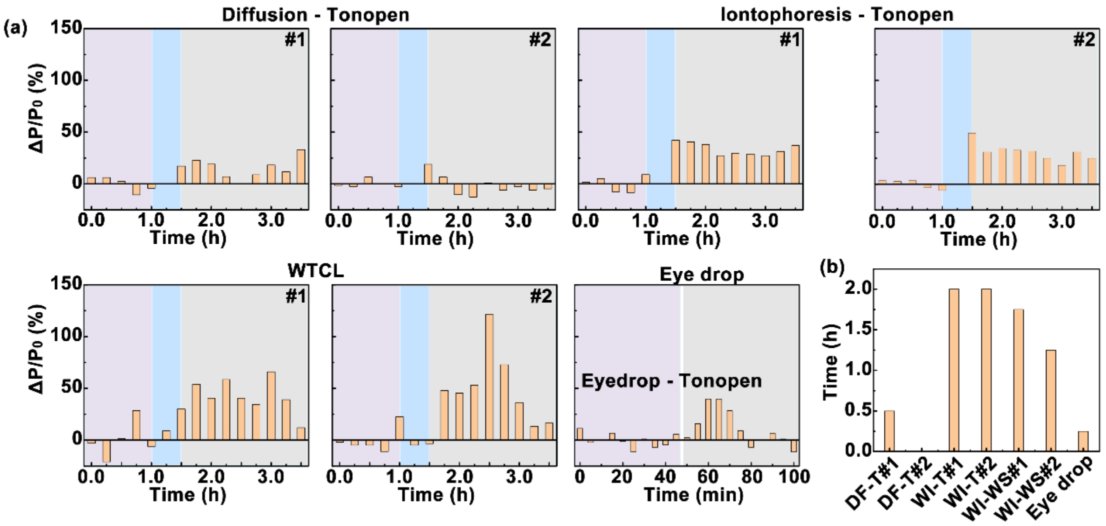

Supplementary Figure S31. Analysis of the duration of IOP reduction. (a) $\Delta P/P_{0Avg}$ at each time point by different drug delivery approach. (b) Quantifications of IOP reduction duration by different drug delivery approach. $\Delta P/P_{0Avg} \geq 20\%$ was identified as the effective IOP regulation[18], and the period during which the IOP meet this criteria was defined as the effective IOP regulation period.

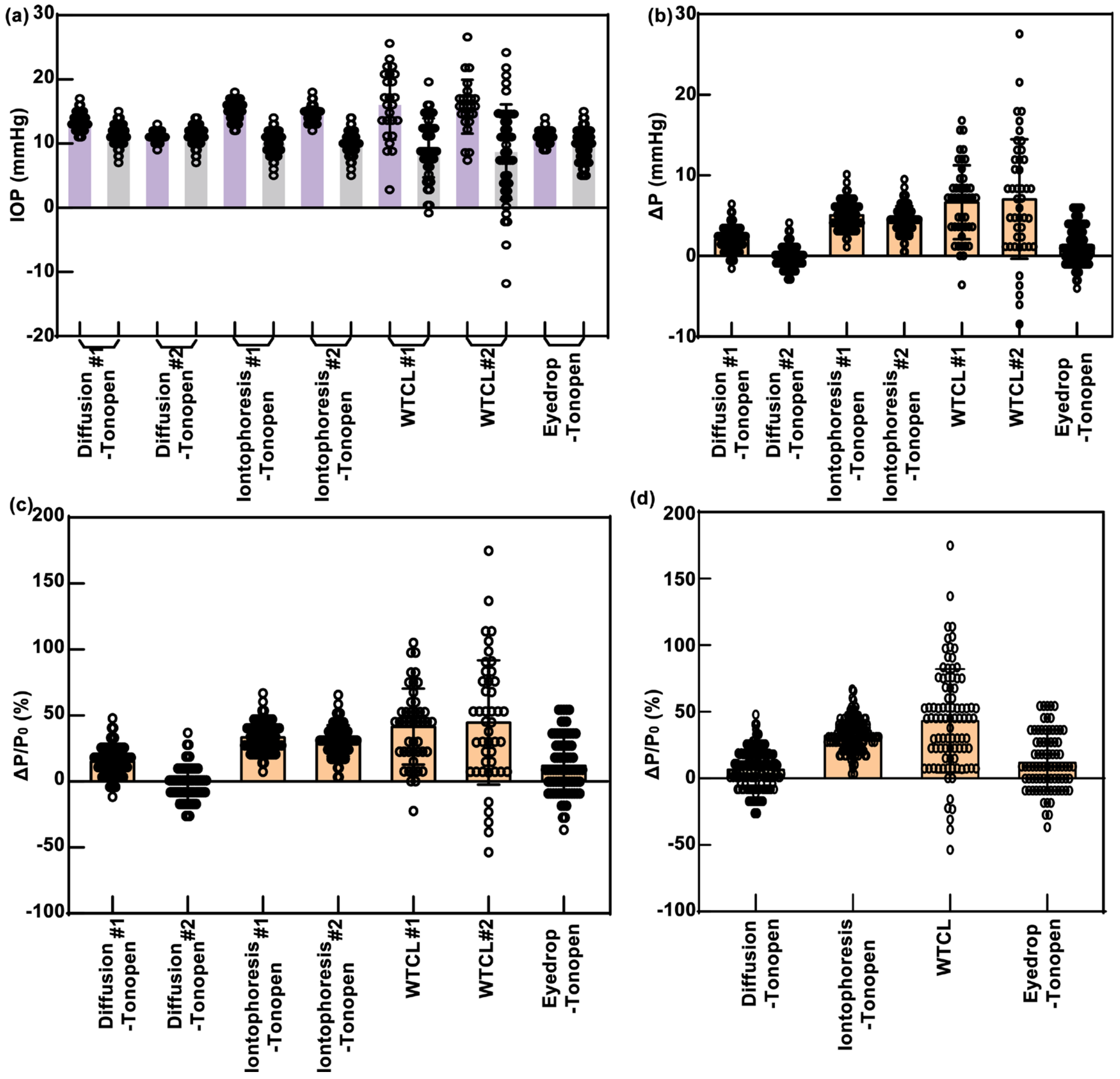

Supplementary Figure S32. Assessment of IOP reduction effect after 2 hour of drug administration, summarizing based on Figure 6e-6g in main text. And IOP reduction effect after 0.8 hour of drug administration, summarizing based on Figure 6h in main text. (a) Analysis of the measured IOP value (either by WTCL or TonoPen) recorded in the different period of experiments. The purple columns refer to the IOP data in the initial period (in 1 hour) before drug delivery. The gray columns represent the IOP data recorded in 2 hours after drug delivery. From left to right, N=40, 72, 40, 72, 40, 72, 40, 72, 25, 45, 25, 45, 87, 80 data points. (b) IOP changes (ΔP) at different delivery experiments. ΔP could be expressed as: $\Delta P = P - P_{0Avg}$, where P denotes the real-time IOP recorded in 2 hours after drug delivery, P_{0Avg} represents the average IOP before drug delivery. From left to right, N=72, 72, 72, 72, 45, 45, 80 data points. (c)

Normalized IOP changes ($\Delta P/P_{0Avg}$) in different drug delivery experiments. $\Delta P/P_{0Avg}$ could be expressed as: $\Delta P/P_{0Avg} = (P - P_{0Avg}) * 100 / P_{0Avg}$, where P denotes the real-time IOP recorded in 2 hours after drug delivery, while P_{0Avg} represents the average IOP before drug delivery. From left to right, N=72, 72, 72, 72, 45, 45, 80 data points. (d) Assessment of IOP reduction effect by different drug delivery methods. ΔP recorded from different experimental animal groups in the same delivery approach were averaged to calculate $\Delta P/P_{0Avg}$ for analyzing IOP reduction effect. From left to right, N=144, 144, 90, 80 data points. All data were presented as mean ± s.d..

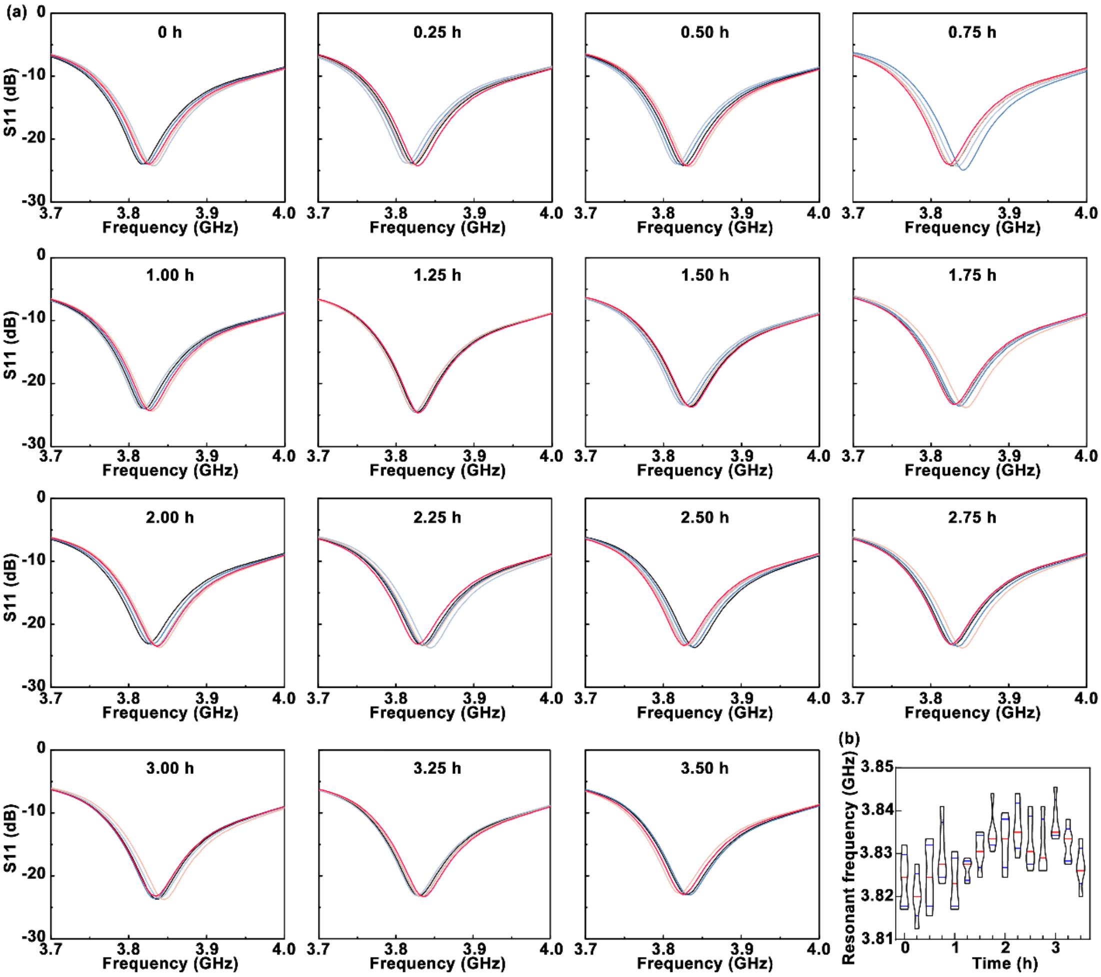

Supplementary Figure S33. (a) Scattering parameters of IOP sensing module at each measurement time point during the WTCL#1 experimental group in Figure 6g in main text. During synergistic IOP monitoring and in situ drug delivery by WTCL, the

scattering parameter of IOP monitoring module was recorded wirelessly by network analyzer every 15 minutes, while the resonant frequency measurements were repeated for 5 times at each time point. (b) Resonant frequency fluctuations as the function of time. The resonant frequency could be further converted to IOP, according to the standard curve of resonant frequency-IOP. N=8 measurements per group.

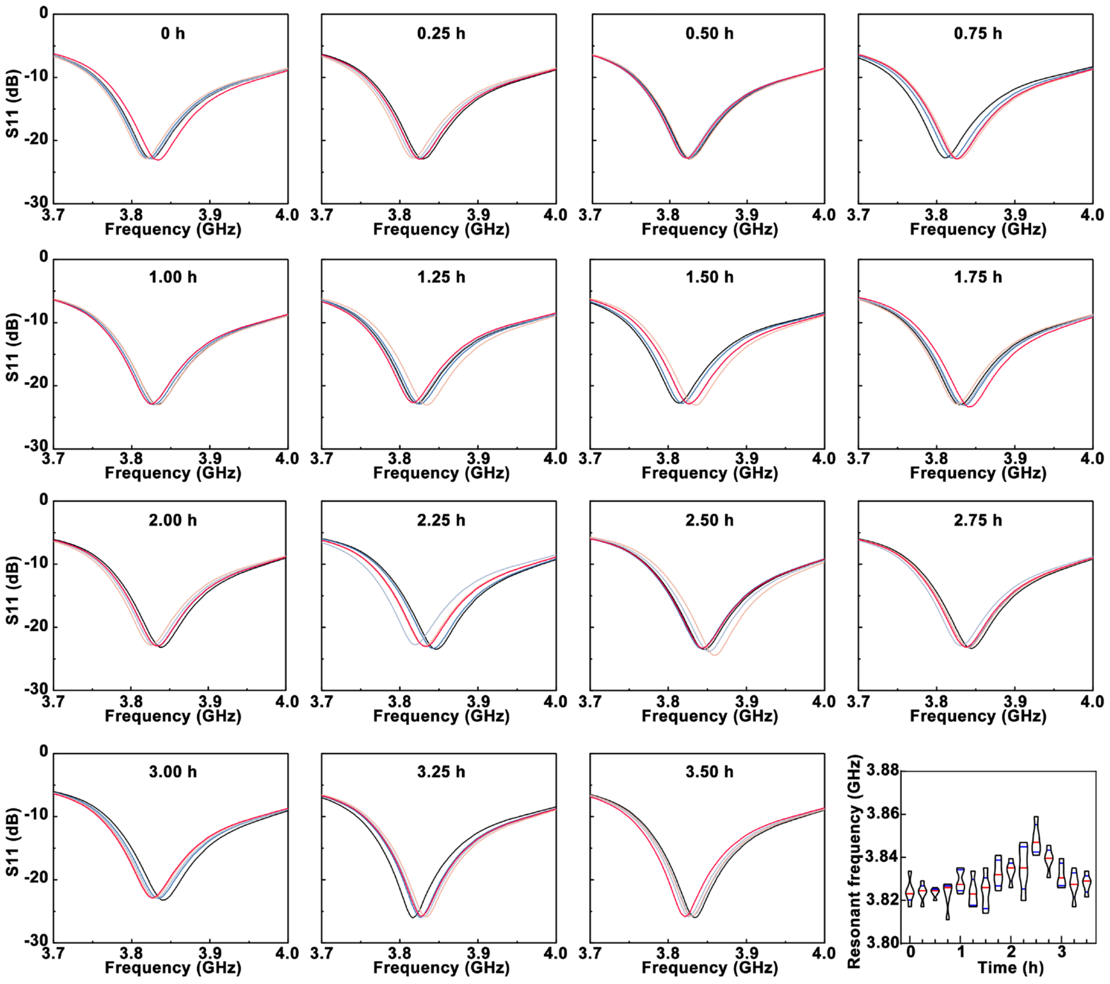

Supplementary Figure S34. (a) Scattering parameters of IOP sensing module at each measurement time point during the WTCL#2 experimental group in Figure 6g in main text. During synergistic IOP monitoring and in situ drug delivery by WTCL, the scattering parameter of IOP monitoring module was recorded wirelessly by network analyzer every 15 minutes, while the resonant frequency measurements were repeated for 5 times at each time point. (b) Resonant frequency fluctuations as the function of

time. The resonant frequency could be further converted to IOP, according to the standard curve of resonant frequency-IOP. N=8 measurements per group.

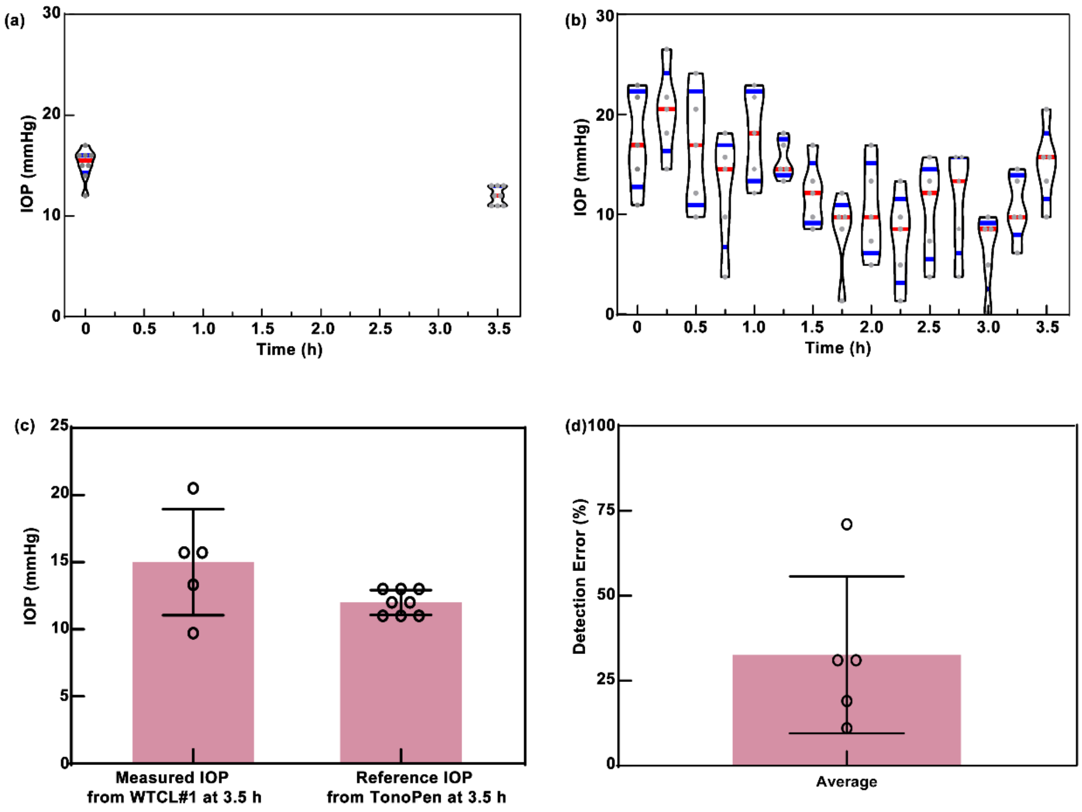

Supplementary Figure S35. (a) Reference IOP values, related to Figure 6g (I) in main text**,** captured by Tonopen before and after WTCL#1 experiment in Figure 6g in main text, which could be employed to calibrate the measured IOP and analyze detection error. N=8 measurements per group. (b) IOP values calculated from the resonant frequency values of WTCL#1 experiment in Supporting Information Figure S33, without calibration using the reference data via Tonopen measurement. N=8 measurements per group. (c) The measured IOP via WTCL#1 at the end of experiment (initially calibrated with the reference IOP provided by Tonopen), and the comparison to the reference IOP measured via Tonopen at the end of experiment. From left to right, N=5, 8 data points. (d) The detection error of IOP monitoring using WTCL with calibration. The detection error could be expressed as

$Error = |P - P_{Avg}| * 100\%/P_{Avg}$ where P represents the real-time pressure recorded by WTCL at 3.5 h, P_{Avg} denotes the average IOP collected by TonoPen at 3.5 h. N=5 data points. Data were presented as mean ± s.d..

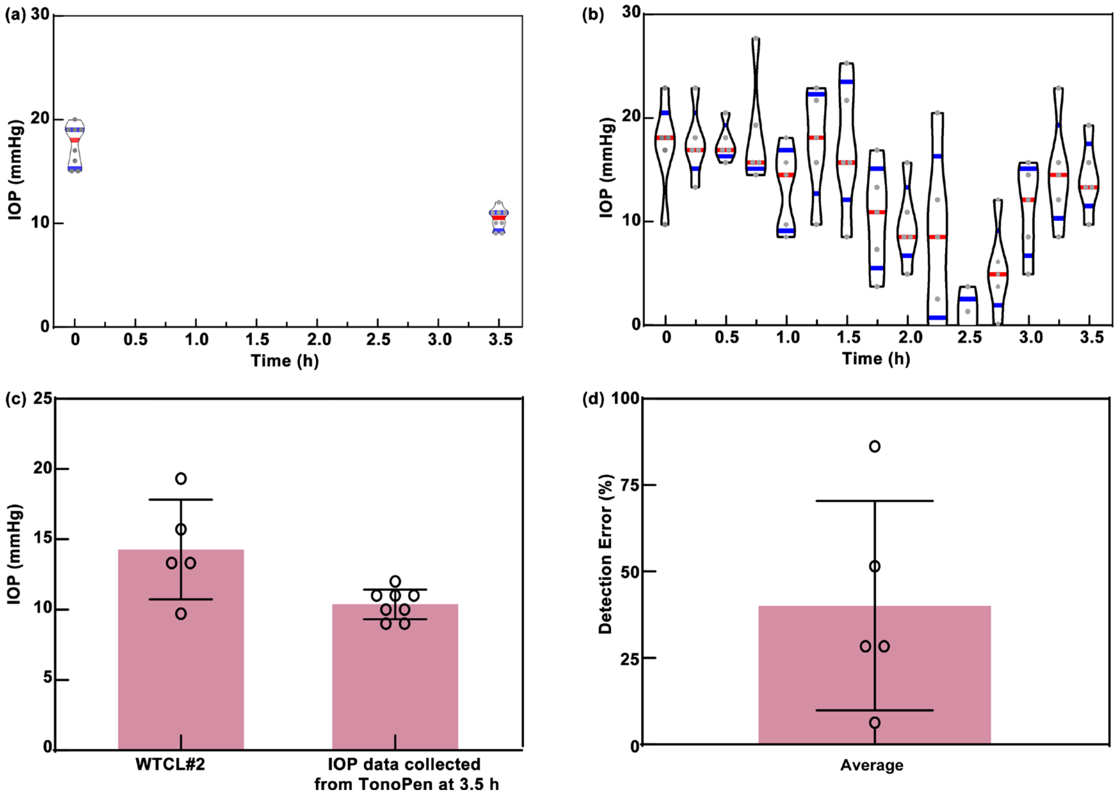

Supplementary Figure S36. (a) Reference IOP values, related to Figure 6g (II) in main text**,** captured by Tonopen before and after WTCL#2 experiment in Figure 6g in main text, which could be employed to calibrate the measured IOP and analyze detection error. N=8 measurements per group. (b) IOP values calculated from the resonant frequency values of WTCL#2 experiment in Supporting Information Figure S34, without calibration using the reference data via Tonopen measurement. N=8 measurements per group. (c) The measured IOP via WTCL#2 at the end of experiment (initially calibrated with the reference IOP provided by Tonopen), and the comparison to the reference IOP measured via Tonopen at the end of experiment. From left to right, N=5, 8 data points. (d) The detection error of IOP monitoring using WTCL with calibration. The detection error could be expressed as $Error = |P - P_{Avg}| * 100\%/P_{Avg}$ where P represents the real-time pressure recorded by

WTCL at 3.5 h, P_{Avg} denotes the average IOP collected by TonoPen at 3.5 h. From left to right, N=5 data points. Data were presented as mean ± s.d..

S10. Thermal analysis

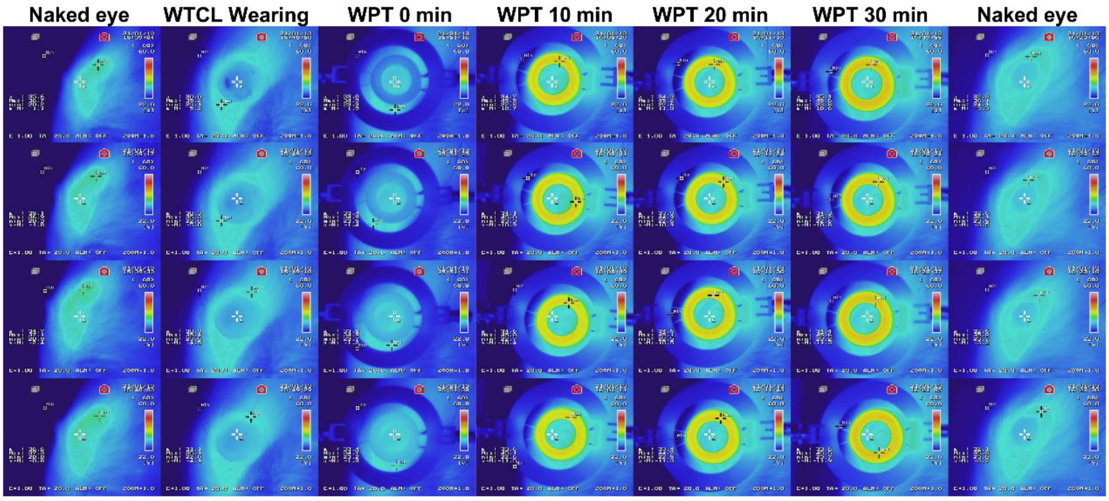

Supplementary Figure S37. Time slot infrared images showing the temperature variations of the ocular surface tissue, WTCL, and integrated antenna measured by infrared camera during the 30 min WPT process. WTCL was worn on rabbit's eye, and integrated antenna connected to waveform generator was posited above WTCL with the distance of 6 mm. Square voltage with 20 Vpp at 850 kHz produced from waveform generator was exerted on WPT transmitter. At each time point, 4 infrared images were recorded.

Reference:

1 Kessing;, S. V. & Thygesen;, J. *Primary angle-closure and angle-closure glaucoma*. (Kugler Publications, 2007).

2 Shingleton, B. J., Wadhwani, R. A., O'Donoghue, M. W., Baylus, S. & Hoey, H. Evaluation of intraocular pressure in the immediate period after phacoemulsification. *J. Cataract Refract. Surg.* **27**, 524-527, (2001).

3 Rainer, G. *et al.* Effect of dorzolamide and latanoprost on intraocular pressure after small incision cataract surgery. *J. Cataract Refract. Surg.* **25**, 1624-1629, (1999).

4 Carvalho, I. M. *et al.* Sustained drug release by contact lenses for glaucoma treatment-A review. *J. Control. Release* **202**, 76-82, (2015).

5 Kim, J. *et al.* Intraocular Pressure Monitoring Following Islet Transplantation to the Anterior Chamber of the Eye. *Nano Lett.* **20**, 1517-1525, (2020).

6 Kim, J. *et al.* Wearable smart sensor systems integrated on soft contact lenses for wireless ocular diagnostics. *Nat. Commun.* **8**, 14997, (2017).

7 Chen, G.-Z., Chan, I.-S. & Lam, D. C. C. Capacitive contact lens sensor for continuous non-invasive intraocular pressure monitoring. *Sens. Actuator A-Phys.* **203**, 112-118, (2013).

8 Chen, G.-Z., Chan, I.-S., Leung, L. K. K. & Lam, D. C. C. Soft wearable contact lens sensor for continuous intraocular pressure monitoring. *Med. Eng. Phys.* **36**, 1134-1139, (2014).

9 Kouhani, M. H. M., Weber, A. & Li, W. in *2017 IEEE 30th International Conference on Micro Electro Mechanical Systems (MEMS).* 557-560.

10 Kouhani, M. H. M., Wu, J., Tavakoli, A., Weber, A. J. & Li, W. Wireless, passive strain sensor in a doughnut-shaped contact lens for continuous non-invasive self-monitoring of intraocular pressure. *Lab Chip* **20**, 332-342, (2020).

11 Chen, P., Saati, S., Varma, R., Humayun, M. S. & Tai, Y. Wireless Intraocular Pressure Sensing Using Microfabricated Minimally Invasive Flexible-Coiled LC Sensor Implant. *J. Microelectromech. Syst.* **19**, 721-734, (2010).

12 Chen, P., Rodger, D. C., Saati, S., Humayun, M. S. & Tai, Y. Microfabricated Implantable Parylene-Based Wireless Passive Intraocular Pressure Sensors. *J. Microelectromech. Syst.* **17**, 1342-1351, (2008).

13 Crum, B. *Parylene Based Wireless Intraocular Pressure Sensor for Glaucoma Research*, Michigan State University, (2013).

14 Guimera, A., Ivorra, A., Gabriel, G. & Villa, R. Non-invasive assessment of corneal endothelial permeability by means of electrical impedance measurements. *Med. Eng. Phys.* **32**, 1107-1115, (2010).

15 Guimera, A. *et al.* A non-invasive method for an in vivo assessment of corneal epithelium permeability through tetrapolar impedance measurements. *Biosens. Bioelectron.* **31**, 55-61, (2012).

16 Zhang, Y. *et al.* High precision epidermal radio frequency antenna via nanofiber network for wireless stretchable multifunction electronics. *Nat. Commun.* **11**, 5629, (2020).

17 Klyce, S. D. Electrical profiles in the corneal epithelium. **226**, 407-429, (1972).

18 Jonas, J. B. *et al.* Glaucoma. *Lancet* **390**, 2183-2193, (2017).
